# Supplementary material for: Smoke-free status of homes and workplaces among Indian people: Evidence from Global Adult Tobacco SurveyData-2016/2017
Source: PLoS One. 2023 Feb 23;18(2):e0282138. doi: 10.1371/journal.pone.0282138 (PMC9949653; doi:10.1371/journal.pone.0282138)
Supplement: Attachment — Submitted filename: PlosOne SUNG reviewer 6-22-2021.pdf [file pone.0282138.s003.pdf]

REVIEWER'S REPORT
Manuscript ID: PONE-D-20-41034R1

Title: Smoke-free policies in the home and in the workplace among Indian people: Evidence from Global Adult Tobacco Survey Data-2016/2017

This revised paper still needs a substantial revision and editing.

1. There are published papers which used the same GATS-India-II data to examine the prevalence and correlates of SHS exposure in India (Tripathy 20201, 2020b). There is a need to discuss how this study differs from the published studies.
    a. I found some question about sample size. The study by Tripathy (2020) used all observations in the GATS-India-II (N=74,037) and identified 64,538 non-smokers and 9,499 current smokers (including 7,647 daily smokers and 1,852 less-than-daily smokers). After deleting missing values, this study included 71,046 for the analysis of smoke-free policies at home. However, there are 11,321 current smokers according to Table 1. Why the sample size of current smokers is larger than the sample size (11,321 vs. 9,499) in the study by Tripathy (2020) even though the total sample size is smaller (71,046 vs. 74,037)? I suspect that this study might use a different definition of current smokers.
    b. I also found some inconsistent findings. Tripathy (2020) reported that exposure to SHS in the home was 29.2% among all respondents and it was higher among females (30.4%) compared to males (28.1%); and that exposure to SHS in workplaces was 30% overall and it was higher among males (32.5%) compared to females (17.8%). However, this study yielded opposite results compared with Tripathy (2020).

    Tripathy JP. Secondhand smoke exposure at home and public places among smokers and non-smokers in India: findings from the Global Adult Tobacco Survey 2016-17. Environ Sci Pollut Res Int. 2020a;27(6):6033-6041. doi: 10.1007/s11356-019-07341-x.

    Tripathy JP. Smoke-free workplaces are associated with smoke-free homes in India: evidence for action. Environ Sci Pollut Res Int. 2020b;27(33):41405-41414. doi: 10.1007/s11356-020-10107-5.

2. (Page 2, abstract) The main aim of this study is to determine the correlates of smoke-free policies in the home and in the workplace. Before addressing the correlates, it is necessary to examine the prevalence of smoke-free policies in the home and in the workplace.

3. The second aim of this study is to assess the relationship between smoke-free policies and secondhand smoke (SHS) exposure. However, there is a lack of explanation for why examining this aim is important. Also, while the main aim of this study was analyzed using persons as the unit of the analysis, this aim was analyzed using states (30 states and 2 Union Territories) as the unit of the analysis. Moreover, while the main aim of this study was analyzed using the multivariable logistic regression model, this aim was analyzed using a simple Pearson's correlation without adjusting for other confounding factors. If the authors

really want to include this second aim in this paper, this aim should be examined in a much more rigorous way.

   a. First, the definition of the SHS exposure variables, including what GATS questions were used to measure SHS exposure, should be explained clearly in the Methods section.
   b. Second, a multivariable regression model which controls for other confounding factor should be used to analyze the association between smoke-free policies and SHS exposure.
   c. Third, justification is needed for why the analysis needs to be conducted at state-level rather than at person-level.

4. (Page 2, abstract). More specific results about the correlates of smoke-free policies need to be provide in the Results section. For example, what are the prevalence estimates for smoke-free policies among all people aged 15+ in India? How does the prevalence differ by sub-groups stratified by each correlate? What are the key findings concerning the adjusted odds ratios for the associations between correlates and smoke-free policies?

5. (Page 2, abstract). The Conclusion section needs to be re-written to highlight the key findings.

6. Page 3, 1st paragraph: "GATS-2 also reports that 38.7% … of exposure to secondhand smoke (SHS) takes place at home, 30.2% … at workplace and 23% … occurs at any public place". Before this sentence, it is important to let the readers know what was the prevalence of secondhand smoke exposure in India according to the GATS-2 data. Also, does this sentence mean that among people who were exposed to secondhand smoke, 38.7% were exposed at home, 30.2% were exposed at workplace, and 23% were exposed at public place. These 3 percentages only add up to 91.9%. What is the source of secondhand smoke exposure for the remaining 8.1%?

7. Page 3, 2nd paragraph "Although there is a progress of smoke-free policies … government still couldn't reach its ultimate goal." What is the ultimate goal of the National Tobacco Control Program launched in 2007-2008?

8. Page 3, 3rd paragraph.
   a. Delete the wording "To our best knowledge".
   b. In the sentence "However, most of these previous studies have been focused on the prevalence and predictors of tobacco use [11, 18-20]", the cited studies were not relevant because they are about secondhand smoke exposure rather than about tobacco use.
   c. In the sentence "A study also observed that a smoke-free workplace was … associated with SHS exposure at home [21]", Ref #19 can also be cited in addition to Ref #21.
   d. In the sentence "A number of countries or community … the presence of policies that restrict smoking in the workplace and home may vary by … status [19, 22]", Ref #19

should not be cited because it is about secondhand exposure rather than about smoke-free policies.

9. Page 4, Data paragraph, in the sentence "The analysis of smoking policies at workplace … but outside their home (adults)", "smoking policies" should be corrected into 'smoke-free policies". Also, were "adults" defined as people aged 18+ or aged 15+?

10. Page 4, Section 2.3:
    a. In the 1st sentence, delete "The status of" and "consisted of four categories".
    b. In the 2nd sentence, change the sentence "To determine the correlates of complete smoke-free policies at home and workplace classified into binary in the following ways " to "Smoke-free policies at home and workplace were classified into binary in the following ways".
    c. If the outcome variable "smoke-free policies at home" is classified as a binary variable as stated in this section, why was this variable classified into a 4-group categorical variable in Table 1? The chi-square test for the association between 2-category smoke-free variable and the independent variable would have different results from the chi-square test for the association between 4-category smoke-free variable and the independent variable.

11. Page 5, 1st paragraph: The entire description of the independent variables was provided only in a supplementary table. Because the main aim of this study is to determine whether the selected independent variables are significant correlates of smoke-free policies, the independent variables should be explained in great detail in the Methods section including their names, definitions, and how they were measures. For example, for the smoking status variable, how were "smokers" defined? What GATS questions were used to define "smokers"? For the "knowledgeable of the children illness", what GATS questions were used to define this variable?

12. Page 5, Section 2.4, the authors checked multicollinearity based on the values of SE for all predictors by citing Ref #23, which determined multicollinearity by the criteria of whether the magnitude of the SE for all predictors was > 0.5 by citing a study "Chan Y (2004) Biostatistics 202: logistic regression analysis. SingapMed J 45:149–153)." However, in the original work by Chan (2004), it was stated that measuring multicollinearity by inspecting the magnitude of the standard error (SE) of each variable is a simple but subjective technique because there is no fixed criterion on how small the SE should be but a matter of judgment. In the literature, a more widely used technique to diagnose multicollinearity is the variance inflation factor (VIF).

13. On page 6, 1st paragraph, the phrase "by conducting bivariate analysis" and the sentence "A Chi-square test was used to assess the association … with selected factors. " should be moved to the Methods section.

14. Page 6, 1st paragraph, in the sentence "… among the participants 54.4% had complete smoke-free policies at their home, 15.6% had partial … and 34% had no rules … (see Table 1)", there are two problems. First, the sum of 54.4%, 15.6%, and 34% equals 104.0%, which

is greater than 100%. There must be typos. Second, these numbers were not shown in Table 1. Similarly, in the next sentence, the reported percentages for smoke-free policy at workplace were not shown in Table 2.

15. Pages 6-7, the Results section needs to be substantially revised to improve the clarity. Some of the reported results (including Table 3) do not seem reasonable.
    a. For example, in the sentence, ".. elderly people were more likely to enjoy a completely smoke-free environment at their home than the younger counterparts.", who are the elderly people? Was the group aged 31-45 regarded as "elderly people"? What are the younger counterparts? Is this statement justified by the results in Table 3? I am puzzled why the AORs for the age groups 31-45, 46-60, and ≥60 were greater than 1 and highly significantly ($p < .001$) while the prevalence of smoke-free home rule was very similar across age groups (49.2% for the 15-30 group, 50.8% for the 31-45 group, 51.6% for the 46-60 group, and 50.6% for the ≥60 group. Was the sample weight used for the logistic regression analysis? I wonder whether the highly statistically significant results were due to the inflated sample size from using the sample weight. If this is the case, the models need to be re-estimated using the normalized weight.
    b. A sentence states that "Female counterparts had less likely tendency to live in a complete smoke-free atmosphere in their residence compared to the male counterparts." However, according to Table 1, the prevalence of smoke-free home rules was 50.7% for females and 50.0% for males. Their prevalence rates are quite close. It is peculiar why the AOR for female compared to male was less than 1 (AOR=0.569) and highly significant ($p < .001$).
    c. Another sentence states that "Participants who lived in rural areas were 1.211 times more likely to report that they worked in a completely smoke-free environment than their counterparts." However, according to Table 2, the prevalence of smoke-free workplace rule in rural areas was 52.1%, which is much lower than that in urban areas (69.4%). This illustrated that the reliability of Table 3 results is questionable.
    d. The sentence "Participants who lived in the South, East, and West region of India had the higher tendency to report that they worked in a completely smoke-free environment compared to other regions" is not justifiable by the Table 3 results.

16. On page 7, the 2nd paragraph, the phrase "According to a research study," should be deleted.

17. Pages 8-9, the Discussion section needs substantial revision by rigorously comparing the findings of this study with the literature, e.g,. how the findings are similar to or different from the previously published studies in the field, rather than repeating what has been presented in the Results section.

18. On page 8, the 2nd paragraph, the sentence "Married and separated people were more habituated to smoking in their workplace" is inconsistent with the results shown in Table 3.

19. On page 9, the 1st paragraph, the meaning of the 4 sentences ("The research paper demonstrated that …. It could be detrimental for children in a two-fold way. They could fall

victim …. But, if the children belonged to a highly educated and high income family, the exposure risk would decrease") is not clear. How are these sentences relevant to the findings of this study?

20. On page 9, the 2nd paragraph, the sentence "For instance, … such as section 4 (smoking in public places-59.28%), 6-a (implementation level-68.57%) and 6-b (implementation level-52.85%) were observed to be violated to some extent [37]." What does "implementation level" mean? How was it measured?

21. On Page 10, the authors cited a study conducted in the United States to explain the limitation of the GATS survey. This does not make sense.

22. Page 10, the last sentence — "… so that the projection of WHO about India being the only South Asian country to curb its smoking prevalence by 30% within 2025 will turn to be a success" is ambiguous. First, it needs to cite a reference about the WHO projected goal of smoking prevalence by 2014 for India. Second, it needs to explain what is the current smoking prevalence in India. This sentence seems to be contradictory to the information presented in the Introduction section (Page 3, lines 8-9): "Findings from the Global Adult Tobacco Survey 2 (GATS-2)-2017 indicate that 266.8 (28.6%) millions of all adults use tobacco in any form in India where 10.7% smoke tobacco". Wasn't India's cigarette smoking prevalence rate in 2016-2017 10.7%?

23. Tables 1-2 need to add a row to show results for all the persons in the final study sample.

24. Tables 3-4 need to show the total sample size for the analysis.

25. In the Supplementary Table A1, for the age variable, "Up to 60 years" should be corrected into "More than 60 years".

26. Inconsistent terms:
    a. Smoke-free policies vs. smoking policies. The term "smoking policies" used in several places (e.g., page 4, line 2) should be corrected into "smoke-free policies".
    b. Outcome variables & independent variables (Section 2.3, line 1 and line 13) vs. dependent variables & explanatory variables (page 5, Section 2.4, line 3).
    c. GATS2 vs. GATS 2

27. Inappropriate terms: "secondhand smoker's exposure" should be corrected into "secondhand smoke exposure".
