# Supplementary material for: Smoke-free status of homes and workplaces among Indian people: Evidence from Global Adult Tobacco SurveyData-2016/2017
Source: PLoS One. 2023 Feb 23;18(2):e0282138. doi: 10.1371/journal.pone.0282138 (PMC9949653; doi:10.1371/journal.pone.0282138)
Supplement: Attachment — Submitted filename: Rebuttal-Ver0.pdf [file pone.0282138.s007.pdf]

Authors' Response to Reviewers

Manuscript ID: PONE-D-20-41034R3

Title: Smoke-free policies in the home and in the workplace among Indian people: Evidence from Global Adult Tobacco Survey Data-2017

Dear Respected Editor-in-Chief,

First of all, we thank the reviewer and the editor for their useful suggestions for improving the manuscript. We have now revised the manuscript accordingly. Thank you once again and looking forward to the final acceptance in your esteemed journal.

Reviewer (R3):

The paper has been improved but important limitations remain:

(R3-1): We still miss a justification of the variables' choice. Mentioning that they have been used in the literature is not enough. We must understand which mechanisms may lead subgroups to adopt stricter norms or not. In the Discussion, no good explanations are presented for some groups adopting stricter norms (e.g., more educated or richer people).

Authors: Thank you for your valuable suggestions and giving us an opportunity to improve the revised manuscript. We try to add the justification of including various explanatory variables for this research work and modify the discussion section to include the possible reasons for adopting stricter norms among some specified groups (e.g., more educated or richer people).

(R3-2): Since this information is missing about potential causal mechanisms, the Discussion does not provide relevant insights for decision making.

Authors: Thanks again for your valuable suggestions. We have focused on this suggestion and rewritten some points in the discussion section to make the potential causal mechanisms clear. Thanks for understanding in advance.

(R3-2): The text needs a careful revision of English language.

Authors: Thank you for your comment. We have given a special attention to maintaining the standard English language for this article. Thanks for understanding in advance.

Dear Reviewer#3

Thank you very much for your encouragement, kind guidance, and support in helping the authors improve the manuscript.
We are truly grateful for your time and support.
Kind Regards,
The Authors of the manuscript.

Reviewer (R4):

(R4-1): I beg to differ with the other reviewer who suggested changing the term **"smoke-free policy" to "smoking norms"**. In my opinion, using the term **"smoke-free policy"** is more accurate.

Authors: We highly appreciate your valuable comments. We have changed the term "smoke-free policy" to "smoking norms" throughout the manuscript. Thanks for understanding in advance.

(R4-2): In Table 3, please place the number 1 to indicate the reference category in the AOR column instead of the p value column.

Authors: Thank you for your valuable suggestions. We regret making such a great mistake. Now, we have fixed it and updated the revised manuscript. Thanks again.

(R4-3): All other issues were addressed except for language, which still needs correcting. Example: In Discussion, Para 3-"Literatures are suggesting the fact than people engaged in other government/non government professional careers" needs to be rewritten. In its current form, the meaning of this sentence is unclear.

Authors: Thank you for your opinion. This point has been carefully addressed and updated in the revised manuscript. Moreover, we have given special attention to maintaining the standard English language for this article. Thanks again for your undderstadning in advance.

Dear Reviewer#4

Thank you very much for your encouragement, kind guidance, and support in helping the authors improve the manuscript.
We are truly grateful for your time and support.
Kind Regards,
The Authors of the manuscript.
