# Supplementary material for: Smoke-free status of homes and workplaces among Indian people: Evidence from Global Adult Tobacco SurveyData-2016/2017
Source: PLoS One. 2023 Feb 23;18(2):e0282138. doi: 10.1371/journal.pone.0282138 (PMC9949653; doi:10.1371/journal.pone.0282138)
Supplement: Attachment — Submitted filename: PONE-D-20-41043R4-Review.pdf [file pone.0282138.s008.pdf]

This study examines factors associated with smoke-free policies at home and in workplace using the 2017 Global Adult Tobacco Survey (GATS) in India. Additionally, the study examines the correlations between secondhand smoke exposure and smokefree policies. The variables were selected from the literature; therefore, the findings were mostly consistent with the existing literature. Nevertheless, this is the first kind of study in a country with the second largest of tobacco users in the world. The main results show that substantial number of people in the country are not covered by complete smokefree policies which is significant to inform policy initiatives. As such, it provides an added value to the literature. Further, the authors appear to be responsive to earlier reviews.

With the above said, here are few suggestions:

1) Consider creating a third paragraph for the introduction, starting with "Several studies have been conducted ...."

2) The study's aim is still not well-articulated in the paragraph #2.

3) In section 2.1, did you examine that the data you excluded were not significantly different from the analytic samples.

4) In section 2.4, add the name of the manufacturer and the City and country in a parenthesis after "STATA version 12 (...., ...)

5) In reporting the results (section 3.2), can you report OR, CI, and P-value?

6) Please note that this is a cross-sectional study; therefore, the results should not be interpreted as establishing a causation, instead of association

7) Key limitations of the study are missing, particularly biases such as recall and social desirability. The cross-sectional data by itself is a limitation

The minor issues involve attention to details ---

1) Place comma (,) before "respectively"

2) Delete "h" on the third line under section 4 Discussion

3) Consider removing grid on tables 2 and 3
