# Supplementary material for: Smoke-free status of homes and workplaces among Indian people: Evidence from Global Adult Tobacco SurveyData-2016/2017
Source: PLoS One. 2023 Feb 23;18(2):e0282138. doi: 10.1371/journal.pone.0282138 (PMC9949653; doi:10.1371/journal.pone.0282138)
Supplement: Attachment — Submitted filename: PONE-D-20-41034_Smoke.pdf [file pone.0282138.s009.pdf]

PLOS ONE

Smoke-free policies in the home and in the workplace among Indian people: Evidence from Global Adult Tobacco Survey Data-2016/2017

--Manuscript Draft--

**Manuscript Number:**	PONE-D-20-41034R4
**Article Type:**	Research Article
**Full Title:**	Smoke-free policies in the home and in the workplace among Indian people: Evidence from Global Adult Tobacco Survey Data-2016/2017
**Short Title:**	Smoke-free policies in the home and in the workplace among Indian people
**Corresponding Author:**	Sharlene Alauddin<br>Khulna University<br>Khulna, BANGLADESH
**Keywords:**	logistic models, prevalence, risk factors, tobacco smoking
**Abstract:**	Aims: This study aimed to determine the impact of correlates on tobacco control/smoke-free policies both at home and workplace in India. To assess the magnitude of the problem, the relationship between smoke-free policies and secondhand smoke (SHS) exposure was also explored.Methods: Data was extracted from the Global Adult Tobacco Survey Data (GATS)-2017. It was a household survey that included people aged 15 years or older and covered all the 30 states and 2 Union Territories (UTs) in India. A logistic regression model was used to determine the correlates of smoke-free policies in the home and workplace. Additionally, Pearson correlation was used to explore the relationship between smoke-free policies and the proportion of participants exposed to SHS both at home and workplace.Results: The overall prevalence of smoke-free policies in the home and workplace was 62.8% and 51.7%, respectively. Logistic regression results illustrated that tobacco smoking status, place of residence, region, education, occupation, wealth quintile, and knowledge status about children's illness were significantly associated with the respondent's intention to live in a completely smoke-free environment both at home and workplace in India. This study also focused on the fact that SHS exposure was significantly negatively associated with a smoke-free policy.Conclusion: This study will help the policymakers to promote efficient policies for improving smoke-free policies and ensure a better environment both at home and workplace in India.
**Order of Authors:**	Mohammad Ali
	Most. Farida Khatun
	Tasnuva Yasmin
	Ashis Talukder
	Md. Maniruzzaman
	Sharlene Alauddin
**Opposed Reviewers:**	
**Response to Reviewers:**	Authors' Response to Reviewers<br>Manuscript ID: PONE-D-20-41034R3<br>Title: Smoke-free policies in the home and in the workplace among Indian people: Evidence from Global Adult Tobacco Survey Data-2017<br>Dear Respected Editor-in-Chief,<br>First of all, we thank the reviewer and the editor for their useful suggestions for improving the manuscript. We have now revised the manuscript accordingly. Thank you once again and looking forward to the final acceptance in your esteemed journal.<br>Reviewer (R3):<br><br>The paper has been improved but important limitations remain:<br><br>(R3-1): We still miss a justification of the variables' choice. Mentioning that they have been used in the literature is not enough. We must understand which mechanisms may

Powered by Editorial Manager® and ProduXion Manager® from Aries Systems Corporation

	lead subgroups to adopt stricter norms or not. In the Discussion, no good explanations are presented for some groups adopting stricter norms (e.g., more educated or richer people).<br>Authors: Thank you for your valuable suggestions and giving us an opportunity to improve the revised manuscript. We try to add the justification of including various explanatory variables for this research work and modify the discussion section to include the possible reasons for adopting stricter norms among some specified groups (e.g., more educated or richer people).<br>(R3-2): Since this information is missing about potential causal mechanisms, the Discussion does not provide relevant insights for decision making.<br>Authors: Thanks again for your valuable suggestions. We have focused on this suggestion and rewritten some points in the discussion section to make the potential causal mechanisms clear. Thanks for understanding in advance.<br>(R3-2): The text needs a careful revision of English language.<br>Authors: Thank you for your comment. We have given a special attention to maintaining the standard English language for this article. Thanks for understanding in advance.<br>Dear Reviewer#3<br>Thank you very much for your encouragement, kind guidance, and support in helping the authors improve the manuscript.<br>We are truly grateful for your time and support.<br>Kind Regards,<br>The Authors of the manuscript.<br><br><br><br>Reviewer (R4):<br><br>(R4-1): I beg to differ with the other reviewer who suggested changing the term "smoke-free policy" to "smoking norms". In my opinion, using the term "smoke-free policy" is more accurate.<br>Authors: We highly appreciate your valuable comments. We have changed the term "smoke-free policy" to "smoking norms" throughout the manuscript. Thanks for understanding in advance.<br>(R4-2): In Table 3, please place the number 1 to indicate the reference category in the AOR column instead of the p-value column.<br>Authors: Thank you for your valuable suggestions. We regret making such a great mistake. Now, we have fixed it and updated the revised manuscript. Thanks again.<br>(R4-3): All other issues were addressed except for language, which still needs correcting. Example: In Discussion, Para 3-"Literatures are suggesting the fact than people engaged in other government/non government professional careers" needs to be rewritten. In its current form, the meaning of this sentence is unclear.<br>Authors: Thank you for your opinion. This point has been carefully addressed and updated in the revised manuscript. Moreover, we have given special attention to maintaining the standard English language for this article. Thanks again for your undderstadning in advance.<br><br>Dear Reviewer#4<br>Thank you very much for your encouragement, kind guidance, and support in helping the authors improve the manuscript.<br>We are truly grateful for your time and support.<br>Kind Regards,<br>The Authors of the manuscript.
**Additional Information:**	
**Question**	**Response**
**Financial Disclosure**<br><br>Enter a financial disclosure statement that describes the sources of funding for the work included in this submission. Review the submission guidelines for detailed	The authors received no specific funding for this work.

Powered by Editorial Manager® and ProduXion Manager® from Aries Systems Corporation

requirements. View published research articles from *PLOS ONE* for specific examples.<br><br>This statement is required for submission and **will appear in the published article** if the submission is accepted. Please make sure it is accurate.<br><br>**Unfunded studies**<br>Enter: *The author(s) received no specific funding for this work.*<br><br>**Funded studies**<br>Enter a statement with the following details:<br>• Initials of the authors who received each award<br>• Grant numbers awarded to each author<br>• The full name of each funder<br>• URL of each funder website<br>• Did the sponsors or funders play any role in the study design, data collection and analysis, decision to publish, or preparation of the manuscript?<br>• **NO** - Include this sentence at the end of your statement: *The funders had no role in study design, data collection and analysis, decision to publish, or preparation of the manuscript.*<br>• **YES** - Specify the role(s) played.<br><br>* typeset	
**Competing Interests**<br><br>Use the instructions below to enter a competing interest statement for this submission. On behalf of all authors, disclose any competing interests that could be perceived to bias this work—acknowledging all financial support and any other relevant financial or non-financial competing interests.<br><br>This statement **will appear in the published article** if the submission is accepted. Please make sure it is accurate. View published research articles from *PLOS ONE* for specific examples.	The authors have declared that no competing interests exist.

**NO authors have competing interests**<br><br>Enter: *The authors have declared that no competing interests exist.*<br><br>**Authors with competing interests**<br><br>Enter competing interest details beginning with this statement:<br><br>*I have read the journal's policy and the authors of this manuscript have the following competing interests: [insert competing interests here]*<br><br>* typeset	
**Ethics Statement**<br><br>Enter an ethics statement for this submission. This statement is required if the study involved:<br><br>• Human participants<br>• Human specimens or tissue<br>• Vertebrate animals or cephalopods<br>• Vertebrate embryos or tissues<br>• Field research<br><br>Write "N/A" if the submission does not require an ethics statement.<br><br>General guidance is provided below. Consult the submission guidelines for detailed instructions. **Make sure that all information entered here is included in the Methods section of the manuscript.**	This study considers a secondary dataset and the GATS2-2016/2017 survey was approved by<br>Ministry of Health and Family Welfare, Government of India. All data was completely anonymized, de-identified, and aggregated before access and analysis.

Powered by Editorial Manager® and ProduXion Manager® from Aries Systems Corporation

**Format for specific study types**<br><br>**Human Subject Research (involving human participants and/or tissue)**<br>• Give the name of the institutional review board or ethics committee that approved the study<br>• Include the approval number and/or a statement indicating approval of this research<br>• Indicate the form of consent obtained (written/oral) or the reason that consent was not obtained (e.g. the data were analyzed anonymously)<br><br>**Animal Research (involving vertebrate animals, embryos or tissues)**<br>• Provide the name of the Institutional Animal Care and Use Committee (IACUC) or other relevant ethics board that reviewed the study protocol, and indicate whether they approved this research or granted a formal waiver of ethical approval<br>• Include an approval number if one was obtained<br>• If the study involved *non-human primates*, add *additional details* about animal welfare and steps taken to ameliorate suffering<br>• If anesthesia, euthanasia, or any kind of animal sacrifice is part of the study, include briefly which substances and/or methods were applied<br><br>**Field Research**<br><br>Include the following details if this study involves the collection of plant, animal, or other materials from a natural setting:<br>• Field permit number<br>• Name of the institution or relevant body that granted permission	
**Data Availability**<br><br>Authors are required to make all data underlying the findings described fully available, without restriction, and from the time of publication. PLOS allows rare exceptions to address legal and ethical concerns. See the PLOS Data Policy and FAQ for detailed information.	Yes - all data are fully available without restriction

Powered by Editorial Manager® and ProduXion Manager® from Aries Systems Corporation

A Data Availability Statement describing where the data can be found is required at submission. Your answers to this question constitute the Data Availability Statement and **will be published in the article**, if accepted.<br><br>**Important:** Stating 'data available on request from the author' is not sufficient. If your data are only available upon request, select 'No' for the first question and explain your exceptional situation in the text box.<br><br>Do the authors confirm that all data underlying the findings described in their manuscript are fully available without restriction?	
**Describe where the data may be found in full sentences. If you are copying our sample text, replace any instances of XXX with the appropriate details.**<br><br>• If the data are **held or will be held in a public repository**, include URLs, accession numbers or DOIs. If this information will only be available after acceptance, indicate this by ticking the box below. For example: *All XXX files are available from the XXX database (accession number(s) XXX, XXX.).*<br>• If the data are all contained **within the manuscript and/or Supporting Information files**, enter the following: *All relevant data are within the manuscript and its Supporting Information files.*<br>• If neither of these applies but you are able to provide **details of access elsewhere**, with or without limitations, please do so. For example:<br><br>*Data cannot be shared publicly because of [XXX]. Data are available from the XXX Institutional Data Access / Ethics Committee (contact via XXX) for researchers who meet the criteria for access to confidential data.*<br><br>*The data underlying the results presented in the study are available from (include the name of the third party*	The data set used in this study will be available from the website of Centers for Disease Control and Prevention (CDCP). In order to gain access to the data files, you have to complete the form. The data set is available from the following link: nccd.cdc.gov/GTSSDataSurveyResources/Ancillary/DataReports.aspx?CAID=2.

Powered by Editorial Manager® and ProduXion Manager® from Aries Systems Corporation

and contact information or URL).<br>• This text is appropriate if the data are owned by a third party and authors do not have permission to share the data.<br><br>* typeset	
Additional data availability information:	Tick here if the URLs/accession numbers/DOIs will be available only after acceptance of the manuscript for publication so that we can ensure their inclusion before publication.

Khulna University
Khulna-9208, Bangladesh
Website: www.ku.ac.bd

Date: July 20, 2022

To
Editor-in-Chief, PloS One
Dear Respected Editor:

It is an honor to submit our new manuscript entitled "***Smoke-free policies in the home and in the workplace among Indian people: Evidence from Global Adult Tobacco Survey Data-2016/2017***" to your PloS One journal. I confirm that this work is original and has not been published elsewhere nor it is currently under consideration for publication elsewhere.

We have taken the rebuttal from both the reviewers/editors and converted their comments into serial numbers. We have addressed each and every comment very comprehensively and we are very confident that they will like it. We want to thank you, the Editor and both the reviewers for giving us the chance to update the manuscript. We are eagerly waiting the positive response.

We look forward to your blessings and positive critique.

Best Regards

Sharlene Alauddin
Assistant Professor
Statistics Discipline
Khulna University
Khulna-9208, Bangladesh
sharlene.kst@gmail.com

Manuscript

Click here to access/download;Manuscript;Revised-Manuscript with unhighlighted-Ver2.docx

Title Page

Smoke-free policies in the home and in the workplace among Indian people: Evidence from Global Adult Tobacco Survey Data-2016/2017

Mohammad Ali[1], Most. Farida Khatun[2], Tasnuva Yasmin[3], Ashis Talukder[1], Md. Maniruzzaman[1], Sharlene Alauddin[1*]

[1]Statistics Discipline, Khulna University, Khulna-9208, Bangladesh
[2]Pharmacy Discipline, Khulna University, Khulna-9208, Bangladesh
[3]Development Studies Discipline, Khulna University, Khulna-9208, Bangladesh

Contributors: All authors equal contribution

***Corresponding author**
Sharlene Alauddin
Assistant Professor
Statistics Discipline
Khulna University
Khulna-9208, Bangladesh
sharlene.kst@gmail.com

Smoke-free policies in the home and in the workplace among Indian people: Evidence from Global Adult Tobacco Survey Data-2016/2017

Abstract

Aims: This study aimed to determine the impact of correlates on tobacco control/smoke-free policies both at home and workplace in India. To assess the magnitude of the problem, the relationship between smoke-free policies and secondhand smoke (SHS) exposure was also explored.

Methods: Data was extracted from the Global Adult Tobacco Survey Data (GATS)-2017. It was a household survey that included people aged 15 years or older and covered all the 30 states and 2 Union Territories (UTs) in India. A logistic regression model was used to determine the correlates of smoke-free policies in the home and workplace. Additionally, Pearson correlation was used to explore the relationship between smoke-free policies and the proportion of participants exposed to SHS both at home and workplace.

Results: The overall prevalence of smoke-free policies in the home and workplace was 62.8% and 51.7%, respectively. Logistic regression results illustrated that tobacco smoking status, place of residence, region, education, occupation, wealth quintile, and knowledge status about children's illness were significantly associated with the respondent's intention to live in a completely smoke-free environment both at home and workplace in India. This study also focused on the fact that SHS exposure was significantly negatively associated with a smoke-free policy.

Conclusion: This study will help the policymakers to promote efficient policies for improving smoke-free policies and ensure a better environment both at home and workplace in India.

1 Introduction

Tobacco use is a major risk factor for non-communicable diseases and one of the top preventable causes of worldwide premature deaths [1]. All forms of tobacco products, whether they are smoked, inhaled, chewed, or sucked, cause serious damage to health [2]. Tobacco kills more than 8 million people each year globally, and among them, 7 million people are died due to direct tobacco use, while around 1.2 million deaths occur due to secondhand smoke (SHS) [3]. India is the second-largest consumer of tobacco after China in the world and one-third of its population uses tobacco [4, 5]. According to the report of the Global Adult Tobacco Survey 2 (GATS 2)-2017, 266.8 (28.6%) million adults use tobacco in any form in India, whereas 10.7% of people use smoke tobacco and 21.4% of people use smokeless tobacco [6]. The prevalence of male and female tobacco consumers is 42.4% and 14.2% [6]. According to the GATS 2 (2016/2017) report, among the total of SHS, 38.7% (38.1% male vs. 39.3% female) of people takes smoke at home, 30.2% (32.7% male vs. 17.9% female) at workplace and 5.3% (8.1% male vs. 2.4% female) at government office, 5.6% (6.8% male vs. 4.4% female) at health care facilities, 7.4% (13.0% male vs. 1.6% female) at restaurants [6]. Asian women have a lower tendency to smoke while most of them and their children are mainly victims of SHS and both suffer from various smoking-induced harms [7]. According to previous studies, about one-third of non-smokers are adults and 40% of children are exposed to SHS [7, 8]. Several studies showed that SHS increased the risks of lung cancer, stroke, bronchitis, childhood diseases, and heart disease [9-11].

To protect non-smokers from SHS, legislation was enacted by the parliament of India in 2003 entitled "Cigarettes and Other Tobacco Products Act 2003" (COTPA 2003) which prohibits smoking in public and work premises [12]. The provisions under this law were notified to the public from 2004 to 2006. The Government faced many legal challenges from the tobacco industry during this period. However, in 2008, after a long legal fight and intervention of civil society, a remarkable effect of these rules was found both in public and workplaces [38]. Additionally, to fulfill the commitments under the WHO Framework Convention on Tobacco Control (FCTC) and to facilitate effective implementation of COTPA 2003, the Ministry of Health and Family Welfare, Government of India, launched the National Tobacco Control Program in 2007-2008 [12]. Although there is progress in smoke-free policies both in indoor public/workplaces and public transportation, the policies are still not comprehensive [6, 13]. All of these rules come to the public as a Government or administrative order and there is a lack of awareness in following any legal instruments. Without strong enforcement

and clear guidelines for the citizens about their right to smoke, the implementation of these policies will remain to be largely ineffective [39]. Several studies have been conducted on tobacco smoking in India [14-17]. However, most of these previous studies have only focused on the prevalence and predictors of tobacco use [14-17]. Another study investigated SHS exposure at home and in public places among smokers and non-smokers [18]. A study also observed that a smoke-free workplace was significantly associated with SHS exposure at home [19, 20]. Previous research showed that the presence of policies that restrict smoking in the workplace and home may varied by geographical region, occupation, and socioeconomic status [21]. Noticeably, no previous study focused on the risk factors affecting the functionality of smoke-free policies both at home and workplace in India. This is the first study in India, to determine the influential correlates of smoke-free policies both at home and workplace respectively. Besides, this study explores the relationship between smoke-free policies and SHS exposure by measuring the correlation between the state-level proportion of respondents at both premises.

2 Materials and methods

2.1 Data

The data was extracted from the Global Adult Tobacco Survey 2 (GATS 2), which was conducted in India during August 2016-February 2017. A detailed description of the survey objectives, design, methods, and questionnaires can be found elsewhere [6]. In brief, GATS 2 is a global standard for systematically monitoring the status of using tobacco among adults and tracking key control policies. [illegible]s study conducted a household survey of people aged 15 years or older, covering all the 30 states and 2 Union Territories (UTs) in India. A multistage stratified cluster sampling design was applied to collect the sample from each state as well as particularly from urban and rural areas. In GATS 2, a total of **74,037** individual interviews were completed with an overall response rate of 92.9%. The analysis of the smoke-free policies at the workplace was restricted to the GATS 2 respondents who were working indoors or both indoors and outdoors. After removing the respondents with missing values, the final analysis of the smoke-free policies was conducted with ~~**71,046**~~ respondents from home and **15,254** from workplaces.

2.2 Ethics approval and consent to participate

This study utilized a secondary dataset that was publicly available and the GATS 2-2016/2017 survey was approved by the Ministry of Health and Family Welfare, Government of India. All data was completely anonymized, de-identified, and aggregated before access and analysis.

2.3 Variables of the study

Smoke-free policies both at home and workplace were considered as the dependent variables for this study. It is classified into binary categories in the following ways: the respondent was classified as living in a completely smoke-free home if he/she answered "smoking is never allowed inside your home" to the question "Which of the following best describes the rules about smoking inside your home?" If the answer was "smoking is allowed inside your home," "smoking is generally not allowed but exceptions," or "there are no rules about smoking at your home" he/she was considered as not living in a completely smoke-free home. Similarly, the respondent was classified as working in a completely smoke-free environment if he/she answered "not allowed in any indoor areas" to the question "Which of the following best describes the indoor smoking policy where you work?" If the answer was "smoking is allowed anywhere", "allowed only in some indoor areas", or "there is no policy", he/she was considered as not working in a completely smoke-free environment. The descriptions of the independent variables along with their categorizations are presented in Table 1.

In this study, to construct the wealth quintile variable, the following indicators were used: Whether the household or person living in the household enjoys the facilities of i) electricity, ii) electric fan, iii) air conditioner, iv) refrigerator, v) washing machine, vi) radio, vii) television, viii) computer/laptop, ix) internet connection, x) moped/scooter xi) car, xii) flush toilet, xiii) fixed telephone, xiv) cell telephone. Principal component analysis (PCA) ~~has~~ been conducted to create this variable and it was divided into five categories (poorest, poorer, medium, richer, and richest) based on the quintiles of the first PCA scores. The estimated proportion of participants living in smoke-free homes and workplaces was collected and the state-level correlation between the proportion of smoke-free policies and SHS exposure at both home and workplace was also explored. GATS 2 asked the following questions to measure the SHS exposure at home and workplace as follows: (i) How often does anyone smoke inside your home? Would you say daily, weekly, monthly, less than monthly, or never?' Respondents who indicated 'daily', 'weekly', or 'monthly' were classified as exposed to SHS in the home; (ii) For the question, during the past 30 days, did anyone smoke in indoor areas where you work?', respondents who answered 'yes' were classified as exposed to SHS in the workplace. Reviewing some pre-existing literatures, other relevant explanatory variables (given in Table 1) were selected [5,9,7,15,17,18].

2.4 Statistical analysis

Descriptive analysis was performed and expressed as frequency (percentage) of smoke-free policies both in home and workplaces. A Chi-square test was conducted to assess the bivariate association between the dependent and independent variables. For multivariate analysis, a logistic regression model was used to estimate the adjusted odds ratio (AOR) of the selected independent variables along with their correspondence p-values and 95% confidence interval. We determined the most significant variables of smoke-free policies both at home and workplace using p-value (<0.001). In this study, the multicollinearity among different independent variables was checked by variance inflation factor (VIF) values. The VIF values less than 10 indicated, no existence of significant multicollinearity between the independent variables [22]. The entire data analysis, data cleaning, and management were carried out using STATA version 12. Sampling weights were applied and the weighted estimates were calculated to consider the complex study design. Clustering and stratification were also counted with the svyset command in STATA. The following variables were used to apply clustering and stratification: gatscluster, gatsstrata and gatsweight. A Pearson correlation test was carried out to explore the relationship between smoke-free policies and the proportion of SHS exposure at both home and workplace.

3 Results

3.1 Prevalence of complete smoke-free ~~policies~~ in home and workplace

The overall prevalence of complete smoke-free policies at home and workplace were 62.8% and 51.7% (See Table 2). About 25% and 48.5% of tobacco smokers follow smoke-free policies in their home and workplace. The major proportion of participants who lived in rural areas was reported to follow smoke-free policies both in their home and workplace. The Chi-square results revealed that all factors except respondents' age and gender were significantly associated with the home-based smoke-free policies, whereas all other factors except age were significantly associated with smoke-free policies at the workplace (p<0.001) (See Table 2).

3.2 ~~Logistic regression analysis~~

Only significant factors were considered to perform in the logistic regression model for the home and workplace. Since the VIF of all selected independent variables was less than 10, it indicated that there were no multicollinearity problems among the selected independent variables (~~See~~ Table 3). We found that non-smokers were more likely to live and work in a completely smoke-free environment in their home [AOR=3.285] and workplace [AOR=1.627] compared to smokers (~~See~~ Table 3). We also

found that female participants were more likely to enjoy the smoke-free environment at their workplace. Other religious people were 1.939 times more likely to follow complete smoke-free policy at home compared to Hindu religious people. The participants who lived in rural areas were 1.376 and 1.245 times more likely to live in a completely smoke-free environment at home and workplace respectively compared to urban counterparts. The participants who lived in the South region of India had the highest tendency to continue living and working with complete smoke-free policy compared to other regions. Highly educated participants had a higher tendency to maintain a completely smoke-free environment at their home [AOR=1.879] and workplace [AOR=2.314]. Students and other participants had the lowest tendencies to live in a completely smoke-free environment at their home, while daily wise labor, self-employed, and students were less likely to report that their workplace was smoke-free compared to government/non-government employees. The participants who belong to rich families were 1.176 times more likely to live complete smoke-free in their homes. Whereas, the rich and richest participants had a higher tendency to report that their workplaces were completely smoke-free compared to the poorest participants. The participants who were knowledgeable and careful about children's illnesses from other people's smoke had more tendencies to maintain and ensure a complete smoke-free policy in their homes compared to their counterparts.

3.3 Correlation analysis

We performed a correlation analysis to assess the state-level relationship between a smoke-free policy with SHS exposure at home and workplace. It was observed that the smoke-free policies of both home and workplace was significantly negatively associated with ~~SHS exposure~~ (See Table 4).

4 Discussion

The results of the research paper demonstrated that non-smoker participants were more likely to live in both completely smoke-free in their home and workplace. Besides, elderly people composed of a varying age group from 45 onwards 65 and above are mostly tended to stay smoke-free in their home h[18-20]. Similarly, people of an elder age supposedly maintained the highest rate of smoke-free policies [19-21]. Based on gender, women's likelihood to live in a smoke-free environment at workplace was higher than men [18, 19, 21, 22]. Some literature opposed this statement revealing that women's possibility to live in a completely smoke-free environment was less than their male counterparts [20, 23-25]. This phenomenon pointed to the fact that in the societal context, mostly men were associated with smoking and women were not even in a position to initiate a protest against this behavior [23, 26]. Additionally, female people emphasized more on a smoke-free zone in their workplace more than their opposite gender

[19-22]. Married people were less tending to enjoy a complete non-smoking work environment as well as their home than their single counterparts [22]. Married and separated people were more habituated to smoking in their workplace [19]. Contrarily, separated persons were expected to enjoy a more smoke-free environment in their residence.

Religious factors were found to be reflected in ensuring smoke-free policies. Muslims and Christians were less likely to maintain a smoke-free environment in their home. This might be because they are not enough concerned about their family health issues [27]. On the other hand, Muslim respondents were more likely to report that their workplaces were completely smoke-free than their counterparts. Religious services both on social and personal levels urged people to not be accustomed to smoking or a least consume fewer cigarettes than non-religious people [28]. People living in rural areas were more prone to live in a completely smoke-free environment both at home and workplace compared to the urban population [18, 29, 30] which scenario was true in the context of some countries [18,19]. Existing literature agreed with the current study in the sense that on a regional basis, south Indian people possessed the highest tendency to live in a completely smoke-free environment at home and workplace [18, 20]. People from central, east, and west were also expected to enjoy a smoke-free environment in their homes. Alongside the people of the east, the west had the opportunity to work in a smoke-free place/zone.

Literature supported the finding of this study that higher educated people were more likely to live in a smoke-free home environment [18, 19, 21-24, 30]. But there was contradictory information regarding higher educated people's SHS exposure in their workplace as well as at home. In one study, it was pointed out that the possibility of the higher educated people being in a workplace exposed to smoking was also greater than relatively lower educated people [19, 20]. Previous Literature were suggested the fact that people of other occupations tended to experience a lower risk to have an unspoiled smoking contaminated job place as well as a home environment than people engaged in other government / non-government professional careers [20, 30].

People who possessed a relatively high level of income were more prone to have a complete smoke-free household and workplace than the poorest ones [21, 24, 31]. Therefore, socioeconomic conditions contributed to enhancing the cautiousness among the people. The research paper demonstrated that if a parent was a regular smoker both at home and workplace then the chance of children being exposed to SHS would increase [20]. It could be detrimental to children in a two-fold way. They could fall victim to various illnesses and along with that, the risk of smoking could also increase among

teenagers if their parents were accustomed to being aware of it [31, 32]. But, if the children belonged to a highly educated and high-income family, the exposure risk would decrease [21]. Parents having this knowledge intend to enjoy a smoke-free policy both at their home and workplace. This study also observed that smoke-free policies in the home and the workplace were significantly positively associated with each other whereas both policies were significantly negatively associated with different settings of secondhand smokers. It was indicated that smoke-free policies played a vital role to control secondhand smoking-induced problems. However, enacting a policy does not necessarily mean that it would be completely implemented throughout the country and this is true for the COTPA also. For instance, in 2020, a study was conducted in Delhi assessing the implementation level of COTPA 2003 in 376 public places in the city. Various sections of the act such as section 4 (smoking in public places-59.28%), 6-a (implementation level-68.57%), and 6-b (implementation level-52.85%) were observed to be violated to some extent [34]. The case of Bengaluru was also similar in the sense that this city also witnessed the violation of various sections of the act. The absence of a 'non-smoking sign' was 89.7% and 76% of the educational establishments had an abundance of shops selling nicotine products closer to their radius [35]. Besides the cities, the rural and tribal populations also suffer from the problem of the implementation of smoke-free policies. India possesses a tribal population of more than 100 million who also belong to the highest level of smoking but because of location and associated resource deprivation, implementation of policies is poor among them [36].

However, it is prevalent from the previous discussion that some sub-groups of the respondents are more exposed to secondhand smoking than their counterparts. While looking for a causal relationship, literatures point out towards the fact that if workplaces put an embargo upon smoking, it highly decreases the rate of smoking and as a result it decreases the exposure to SHS in the workplace also [37]. Furthermore, if an individual is forced to be refrained from smoking in the workplace, it would ultimately lead to quit smoking in the home also. Besides workplace embargo upon smoking, studies also show that banning any kind of advertisement of cigarettes in the media or inclusion of smoking in movie scenes will greatly curb people's desire to smoking [38]. Now, as higher educated people and people with higher income level are generally more exposed to such workplace ban as well as awareness raising campaigns against SHS, their rate of engaging in smoking and exposure to SHS is relatively lower.

Though the existing literature showed discrepancies between the enacting and implementation of a policy, it was also mentioned that a collaborative emphasis on the implementation of these policies from the institutions, policymakers, academics, media, and civil society would increase the level of

implementation [35, 39]. Literature also mentioned that studying the patterns of smoking from different angles will help further improve preventive tools for smoke-free policies [36]. The current study also made a similar observation that smoke-free policies and SHS exposure were negatively correlated. It is indicated that an improvement in the smoke-free policies could be crucial in reducing the proportion of SHSr at every level.

4.1 Strength and limitations

This study was conducted based on a large national representative GATS survey. This survey was considered a global standard for monitoring the impact of key tobacco control policies. Earlier studies had analyzed comprehensive information on tobacco smoke-free policies in both home and workplace among different countries, but none of them investigated the situation in India despite being at a state of high risk for tobacco. Therefore, this research work is expected to assist the policymakers in imply more efficient policies for tobacco control in India. In this study, the most significant variable, like the wealth index was indirectly formed. Also, the trend of smoke-free policies at home, as well as workplace, wasn't explored. However, we didn't adjust some other possible confounding factors which could be explored further.

5 Conclusion

This study found that 62.8% of respondents were living under a complete smoke-free policy at home, and 51.7% at the workplace. This study also found that tobacco smoking status, place of residence, region, education, occupation, wealth quintile, and knowledge status about the children's illness from other people's smoking were significantly associated with the complete smoke-free policies in home, while all these significant factors along with gender, were also associated with the complete smoke-free policies for the workplace in India. Smoke-free policies in both home and workplace were significantly negatively associated with SHS exposure. Indian authorities should emphasize these findings as a supporting document while implementing tobacco control policies.

Acknowledgements

The authors would like to acknowledge the authority of the GATS 2-2016/2017.

Availability of data and materials

The data set used in this study will be available from the website of the Centers for Disease Control and Prevention (CDCP). To gain access to the data files, you have to complete the form. The data set is available from the following link.

nccd.cdc.gov/GTSSDataSurveyResources/Ancillary/DataReports.aspx?CAID=2

Competing interests

None declared

Funding

The authors did not receive any funds for this study.

References

1. Alwan A. Global status report on noncommunicable diseases 2010. 2011: World Health Organization.
2. Bauer UE, Briss PA, Goodman RA, Bowman BA. Prevention of chronic disease in the 21st century: elimination of the leading preventable causes of premature death and disability in the USA. The Lancet. 2014; 384(9937):45-52.
3. World Health Organization. WHO report on the global tobacco epidemic, 2019: offer help to quit tobacco use: executive summary, in WHO report on the global tobacco epidemic, 2019: offer help to quit tobacco use: executive summary. 2019.
4. Ministry of Health and Family Welfare, G.o.I., Global Adult Tobacco Survey India, 2009-2010. New Delhi, India. 2010.
5. Kaur J, Jain DC. Tobacco control policies in India: implementation and challenges. Indian journal of public health. 2011;55(3):220.
6. Tata Institute of Social Sciences (TISS), M.M.o.H.a.F.W., Government of India., Global Adult Tobacco Survey GATS 2 India 2016-17. 2017.
7. Öberg M, Jaakkola MS, Woodward A, Peruga A, Prüss-Ustün A. Worldwide burden of disease from exposure to second-hand smoke: a retrospective analysis of data from 192 countries. The lancet. 2011;377(9760):139-46.
8. Eriksen MJ, Mackay, H Ross. The tobacco atlas. 2013: American Cancer Society.
9. Oberg M. Second hand smoke: Assessing the burden of disease at national and local levels. 2010
10. Samet JM, Avila-Tang E, Boffetta P, Hannan LM, Olivo-Marston S, Thun MJ, et al. Lung cancer in never smokers: clinical epidemiology and environmental risk factors. Clinical Cancer Research. 2009;15(18):5626-45.
11. Fischer F, A Kraemer. Meta-analysis of the association between second-hand smoke exposure and ischaemic heart diseases, COPD and stroke. BMC Public Health, 2015; 15(1): 1202.
12. Schwartz RL, Wipfli HL, Samet JM. World No Tobacco Day 2011: India's progress in implementing the framework convention on tobacco control. The Indian journal of medical research. 2011;133(5):455.
13. Mistry R, Dasika A. Antenatal tobacco use and secondhand smoke exposure in the home in India. Nicotine and Tobacco Research. 2018;20(2):258-61.

14. Mohan P, Lando HA, Panneer S. Assessment of tobacco consumption and control in India. Indian Journal of Clinical Medicine. 2018;9:1179916118759289.

15. Guindon GE, Nandi A, Chaloupka IV FJ, Jha P. Socioeconomic differences in the impact of smoking tobacco and alcohol prices on smoking in India. National Bureau of Economic Research. 2011.

16. Prasad R, Suryakant RG, Singhal S, Dawar R, Agarwal GG. A case-control study of tobacco smoking and tuberculosis in India. Annals of thoracic medicine. 2009;4(4):208.

17. Mistry R, Pednekar MS, Gupta PC, Raghunathan TE, Appikatla S, Puntambekar N, et al. Longitudinal study of adolescent tobacco use and tobacco control policies in India. BMC public health. 2018;18(1):815.

18. Tripathy, J.P. Secondhand smoke exposure at home and public places among smokers and non-smokers in India: findings from the Global Adult Tobacco Survey 2016–17. *Environ Sci Pollut Res* 27, 6033–6041 (2020).

19. Tripathy, J. P. (2020). Smoke-free workplaces are associated with smoke-free homes in India: evidence for action. *Environmental Science and Pollution Research*, *27*(33), 41405-41414.

20. Lee JT, Agrawal S, Basu S, Glantz SA, Millett C. Association between smoke-free workplace and second-hand smoke exposure at home in India. Tobacco control. 2014

;23(4):308-12.

21. Berg CJ, Daley CM, Nazir N, Cully A, Pacheco CM, Buchanan T, et al. Smoke-free policies in the workplace and in the home among American Indians. Journal of health disparities research and practice. 2012;5(2):7.

22. Tripathy JP, Verma M. Impact of Health Warning Labels on Cigarette Packs in India: Findings from the Global Adult Tobacco Survey 2016–17. Behavioral Medicine. 2020; 20:1-0.

23. Ayo-Yusuf OA, Olufajo O, Agaku IT. Exposure to secondhand smoke and voluntary adoption of smoke-free home and car rules among non-smoking South African adults. BMC public health. 2014;14(1):580.

24. Max W, Sung HY, Shi Y. Exposure to secondhand smoke at home and at work in California. Public health reports. 2012;127(1):81-8.

25. Singh A, Sahoo N. Urban–rural differentials in the factors associated with exposure to second-hand smoke in India. BMJ open. 2013;3(11).

26. Nazar GP, Lee JT, Glantz SA, Arora M, Pearce N, Millett C. Association between being employed in a smoke-free workplace and living in a smoke-free home: evidence from 15 low and middle income countries. Preventive Medicine. 2014; 59:47-53.

27. Bonevski B, Paul C, Jones A, Bisquera A, Regan T. Smoky homes: Gender, socioeconomic and housing disparities in second hand tobacco smoke (SHS) exposure in a large population- based Australian cohort. Preventive medicine. 2014; 60: 95-101..

28. Awawdi K, Steiner H, Green MS, Zelber-Sagi S. Association between second-hand smoking and acute coronary heart disease among Arab women with multiple risk factors. The European Journal of Public Health. 2016;26(1):141-5.

29. Olasky SJ, Levy D, Moran A. Secondhand smoke and CVD in low-and middle-income countries: A case for action. Global heart. 2012;7(2):151-60.

30. Yong HH, Hamann SL, Borland R, Fong GT, Omar M. Adult smokers' perception of the role of religion and religious leadership on smoking and association with quitting: A comparison between Thai Buddhists and Malaysian Muslims. Social science & medicine. 2009;69(7):1025-31.

31. Koenig HG, George LK, Cohen HJ, Hays JC, Larson DB, Blazer DG. The relationship between religious activities and cigarette smoking in older adults. The Journals of Gerontology Series A: Biological Sciences and Medical Sciences. 1998;53(6):M426-34.

32. Kaleta D, Polanska K, Usidame B. Smoke-free workplaces are associated with protection from second-hand smoke at homes in nigeria: evidence for population-level decisions. BioMed research international. 2015.

33. Kabir Z, Alpert HR, Goodman PG, Haw S, Behm I, Connolly GN, et al. Effect of smoke-free home and workplace policies on second-hand smoke exposure levels in children: an evidence summary. Pediatric Health. 2010;4(4):391-403.

34. Ali, I., Patthi, B., Singla, A., Dhama, K., Muchhal, M., Rajeev, A., ...& Khan, A. (2020). Assessment of implementation and compliance of (COTPA) Cigarette and Other Tobacco Products Act (2003) in open places of Delhi. *Journal of Family Medicine and Primary Care*, 9(6), 3094.

35. Habbu, S. G., &Krishnappa, P. (2015). Assessment of implementation of COTPA-2003 in Bengaluru city, India: A cross-sectional study. *Journal of Indian Association of Public Health Dentistry*, 13(4), 444.

36. Mohan, P., Lando, H. A., &Panneer, S. (2018). Assessment of tobacco consumption and control in India. *Indian Journal of Clinical Medicine*, 9, 1179916118759289.

37. Farrelly, M. C., Evans, W. N., & Sfekas, A. E. (1999). The impact of workplace smoking bans: results from a national survey. Tobacco Control, 8(3), 272-277.

38. Yang, T., Rockett, I. R., Li, M., Hu, X., & Gu, Y. (2012). Tobacco advertising, environmental smoking bans, and smoking in Chinese urban areas. Drug and alcohol dependence, 124(1-2), 121-127.

39. Panda, B., Rout, A., Pati, S., Chauhan, A. S., Tripathy, A., Shrivastava, R., &Bassi, A. (2012). Tobacco control law enforcement and compliance in Odisha, India-Implications for tobacco control policy and practice.

Table 1. Descriptions of the independent variables along with their categorizations

Factors	GATS asked the following questions	Categorizations
Tobacco smoking status	Does this person currently smoke tobacco, including bidis, cigarettes, hukkah, cigars, and pipes?	1= Smoker, 2= Non-smoker
Age (in years)	What is the age of the selected person?	1=15-30, 2= 31-45, 3= 46-60, 4=More than 60
Gender	Record the gender of the selected person	1= Male, 2= Female
Marital status	What is your marital status?	1= Single, 2= Married, 3= Separated/others
Religion	What is your religion?	1=Hindu,2=Muslim,3=Christian, 4=Others
Place of residence	Residence status	1= Urban, 2=Rural
Region	National geographical regions	1=North, 2= Central, 3= East, 4= North-east, 5= West, 6= South
Education	What is the highest level of education you completed?	1=No education,2=Up to primary, 3=Up to secondary, 4= More than secondary
Occupation	Which of the following best describes your main work status over the past 12 months?	1=Govt./Non-Govt. employee,2=Daily wise/causal laborer, 3= Self-employed, 4=Homemaker, 5= Student, 6= Others
Wealth quintile	How would you describe the status of your Wealth quintile?	1= Poorest, 2= Poorer, 3= Middle, 4= Richer, 5= Richest
Knowledge status about children' illness	Based on what you know or believe, does smoking tobacco cause serious illness among the children?	1= No, 2= Yes

Table 2. Correlates of smoke-free policies in the home and the workplace in India

Characteristics	Smoke-free policies in					
	Home (n= 15,254)			Workplace (n= 71,046)		
	Yes %	No, %	p-value	Yes, %	No, %	p-value[1]
Overall	62.8	37.2		51.7	48.3	
**Tobacco smoking status**			<0.001			<0.001
Smoker	28.5	71.5		48.5	51.5	
Non-smoker	56.1	43.9		66.9	33.1	
**Age (in year)**			0.944			0.676
**15-30**	51.8	48.2		63.6	36.4	
31-45	51.8	48.2		61.8	38.2	
46-60	51.3	48.7		62.7	37.3	
More than 60	51.5	48.5		63.2	36.8	
**Gender**			0.205			<0.001
Male	52.2	47.8		61.6	38.4	
Female	51.1	48.9		68.9	31.1	
**Religion**			<0.001			0.0086
Hindhu	51.9	48.1		63.5	36.5	
Muslim	45.4	54.6		57.1	42.9	
Christian	65.4	34.6		64.6	35.4	
Others	35.0	65.0		68.5	31.5	
**Marital status**			<0.001			<0.001
Single	56.0	44.0		69.0	31.0	
Married	50.2	49.8		60.9	39.1	
Separated/others	52.6	47.4		58.9	41.1	
**Place of residence**			<0.001			<0.001
Urban	46.1	53.9		56.0	44.0	
Rural	62.1	37.9		70.8	29.2	
**Region**			<0.001			<0.001
North	56.3	53.7		62.3	37.7	
Central	38.3	61.7		52.7	47.3	
East	43.9	56.1		58.1	41.9	
North-east	43.3	56.7		55.9	44.1	
West	56.6	43.4		74.7	25.3	
South	77.9	22.1		72.2	27.8	
**Education**			<0.001			<0.001
No education	41.5	58.5		41.5	58.5	
Up to primary	46.4	53.6		50.8	49.2	
Up to secondary	53.6	46.4		64.8	35.2	
More than secondary	65.1	34.9		76.3	23.7	
**Occupation**			<0.001			<0.001
Govt./ Non-Govt. employee	60.7	39.3		75.5	24.5	
Daily wage/ causal labourer	46.8	53.2		46.6	53.4	
Self employed	49.1	50.9		58.2	41.8	
Homemaker	59.6	40.4		84.0	16.0	
Student	50.5	49.5		52.8	47.2	
Others	50.1	49.9		58.0	42.0	

**Wealth quintile**			<0.001			<0.001
Q_1 (poorest)	39.1	60.9		46.1	53.9	
Q_2	48.7	51.3		56.7	43.3	
Q_3	54.5	45.5		62.1	37.9	
Q_4	62.8	37.2		74.2	25.8	
Q_5 (richest)	59.6	40.4		72.5	27.5	
**Knowledge status about children' illness**			<0.001			0.484
Yes	52.1	47.9		62.9	37.1	
No	43.4	56.6		60.0	40.0	

Q1: Poorest; Q2: Poorer; Q3: Middle; Q4: Richer; Q5: Richest.

Table 3. Multivariable logistic regression results

Variable		Smoke-free policies in					
		Home			Workplace		
		p-value	AOR (95% CI of AOR)	VIF	p-value	AOR (95% CI of AOR)	VIF
**Tobacco smoking status**	Smoker		1.00	1.05		1.00	1.11
	Non-smoker	<0.001	3.29 (3.00,3.59)		**<0.001**	1.63 (1.40, 1.89)	
**Gender**	Male		-			1	1.11
	Female		-		**0.048**	1.23 (1.00, 1.51)	
**Marital status**	Single		1.00	1.13		1.00	1.06
	Married	0.420	0.96 (0.87, 1.06)		0.604	0.95 (0.79, 1.14)	
	Separated/others	0.274	1.08 (0.94, 1.24)		0.934	0.98 (0.68, 1.43)	
**Religion**	Hindhu		1.00	1.01		1.00	1.01
	Muslim	0.074	0.89 (0.77, 1.01)		0.510	1.07 (0.88, 1.31)	
	Christian	0.901	0.99 (0.81, 1.21)		0.084	0.71 (0.48, 1.05)	
	Others	<0.001	1.94 (1.65, 2.28)		0.589	1.09 (0.78, 1.55)	
**Place of residence**	Urban		1.00	1.21		1.00	1.19
	Rural	<0.001	1.38 (1.25, 1.51)		0.015	1.25 (1.04, 1.48)	
**Region**	North		1.00	1.01		1.00	1.01
	Central	0.434	0.95 (0.84, 1.08)		0.170	0.87 (0.71, 1.06)	
	East	<0.001	1.25 (1.11, 1.41)		0.003	1.42 (1.13, 1.79)	
	North-east	0.001	1.26 (1.09, 1.4)		0.162	1.18 (0.94, 1.49)	
	West	<0.001	1.64 (1.42, 1.89)		0.001	1.67 (1.25, 2.24)	
	South	<0.001	5.34 (4.76, 5.99)		<0.001	1.84 (1.48, 2.29)	
**Education**	No education		1.00	1.44		1.00	1.46
	Up to primary	0.004	1.13 (1.04, 1.22)		0.001	1.36 (1.12, 1.64)	
	Up to secondary	<0.001	1.40 (1.29, 1.53)		<0.001	1.83 (0.50, 2.24)	
	More than secondary	<0.001	1.88 (1.69, 2.08)		<0.001	2.32 (1.83, 2.93)	
**Occupation**	Govt./Non-Govt. employee		1.00	1.07		1.00	1.10
	Daily wise/causal laborer	0.008	0.85 (0.76, 0.96)		<0.001	0.47 (0.38, 0.57)	
	Self-employed	0.094	0.91 (0.82, 1.02)		<0.001	0.56 (0.46, 0.68)	
	Homemaker	0.530	1.05 (0.91, 1.21)		0.001	2.06 (1.33, 3.21)	
	Student	0.001	0.82 (0.73, 0.92)		0.001	0.49 (0.33, 0.74)	
	Others	0.025	0.86 (0.75, 0.98)		0.095	0.63 (0.37, 1.08)	
**Wealth quintile**	Q_1(poorest)		1.00	1.41		1.00	1.47
	Q_2	0.623	0.98 (0.89, 1.07)		0.121	1.17 (0.96, 1.44)	
	Q_3	0.170	1.08 (0.97, 1.19)		0.051	1.25 (0.99, 1.56)	
	Q_4	0.018	1.18 (1.03, 1.35)		0.016	1.38 (1.06, 1.79)	
	Q_5(richest)	0.108	1.10 (0.98, 1.24)		0.007	1.42 (1.10, 1.82)	

**Knowledge status about children' illness**	No		1.00	1.02			
	Yes	0.003	1.22 (1.07, 1.39)			-	

®: Reference category; AOR: Adjusted odds ratio; CI: Confidence interval; SE: Standard error, -indicates that the variable is insignificant in bivariate analysis

Table 4. Correlations between smoke-free policies in the home and workplace and SHS exposure

	Smoke-free policies at home	Smoke-free policies at workplace	SHS exposure at home	SHS exposure at workplace
Smoke-free policies at home	1.00	0.71**	-0.95**	-0.57**
Smoke-free policies at the workplace		1.00	-0.69**	-.69**
SHS exposure at home			1.00	0.60**
SHS exposure in workplace				1.00

SHS-Secondhand smoke; **Correlation is significant at the 0.01 level; *Correlation is significant at the 0.05 level

Title Page

~~Smoking norms~~Smoke-free policyies in the home and in the workplace among Indian people: Evidence from Global Adult Tobacco Survey Data-2016/2017

Mohammad Ali[1], Most. Farida Khatun[2], Tasnuva Yasmin[3], Ashis Talukder[1], Md. Maniruzzaman[1], Sharlene Alauddin[1*]

[1]Statistics Discipline, Khulna University, Khulna-9208, Bangladesh
[2]Pharmacy Discipline, Khulna University, Khulna-9208, Bangladesh
[3]Development Studies Discipline, Khulna University, Khulna-9208, Bangladesh

Contributors: All authors equal contribution

***Corresponding author**
Sharlene Alauddin
Assistant Professor
Statistics Discipline
Khulna University
Khulna-9208, Bangladesh
sharlene.kst@gmail.com

Formatted: Font: 16 pt, Bold

Formatted: Font: 16 pt, Bold

~~Smoking norms~~Smoke-free polic~~y~~ies in the home and in the workplace among Indian people: Evidence from Global Adult Tobacco Survey Data-2016/2017

Abstract

Aims: This study aimed to determine the impact of correlates on tobacco control/~~smoking norms~~smoke-free polic~~y~~ies both at home and workplace~~s~~ in India. To assess the magnitude of the ~~problem the~~problem, the relationship between ~~smoking norms~~smoke-free polic~~y~~ies and secondhand smoke (SHS) ~~-~~exposure was also explored.

Methods: Data was extracted from the Global Adult Tobacco Survey Data (GATS)-2017. It was a household survey that included people aged 15 years or older and covered all the 30 states and 2 Union Territories (UTs) in India. ~~Logistic~~ A logistic ~~Regression~~ regression model was used to determine the correlates of ~~smoking norms~~smoke-free polic~~y~~ies in ~~-~~the ~~-~~home and workplace. Additionally, Pearson correlation was used to explore the relationship between ~~smoking norms~~smoke-free polic~~y~~ies and the proportion of participants exposed to ~~secondhand smoke~~SHS ~~at~~ both at home and workplace.

Results: The overall prevalence of ~~following smoking norms~~smoke-free polic~~y~~ies in the home and workplace was 62.8% and 51.7%, respectively. ~~In this study, multiple determinants such as~~Logistic regression results illustrated that tobacco smoking status, place of residence, ~~geographical~~ region~~s~~, education, ~~employment status~~occupation, wealth quintile, and knowledge status about children's illness ~~from other people's smoking~~ were significantly associated with the respondent's ~~smoking norms~~ intention ~~intension~~ to ~~-~~live in a completely smoke-free environment both at ~~in the~~ home and workplace in India. Thise study also ~~found~~ focused on the fact that ~~the smoking norms in both the home and workplace had significantly negative association with secondhand~~ ~~smoke~~ SHS exposure ~~had~~ was significantly negatively associati~~oned~~ with a ~~smoke-free~~smoke-free ~~polic~~policy~~y~~.

Conclusion: This study will help the policymakers to promote efficient policies for improving ~~smoking norms~~smoke-free polic~~y~~ies and ~~ensuring~~ ensure a better environment both at home and workplace in India.

Formatted: Font color: Text 1
Formatted: Font color: Text 1
Formatted: Font color: Text 1
Formatted: Font color: Text 1
Formatted: Font color: Text 1
Formatted ...
Formatted: Font color: Text 1
Formatted: Font color: Text 1
Formatted: Font color: Text 1
Formatted: Font color: Text 1
Formatted: Font color: Text 1
Formatted: Font color: Text 1
Formatted: Font color: Text 1
Formatted: Font color: Text 1
Formatted: Font color: Text 1
Formatted: Font color: Text 1
Formatted: Font color: Text 1
Formatted: Font color: Text 1
Formatted: Font color: Text 1
Formatted: Font color: Text 1
Formatted: Font color: Text 1
Formatted: Font color: Text 1
Formatted: Font color: Text 1
Formatted: Font color: Text 1
Formatted: Font color: Text 1
Formatted: Font color: Text 1
Formatted: Font color: Text 1
Formatted: Font color: Text 1
Formatted: Font color: Text 1
Formatted: Font color: Text 1
Formatted: Font color: Text 1
Formatted: Font color: Text 1
Formatted: Font color: Text 1
Formatted: Font color: Text 1
Formatted: Font color: Text 1
Formatted: Font: 16 pt, Bold
Formatted: Font: 16 pt, Bold

1 Introduction

Tobacco use is a major risk factor for non-communicable diseases and one of the top preventable causes of worldwide premature deaths [1]. All forms of tobacco products, whether they are smoked, inhaled, chewed, ~~and~~ or sucked, cause serious damage to health [2]. Tobacco kills more than 8 million people each year globally, and among them, 7 million people ~~deaths~~ are ~~the result of~~died due to direct tobacco use, while around 1.2 million deaths occur due to secondhand smoke (SHS) [3]. India is the second-largest consumer of tobacco after China in the world~~, where~~ and one-third of its population uses tobacco [4, 5]. ~~Findings from~~According to the report of the Global Adult Tobacco Survey 2 (GATS ~~-~~2)-2017, ~~indicate that~~ 266.8 (28.6%) million adults use tobacco in any form in India, whereas 10.7% of people use smoke tobacco and 21.4% of people use smokeless tobacco [6]. The prevalence of male and female tobacco consumers is 42.4% and 14.2% [6]. According to the GATS 2 (2016/2017) report~~,~~, among the total of ~~secondhand smoker~~SHS, 38.7% (38.1% male vs. 39.3% female) of people takes ~~of exposure to SHS takes place~~smoke~~s~~ at home, 30.2% (32.7% male vs. 17.9% female) at workplace and 5.3% (8.1% male vs. 2.4% female) at government office, 5.6% (6.8% male vs. 4.4% female) at health care facilities, 7.4% (13.0% male vs. 1.6% female) at restaurants~~, and 13.3% (16.6% male vs. 9.9% female)~~ [6]. ~~Although w~~Asian women have a lower tendency to smoke while~~,~~ most of them and their children are mainly ~~exposure~~victims of ~~to~~ SHS ~~is the main cause for them~~ and both ~~their children to~~ suffer from various smoking-induced harms [7]. According to previous studies, about one-third of ~~nonsmoking~~ non-smokers are adults and 40% of children are exposed to SHS [7, 8]. ~~A number of~~Several ~~researches~~studies showed that ~~exposure to~~ SHS increased the risks of lung cancer, stroke, bronchitis, childhood diseases, and heart disease [9-11].

~~In order to~~To protect non-smokers from SHS, legislation was enacted by the parliament of India in 2003 entitled "Cigarettes and Other Tobacco Products Act 2003" (COTPA 2003) which prohibits smoking in public and work premises [12]. The provisions under this law were notified to the public from 2004 to 2006. The Government ~~had~~ faced many legal challenges from the tobacco industry during this period. However, in 2008, after a long legal fight and intervention of ~~the~~ civil society, a remarkable effect of these rules was found both in public and workplaces [38]. Additionally, to fulfill the commitments under the WHO Framework Convention on Tobacco Control (FCTC) and to facilitate effective implementation of COTPA 2003, the Ministry of Health and Family Welfare, Government of India, launched the National Tobacco Control Program in 2007-2008 [12]. Although there is progress in ~~smoking norms~~smoke-free polic~~y~~ies both in indoor public/workplaces and public transportation, the policies are still not comprehensive [6, 13]. All

Formatted: Width: 8.27", Height: 11.69"

Formatted: Font color: Text 1

Formatted: Font: 16 pt, Bold

Formatted: Font: 16 pt, Bold

of these rules come to the public as a Government or administrative order and there is a lack of awareness in following any legal instruments. Without strong enforcement and clear guidelines to for the citizens about their right to smoke, the implementation of these policies will remain to be largely ineffective [39]. A number ofSeveral studies have been conducted on tobacco smoking in India [14-17]. However, most of these previous studies have only focused on the prevalence and predictors of tobacco use [14-17]. Another study also investigated the exposure to SHS exposure at home and in public place places among smokers and non-smokers [18]. A study also observed that a smoke-free workplace was significantly associated with exposure to SHS exposure at home [19, 20]. A smoke-free workplace and exposure to SHS was also statistically significant [20]. Previous research has shown showed that the presence of policies that restrict smoking in the workplace and home may vary varied by geographical region, occupation, and socioeconomic status [21]. Noticeably, However, there was no no previous serious study has focused on smoking norms the risk factors affecting the functionality of smoke-free policyies both at home and in the workplace in India. This is the first study in India, to determine the influential correlates of smoking normssmoke-free policyies both both at home and in the workplace respectively. Besides, Tthis study also explores the relationship between smoking normssmoke-free policyies and exposure to SHS exposure by measuring the correlation between the state-level proportion of respondents of smoking norms and exposure to SHS at both the home and workplacepremises.

2 Materials and methods

2.1 Data

The data was extracted from the Global Adult Tobacco Survey 2 (GATS 2), which was conducted in India during August 2016-February 2017. A detailed description of the survey objectives, design, methods, and questionnaires can be found elsewhere [6]. In brief, GATS 2 is a global standard for systematically monitoring the status of using tobacco among adults and tracking key control policies. This study conducted a household survey of people aged 15 years or older, covering all the 30 states and 2 Union Territories (UTs) in India. A multistage stratified cluster sampling design was applied adopted independently to collect the sample from in each state and within the states, as well as independently in particularly from urban and rural areas to select the sample. In GATS 2, a total of **74,037** individual interviews were completed with an overall response rate of 92.9%. The analysis of the smoking normssmoke-free policyies at the workplace was restricted to the GATS 2 respondents who were working from indoors or from both indoors and outdoors but outside their home (adults). After removing the respondents with missing values, the final analysis on of the smoking normssmoke-free policyies was conducted with at home

Formatted: Font: 16 pt, Bold

Formatted: Font: 16 pt, Bold

~~focused on~~ **71,046** respondents from home and ~~people, alongside the final analysis on smoking norms at workplace focused on~~ **15,254** from workplaces. ~~people.~~

2.2 Ethics approval and consent to participate

This study ~~considers~~ utilized a secondary dataset that was publicly available and the GATS 2-2016/2017 survey was approved by the Ministry of Health and Family Welfare, Government of India. All data was completely anonymized, de-identified, and aggregated before access and analysis.

2.3 Variables of the study

~~Smoking norms~~Smoke-free ~~policy in~~ policies both at ~~the~~ home and workplace were considered as the dependent variables for this study. ~~Smoking norms at home and workplace were~~It is classified into binary categories in the following ways: the respondent was classified as living in a completely smoke-free home if he/she answered "smoking is never allowed inside your home" to the question "Which of the following best describes the rules about smoking inside your home?" If the answer was "smoking is allowed inside your home," "smoking is generally not allowed but exceptions," or "there are no rules about smoking at your home" he/she was considered as not living in a completely smoke-free home. Similarly, the respondent was classified as working in a completely smoke-free environment if he/she answered "not allowed in any indoor areas" to the question "Which of the following best describes the indoor smoking policy where you work?" If the answer was "smoking is allowed anywhere", "allowed only in some indoor areas", or "there is no policy", he/she was considered as not working in a completely smoke-free environment. The descriptions of the independent variables along with their categorizations are presented in Table 1.

In this study, to construct the wealth quintile variable, the following indicators were used: Whether the household or person living in the household enjoys the facilities of i) electricity, ii) electric fan, iii) air conditioner, iv) refrigerator, v) washing machine, vi) radio, vii) television, viii) computer/laptop, ix) internet connection, x) moped/scooter xi) car, xii) flush toilet, xiii) fixed telephone, xiv) cell telephone. Principal component analysis (PCA) has been conducted ~~and~~ to create this variable and it was divided into five categories (poorest, poorer, medium, richer, and richest) ~~on the basis of~~based on the quintiles of the first PCA scores. ~~We also collected t~~The estimated proportion of participants living in smoke-free homes~~; working in smoke-free~~ and workplaces ~~were~~was collected and ~~proportion of participants exposed to secondhand smoke at~~

Formatted: Font: 16 pt, Bold

Formatted: Font: 16 pt, Bold

~~home and workplace from the GATS 2 report for each state to measure~~ the state-level correlation between the ~~state-level~~ proportion of ~~smoking norms~~smoke-free polic~~y~~ies and ~~exposure to~~ SHS exposure at both home and workplace was also explored. GATS 2 asked the following questions to measure the ~~exposure to~~ SHS exposure at home and workplace ~~were~~ as follows: (i) How often does anyone smoke inside your home? Would you say daily, weekly, monthly, less than monthly, or never?' Respondents who indicated 'daily', 'weekly', ~~or 'monthly~~or 'monthly' were classified as exposed to SHS in the home; (ii~~)~~ ~~For~~) For the question, d~~D~~uring the past 30 days, did anyone smoke in indoor areas where you work?', r~~R~~espondents who answered~~indicated~~ 'yes' were classified as exposed to SHS in the workplace. Reviewing some pre-existing literatures, other relevant explanatory variables (given in ~~table 1~~Table 1) were selected [5,9,7,15,17,18].

Formatted: Font color: Blue

2.4 Statistical analysis

Descriptive ~~statistics~~ analysis ~~(bivariate analysis)~~ was ~~used~~ performed and expressed as frequency (percentage) ~~to explore the scenario~~ ~~using percentage and frequency~~ of ~~smoking norms~~smoke-free polic~~y~~ies both in ~~the~~ home and ~~in the~~ ~~w~~workplace~~s as well as the basic characteristics of the respondents~~. A Chi-square test was conducted ~~used t~~ to assess the bivariate association between ~~the smoking norms at both home and workplace with selected factors~~the dependent and independent variables. For multivariate analysis, a ~~Logistic~~ logistic regression model~~analysis~~ was used to ~~determine~~ ~~identify the significant correlates of smoking norms at home and in the workplace and estimated~~ ~~and to estimate~~ estimate ~~t~~the adjusted odds ratio (AOR) of the selected independent variables ~~-~~along with their correspondence p-values and 95% confidence interval ~~of AOR~~. We determined the most significant variables of smoke-free policies both at home and workplace using p-value (<0.001). In this study, the multicollinearity among different independent variables was checked by ~~using~~ variance inflation factor (VIF) values. ~~If a~~The ~~ll~~ VIF values ~~were~~ less than 10~~,~~ indicated, ~~ing there was~~ no existence of significant multicollinearity between the independent variables [22]. ~~Sampling weights were applied and weighted estimates were calculated to account for the complex study design.~~ The entire data analysis, data cleaning, and management were carried out using STATA ~~software, V~~version 12. Sampling weights were applied and the weighted estimates were calculated to consider the complex study design. Clustering and stratification were also ~~ac~~counted with the ~~for using~~ svyset command in STATA. The following variables were used to apply clustering and stratification: gatscluster, gatsstrata and gatsweight. A Pearson correlation test was carried out ~~also used~~ to explore the relationship between ~~smoking norms~~smoke-free

Formatted: Font: 16 pt, Bold

Formatted: Font: 16 pt, Bold

policyies and the proportion of SHS secondhand smoke exposure -at both home and workplace.

3 Results

3.1 Prevalence of complete smoking normssmoke-free policyies in the home and workplace

The overall prevalence of complete smoking normssmoke-free policyies -inat the home and workplace waswere 62.8% and 51.7% (See Table 2). About 25% and 48.5% of tobacco smokers follow smoking normssmoke-free policyies in their homes and workplace respectively. The major proportion of participants who lived in rural areas, were was reported to follow smoking normssmoke-free policypolicies both in their home and workplace. The Chi-square results revealed that all factors except respondents respondents' age and gender were significantly associated withh the smoking norms hhome--based smoke-free policyies, at home, whereas all other factors except the age were significantly associated with smoking normssmoke-free policyies at the workplace (p<0.001) (See Table 2).

3.2 Logistic regression analysis

Only significant factors were considered to perform in the -logistic regression models for the home and workplace. Since the VIF of all selected independent variables was less than 10, it indicated that It was noted that there erewere were no multicollinearity problems among the selected independent variables since all VIF values were less than 10 (See Table 3). We found that non-smokers were more likely to live and work in a completely smoke-free environment in their home [AOR=3.285] and workplace [AOR=1.627] compared to smokers (See Table 3). We also found that female participants were more likely to enjoy have the smoking normssmoke-free environment at their workplace. Other religious people were 1.939 times more likely to followlive in completelyly smoking normssmoke-free policpolicyy in at home compared to Hindu religious people. The pParticipants who lived in rural areas were 1.376 times for home and 1.245 times for workplace more likely to live and work in a completelyly smoke-free environment at home and workplaces respectively compared to urban counterpartsareas. The pParticipants who lived in the South region of India had the highest tendency to continue livinge and working with in completelyly smoking normssmoke-free policpolicyy at their home and workplace compared to other regions. Highly educated participants had a higher tendency to maintainkeep a completely smoke-free environment atin their home [AOR=1.879] and workplace [AOR=2.314]. Students and other participants had the lowest tendencies to live in a completely smoke-free environment at their home, while daily wise labor, self-employed, and students had the lowest tendencieswere less likely to report that their workplaces were was smoke-free compared to government/non-

Formatted: Font color: Custom Color(RGB(51,51,255))

Formatted: Font: 16 pt, Bold

Formatted: Font: 16 pt, Bold

government employees. The participants who belong came - to from rich families were 1.176 times more likely to live in a completely smoke-free in their home. homes. Whereas, the rich and richest participants had a higher tendency to report that their workplaces were completely smoke-free compared to the poorest participants. The pParticipants who were knowledgeable and careful about children's illnesses from other people's smoke had more tendencies to keep and maintain and ensueensure a complete smoking normssmoke-free policepolicyy at in their home homes compared to their counterparts.

3.3 Correlation analysis

We performed a used correlation analysis to assess the state--level relationship between a smoking normssmoke-free policy at home and the workplace with exposure to SHS exposure at home and the workplace. It was observed that the smoking normssmoke-free policyies of both in the home and in the workplace werewas significantly negatively associated with exposure to SHS exposure (See Table 4).

4 Discussion

The results of the research paper demonstrated that non-smoker participants were more likely to live in a both complete completely smoke--free in their home and workplace. Besides, elderly people composed of a varying age group from 45 onwards 65 and above are mostly tended to stay smoking freesmoke-free in their home lives h [18-20]. Similarly, people of an elder age supposedly maintained the highest rate of smokingsmoke--free policies [19-21]. Based on gender, women's likelihood to live in a smokeing--free environment at workplace environment was higher than men [18, 19, 21, 22]. Some literatures opposed this statement revealing that women's possibility to live in a completely smokingsmoke--free environment was less than their male counterparts [20, 23-25]. This phenomenon actually pointed to the fact that in the societal context, mostly men were associated with smoking and women were not even in a position to initiate a protest against this behavior [23, 26]. Additionally, female people emphasized more on a smoke--free zone in their work-place more than their opposite gender [19-22]. Married people were less tendtending to enjoy a complete non-smoking work environment as well as their home than their single counterparts [22]. Married and separated people were more habituated to smoking in their workplace [19]. Contrarily, separated persons were expected to enjoy a more smoke--free environment in their residence.

Religious factors were found to be reflecting reflected in ensuring smokingsmoke--free policies. Muslims and Christians were less likely to maintain a smoke--free environment at in their home. This might be because they are not enough concerned about their family health issues [27]. On the other hand, Muslim respondents were more likely to report that their workplaces were completely

Formatted: Indent: First line: 0.5"

Formatted: Font: 16 pt, Bold

Formatted: Font: 16 pt, Bold

smoke-free than their counterparts. Religious services both in on a social and personal levels urgedsocial and personal levels peopleurged topeople notto beingnot accustomedbe toaccustomed smokingto orsmoking or at a least consuming consume less fewer cigarettes than non-religious people [28]. People living in rural areas were more prone to live in a completely smoke-free environment both in at home and workplace comparatively compared to the urban population [18, 29, 30] which scenario was true in the context of some countries [18,19]. Existing literature agreed with the current study in the sense that on a regional basis, south Indian people possessed the highest tendency to live in a completely smoke-free environment at home environment and work place [18, 20]. People from central, east, and west were also expected to enjoy a smoke freesmoke-free environment at in their homes. Alongside the people of the east, the west had the opportunity to work in a smoke--free place/zone.

Literatures support supported the finding of this study that higher educated people are were more likely to live in a smoke--free home environment [18, 19, 21-24, 30]. But there is was contradictory information regarding higher educated people's exposure to SHS exposure in their workplace as well as at home. In one study, it iwas being pointed out that the possibility of the higher educated people to bebeing in a workplace exposed to smoking was also greater than relatively lower educated people [19, 20]. But, another study was in favor of the opposite argument [20]. Previous Literatures are were suggesting suggested the fact that people of other occupations were tended to experience a lower risk to have an unspoiled smoking contaminated job place as well as a home environment than people engaged in other government / non-government professional careers [20, 30].

People who possessed a relatively high level of income were more prone to have a complete smoke freesmoke-free household and workplace than the poorest ones [21, 24, 31]. Therefore, socioeconomic conditions contributed to enhance enhancing the cautiousness among the people. The research paper demonstrated that if a parent was a regular smoker both in at home and workplace then the chance of children to bebeing exposed to SHS would increase [20]. It could be detrimental for to children in a two-fold way. They could fall victim of to various illnesses and along with that, the risk of smoking could also increase among teenagers if their parents were accustomed to being aware about of it [31, 32]. But, if the children belonged to a highly educated and high-income family, the exposure risk would decrease [21]. Parents having this knowledge intended to enjoy a smoke-free policy- both at their home and workplace. This study also observed that smoke--free policies in the home and in the workplace werewere significantly positively associated with each other whereas both policies were significantly negatively associated with different settings of secondhand smokers.

Formatted: Font color: Blue

Formatted: Font: 16 pt, Bold

Formatted: Font: 16 pt, Bold

It ~~is~~ was indicated that smoke-~~-~~free policies played a vital role to control secondhand smoking-induced problems. However, enacting a policy does not necessarily mean that it would be completely implemented throughout the country and this is true for the COTPA also. For instance, in 2020, a study was conducted in Delhi assessing the implementation level of COTPA 2003 in 376 public places ~~of~~ in the city. Various sections of the act such as section 4 (smoking in public places-59.28%), 6-a (implementation level-68.57%), and 6-b (implementation level-52.85%) were observed to be violated to some extent [34]. The case of Bengaluru was also similar in the sense that this city also witnessed the violation of various sections of the act. The absence of a 'non-smoking sign' was 89.7% and 76% of the educational establishments had an abundance of shops selling nicotine products closer to their radius [35]. Besides the cities, the rural and tribal populations also suffer from the problem of the implementation of ~~the~~ smoke-free policies. India possesses a tribal population of more than 100 million who also belong to the highest level of smoking but because of location and associated resource deprivation, implementation of policies is poor among them [36].

However, it is prevalent from the previous discussion that some sub-groups of the respondents are more exposed to secondhand smoking than their counterparts. While looking for a causal relationship, literatures point out towards the fact that if workplaces put an embargo upon smoking, it highly decreases the rate of smoking and as a result it decreases the exposure to SHS in the workplace also [37]. Furthermore, if an individual is forced to be refrained from smoking in the workplace, it would ultimately lead to quit smoking in the home also. Besides workplace embargo upon smoking, studies also show that banning any kind of advertisement of cigarettes in the media or inclusion of smoking in movie scenes will greatly curb people's desire to smoking [38]. Now, as higher educated people and people with higher income level are generally more exposed to such workplace ban as well as awareness raising campaigns against SHS, their rate of engaging in smoking and exposure to SHS is relatively lower.

Though the existing literature ~~shows~~ showed discrepancies between the enacting and implementation of a policy, it ~~is~~ was also mentioned that a collaborative emphasis on the implementation of these policies from the institutions, policy~~-~~makers, academics, media, and civil society would increase the level of implementation [35, ~~37~~39]. Literature also ~~mentions~~ mentioned that studying the patterns of smoking from different angles will help further ~~improving~~ improve preventive tools for smoke-free policies [36]. The current study also made a similar observation that smoke-~~-~~free policies and SHS exposure ~~secondhand smokers~~ were negatively correlated. It is indicated that an improvement in the ~~smoke free~~smoke-free policies could be crucial in reducing the proportion of ~~secondhand smokers~~SHSr at every level.

Formatted: Font color: Blue

Formatted: Font color: Blue

Formatted: Font: 16 pt, Bold

Formatted: Font: 16 pt, Bold

4.1 Strength and limitations

This study was conducted based on a large national representative GATS survey. This survey ~~is~~ was considered ~~as~~ a global standard for monitoring the impact of key tobacco control policies. Earlier studies had analyzed comprehensive information on tobacco ~~smoking norms~~smoke-free polic~~y~~ies in both ~~the~~ home and workplace among different countries, but none of them investigated the situation in India ~~in spite of~~despite being at a state of high risk for tobacco. Therefore, this research work is expected to assist the policy-makers ~~to~~in imply ~~contribute to public health~~ more efficient policies~~y~~ for tobacco control in India. ~~However,~~In this study, the most significant variable~~s,~~ ~~like the~~ like the wealth index was~~ere not~~ indirectly formed. ~~Besides, we didn't explore the~~Also, the trend of ~~smoking norms~~smoke-free polic~~y~~ies at home, as well as ~~work place~~workplace, ~~in India~~ wasn't explored. However, w~~W~~e ~~also~~ didn't adjust ~~for~~ some ~~the~~ other possible confounding factors which could be explored further.

5 Conclusion

This study found that 62.8% of respondents were living under a ~~had~~ complete ~~smoking norms~~smoke-free policy at home, and 51.7% ~~for~~ at the workplace. This study also found that tobacco smoking status, place of residence, region~~s~~, education, occupation, wealth quintile, and knowledge status about the children's illness from other people's smoking were significantly associated with the complete ~~smoking norms~~smoke-free polic~~y~~ies in ~~the~~ home, while all these significant factors ~~were found in the home,~~ along with gender, were also associated with the complete ~~smoking norms~~smoke-free polic~~y~~ies for the workplace in India. ~~Smoking norms~~Smoke-free polic~~y~~ies in both ~~the~~ home and workplace were significantly negatively associated with ~~exposure to~~ SHS exposure. Indian authorities should emphasize these findings as a supporting document while~~en~~ implementing ~~making~~ tobacco control policies.

Acknowledgements

The authors would like to acknowledge the authority of the GATS 2-2016/2017.

Availability of data and materials

The data set used in this study will be available from the website of the Centers for Disease Control and Prevention (CDCP). ~~In order to~~To gain access to the data files, you have to complete the form. The data set is available from the following link.

nccd.cdc.gov/GTSSDataSurveyResources/Ancillary/DataReports.aspx?CAID=2

Formatted: Font: 16 pt, Bold

Formatted: Font: 16 pt, Bold

Competing interests

None declared

Funding

The authors ~~do~~ did not receive any funds for this study.

References

1. Alwan A. Global status report on noncommunicable diseases 2010. 2011: World Health Organization.
2. Bauer UE, Briss PA, Goodman RA, Bowman BA. Prevention of chronic disease in the 21st century: elimination of the leading preventable causes of premature death and disability in the USA. The Lancet. 2014; 384(9937):45-52.
3. World Health Organization. WHO report on the global tobacco epidemic, 2019: offer help to quit tobacco use: executive summary, in WHO report on the global tobacco epidemic, 2019: offer help to quit tobacco use: executive summary. 2019.
4. Ministry of Health and Family Welfare, G.o.I., Global Adult Tobacco Survey India, 2009-2010. New Delhi, India. 2010.
5. Kaur J, Jain DC. Tobacco control policies in India: implementation and challenges. Indian journal of public health. 2011;55(3):220.
6. Tata Institute of Social Sciences (TISS), M.M.o.H.a.F.W., Government of India., Global Adult Tobacco Survey GATS 2 India 2016-17. 2017.
7. Öberg M, Jaakkola MS, Woodward A, Peruga A, Prüss-Ustün A. Worldwide burden of disease from exposure to second-hand smoke: a retrospective analysis of data from 192 countries. The lancet. 2011;377(9760):139-46.
8. Eriksen MJ, Mackay, H Ross. The tobacco atlas. 2013: American Cancer Society.
9. Oberg M. Second hand smoke: Assessing the burden of disease at national and local levels. 2010
10. Samet JM, Avila-Tang E, Boffetta P, Hannan LM, Olivo-Marston S, Thun MJ, et al. Lung cancer in never smokers: clinical epidemiology and environmental risk factors. Clinical Cancer Research. 2009;15(18):5626-45.
11. Fischer F, A Kraemer. Meta-analysis of the association between second-hand smoke

Formatted: Font: 16 pt, Bold

Formatted: Font: 16 pt, Bold

exposure and ischaemic heart diseases, COPD and stroke. BMC Public Health, 2015; 15(1): 1202.

12. Schwartz RL, Wipfli HL, Samet JM. World No Tobacco Day 2011: India's progress in implementing the framework convention on tobacco control. The Indian journal of medical research. 2011;133(5):455.
13. Mistry R, Dasika A. Antenatal tobacco use and secondhand smoke exposure in the home in India. Nicotine and Tobacco Research. 2018;20(2):258-61.
14. Mohan P, Lando HA, Panneer S. Assessment of tobacco consumption and control in India. Indian Journal of Clinical Medicine. 2018;9:1179916118759289.
15. Guindon GE, Nandi A, Chaloupka IV FJ, Jha P. Socioeconomic differences in the impact of smoking tobacco and alcohol prices on smoking in India. National Bureau of Economic Research. 2011.
16. Prasad R, Suryakant RG, Singhal S, Dawar R, Agarwal GG. A case-control study of tobacco smoking and tuberculosis in India. Annals of thoracic medicine. 2009;4(4):208.
17. Mistry R, Pednekar MS, Gupta PC, Raghunathan TE, Appikatla S, Puntambekar N, et al. Longitudinal study of adolescent tobacco use and tobacco control policies in India. BMC public health. 2018;18(1):815.
18. Tripathy, J.P. Secondhand smoke exposure at home and public places among smokers and non-smokers in India: findings from the Global Adult Tobacco Survey 2016–17. *Environ Sci Pollut Res* 27, 6033–6041 (2020).
19. Tripathy, J. P. (2020). Smoke-free workplaces are associated with smoke-free homes in India: evidence for action. *Environmental Science and Pollution Research*, *27*(33), 41405-41414.
20. Lee JT, Agrawal S, Basu S, Glantz SA, Millett C. Association between smoke-free workplace and second-hand smoke exposure at home in India. Tobacco control. 2014 ;23(4):308-12.
21. Berg CJ, Daley CM, Nazir N, Cully A, Pacheco CM, Buchanan T, et al. Smoke-free policies in the workplace and in the home among American Indians. Journal of health disparities research and practice. 2012;5(2):7.
22. Tripathy JP, Verma M. Impact of Health Warning Labels on Cigarette Packs in India: Findings from the Global Adult Tobacco Survey 2016–17. Behavioral Medicine. 2020; 20:1-0.
23. Ayo-Yusuf OA, Olufajo O, Agaku IT. Exposure to secondhand smoke and voluntary adoption of smoke-free home and car rules among non-smoking South African adults. BMC public health. 2014;14(1):580.
24. Max W, Sung HY, Shi Y. Exposure to secondhand smoke at home and at work in California. Public health reports. 2012;127(1):81-8.
25. Singh A, Sahoo N. Urban–rural differentials in the factors associated with exposure to second-hand smoke in India. BMJ open. 2013;3(11).
26. Nazar GP, Lee JT, Glantz SA, Arora M, Pearce N, Millett C. Association between being employed in a smoke-free workplace and living in a smoke-free home: evidence from 15 low and middle income countries. Preventive Medicine. 2014; 59:47-53.

Formatted: Font: 16 pt, Bold
Formatted: Font: 16 pt, Bold

27. Bonevski B, Paul C, Jones A, Bisquera A, Regan T. Smoky homes: Gender, socioeconomic and housing disparities in second hand tobacco smoke (SHS) exposure in a large population-based Australian cohort. Preventive medicine. 2014; 60: 95-101..

28. Awawdi K, Steiner H, Green MS, Zelber-Sagi S. Association between second-hand smoking and acute coronary heart disease among Arab women with multiple risk factors. The European Journal of Public Health. 2016;26(1):141-5.

29. Olasky SJ, Levy D, Moran A. Secondhand smoke and CVD in low-and middle-income countries: A case for action. Global heart. 2012;7(2):151-60.

30. Yong HH, Hamann SL, Borland R, Fong GT, Omar M. Adult smokers' perception of the role of religion and religious leadership on smoking and association with quitting: A comparison between Thai Buddhists and Malaysian Muslims. Social science & medicine. 2009;69(7):1025-31.

31. Koenig HG, George LK, Cohen HJ, Hays JC, Larson DB, Blazer DG. The relationship between religious activities and cigarette smoking in older adults. The Journals of Gerontology Series A: Biological Sciences and Medical Sciences. 1998;53(6):M426-34.

32. Kaleta D, Polanska K, Usidame B. Smoke-free workplaces are associated with protection from second-hand smoke at homes in nigeria: evidence for population-level decisions. BioMed research international. 2015.

33. Kabir Z, Alpert HR, Goodman PG, Haw S, Behm I, Connolly GN, et al. Effect of smoke-free home and workplace policies on second-hand smoke exposure levels in children: an evidence summary. Pediatric Health. 2010;4(4):391-403.

34. Ali, I., Patthi, B., Singla, A., Dhama, K., Muchhal, M., Rajeev, A., ...& Khan, A. (2020). Assessment of implementation and compliance of (COTPA) Cigarette and Other Tobacco Products Act (2003) in open places of Delhi. *Journal of Family Medicine and Primary Care*, 9(6), 3094.

35. Habbu, S. G., &Krishnappa, P. (2015). Assessment of implementation of COTPA-2003 in Bengaluru city, India: A cross-sectional study. *Journal of Indian Association of Public Health Dentistry*, 13(4), 444.

36. Mohan, P., Lando, H. A., &Panneer, S. (2018). Assessment of tobacco consumption and control in India. *Indian Journal of Clinical Medicine*, 9, 1179916118759289.

37. Farrelly, M. C., Evans, W. N., & Sfekas, A. E. (1999). The impact of workplace smoking bans: results from a national survey. Tobacco Control, 8(3), 272-277.

~~36.~~38. Yang, T., Rockett, I. R., Li, M., Xu, X., & Gu, Y. (2012). Tobacco advertising, environmental smoking bans, and smoking in Chinese urban areas. Drug and alcohol dependence, 124(1-2), 121-127.

~~37.~~39. Panda, B., Rout, A., Pati, S., Chauhan, A. S., Tripathy, A., Shrivastava, R., &Bassi, A. (2012). Tobacco control law enforcement and compliance in Odisha, India-Implications for tobacco control policy and practice.

Formatted: Condensed by 0.15 pt

Formatted: Font: 12 pt

Formatted: Font: 16 pt, Bold

Formatted: Font: 16 pt, Bold

38. Revised Smoke free Rules. Notification in the Official Gazette GSR 417 (E) dated 30 th May, 2008.

Kaur, J., & Jain, D. C. (2011). Tobacco control policies in India: implementation and challenges. Indian journal of public health, 55(3), 220.

39.

Formatted: Indent: Left: 0.42", No bullets or numbering

Formatted: Highlight

Table 1. Descriptions of the independent variables along with their categorizations

Factors	GATS asked the following questions	Categorizations
Tobacco Smoking smoking status	Does this person currently smoke tobacco, including bidis, cigarettes, hukkah, cigars, and pipesand pipes?	1= Smoker, 2= Non-smoker
Age (in years)	What is the age of the selected person?	1=15-30, 2= 31-45, 3= 46-60, 4=More than 60
Gender	Record the gender of the selected person	1= Male= , 2Male, 2= Female= Female
Marital status	What is your marital status?	1= single, 2Single, 2= Married= , 3Married, 3= Separated/others
Religion	What is your religion?	1=Hindu,2=Muslim,3=Christian, 4=Others
Place of residence	Residence status	1= Urban, 2=Rural
Regions	National geographical regions	1=North, 2= Central, 3= East, 4= North-east, 5= West, 6= South
Education	What is the highest level of education you completed?	1=No education,2=Up to primary, 3=Up to secondary, 4= More than secondary
Occupation	Which of the following best describes your main work status over the past 12 months?	1=Govt./Non-Govt. employee,2=Daily wise/causal laborer, 3= Self-employed, 4=Homemaker, 5= Student, 6= Others
Wealth quintile	How would you describe the status of your Wealth	1= Poorest, 2= Poorer, 3= Middle, 4= Richer, 5= Richest

Formatted: Left

Formatted: Font: Not Bold

Formatted: Font: Not Bold

Formatted: Font: 16 pt, Bold

Formatted: Font: 16 pt, Bold

	quintile?	
Knowledge~~able~~ status ~~of the~~ about children' illness	Based on what you know or believe, does smoking tobacco cause serious illness among the children?	1= No, 2= Yes

Formatted Table

Formatted: Font: 16 pt, Bold

Formatted: Font: 16 pt, Bold

Table 2. Correlates of ~~smoking norms~~smoke-free polic~~y~~ies in the home and the workplace in India

Characteristics	~~Smoking norms~~Smoke-free ~~policy~~ ~~in~~policies in					
	~~Home (~~Home (n= 15,254)			Workplace (n= 71,046)		
	Yes %	No, %	p-value	Yes, %	No, %	p-value[1]
Overall	62.8	37.2		51.7	48.3	
**Tobacco ~~Smoking~~ smoking status**			<0.001			<0.001
Smoker	28.5	71.5		48.5	51.5	
Non-smoker	56.1	43.9		66.9	33.1	
**Age (in year)**			0.944			0.676
**15-30**	51.8	48.2		63.6	36.4	
31-45	51.8	48.2		61.8	38.2	
46-60	51.3	48.7		62.7	37.3	
More than 60	51.5	48.5		63.2	36.8	
**Gender**			0.205			<0.001
Male	52.2	47.8		61.6	38.4	
Female	51.1	48.9		68.9	31.1	
**Religion**			<0.001			0.0086
Hindhu	51.9	48.1		63.5	36.5	
Muslim	45.4	54.6		57.1	42.9	
Christian	65.4	34.6		64.6	35.4	
Others	35.0	65.0		68.5	31.5	
**Marital status**			<0.001			<0.001
Single	56.0	44.0		69.0	31.0	
Married	50.2	49.8		60.9	39.1	
Separated/others	52.6	47.4		58.9	41.1	
**Place of residence**			<0.001			<0.001
Urban	46.1	53.9		56.0	44.0	
Rural	62.1	37.9		70.8	29.2	
**Region**			<0.001			<0.001
North	56.3	53.7		62.3	37.7	
Central	38.3	61.7		52.7	47.3	
East	43.9	56.1		58.1	41.9	
North-east	43.3	56.7		55.9	44.1	
West	56.6	43.4		74.7	25.3	
South	77.9	22.1		72.2	27.8	
**Education**			<0.001			<0.001
No education	41.5	58.5		41.5	58.5	
Up to primary	46.4	53.6		50.8	49.2	
Up to secondary	53.6	46.4		64.8	35.2	
More than secondary	65.1	34.9		76.3	23.7	
**Occupation**			<0.001			<0.001
Govt./ Non-Govt. employee	60.7	39.3		75.5	24.5	
Daily wage/ causal labourer	46.8	53.2		46.6	53.4	
Self employed	49.1	50.9		58.2	41.8	
Homemaker	59.6	40.4		84.0	16.0	
Student	50.5	49.5		52.8	47.2	
Others ~~(a)~~	50.1	49.9		58.0	42.0	
**Wealth quintile (b)**			<0.001			<0.001
Q_1 (poorest)	39.1	60.9		46.1	53.9	
Q_2	48.7	51.3		56.7	43.3	
Q_3	54.5	45.5		62.1	37.9	
Q_4	62.8	37.2		74.2	25.8	
Q_5 (richest)	59.6	40.4		72.5	27.5	
**Knowledge status about children' illness~~Other people's smoking cause serious illness in children~~**			<0.001			0.484
Yes	52.1	47.9		62.9	37.1	
No	43.4	56.6		60.0	40.0	

Q1: Poorest; Q2: Poorer; Q3: Middle; Q4: Richer; Q5: Richest.

Formatted: Font: Not Bold

Formatted: Font: Bold

Formatted: Font: 16 pt, Bold

Formatted: Font: 16 pt, Bold

Table 3. Multivariable logistic regression results

Variable		~~Smoking norms~~Smoke-free ~~policy~~ ~~- in~~policies in					
		Home			Workplace		
		p-value	AOR (95% CI of AOR)	VIF	p-value	AOR (95% CI of AOR)	VIF
**Tobacco smoking status**	Smoker	~~1~~	1.00	1.05	~~1~~	1.00	1.11
	Non-smoker	<0.001	3.29 (3.00,3.59)		**<0.001**	1.63 (1.40, 1.89)	
**Gender**	Male		-		~~**1**~~	1	1.11
	Female		-		**0.048**	1.23 (1.00, 1.51)	
**Marital status**	Single	~~1~~	1.00	1.13	~~1~~	1.00	1.06
	Married	0.420	0.96 (0.87, 1.06)		0.604	0.95 (0.79, 1.14)	
	Separated/others	0.274	1.08 (0.94, 1.24)		0.934	0.98 (0.68, 1.43)	
**Religion**	Hindhu	~~1~~	1.00	1.01	~~1~~	1.00	1.01
	Muslim	0.074	0.89 (0.77, 1.01)		0.510	1.07 (0.88, 1.31)	
	Christian	0.901	0.99 (0.81, 1.21)		0.084	0.71 (0.48, 1.05)	
	Others	<0.001	1.94 (1.65, 2.28)		0.589	1.09 (0.78, 1.55)	
**Place of residence**	Urban	~~1~~	1.00	1.21	~~1~~	1.00	1.19
	Rural	<0.001	1.38 (1.25, 1.51)		0.015	1.25 (1.04, 1.48)	
**Region**	North	~~1~~	1.00	1.01	~~1~~	1.00	1.01
	Central	0.434	0.95 (0.84, 1.08)		0.170	0.87 (0.71, 1.06)	
	East	<0.001	1.25 (1.11, 1.41)		0.003	1.42 (1.13, 1.79)	
	North-east	0.001	1.26 (1.09, 1.4)		0.162	1.18 (0.94, 1.49)	
	West	<0.001	1.64 (1.42, 1.89)		0.001	1.67 (1.25, 2.24)	
	South	<0.001	5.34 (4.76, 5.99)		<0.001	1.84 (1.48, 2.29)	
**Education**	No education	~~1~~	1.00	1.44	~~1~~	1.00	1.46
	Up to primary	0.004	1.13 (1.04, 1.22)		0.001	1.36 (1.12, 1.64)	
	Up to secondary	<0.001	1.40 (1.29, 1.53)		<0.001	1.83 (0.50, 2.24)	
	More than secondary	<0.001	1.88 (1.69, 2.08)		<0.001	2.32 (1.83, 2.93)	
**Occupation**	Govt./Non-Govt. employee	~~1~~	1.00	1.07	~~1~~	1.00	1.10
	Daily wise/causal laborer	0.008	0.85 (0.76, 0.96)		<0.001	0.47 (0.38, 0.57)	
	Self-employed	0.094	0.91 (0.82, 1.02)		<0.001	0.56 (0.46, 0.68)	
	Homemaker	0.530	1.05 (0.91, 1.21)		0.001	2.06 (1.33, 3.21)	
	Student	0.001	0.82 (0.73, 0.92)		0.001	0.49 (0.33, 0.74)	
	Others	0.025	0.86 (0.75, 0.98)		0.095	0.63 (0.37, 1.08)	
**Wealth quintile**	Q_1(poorest)	~~1~~	1.00	1.41	~~1~~	1.00	1.47
	Q_2	0.623	0.98 (0.89, 1.07)		0.121	1.17 (0.96, 1.44)	
	Q_3	0.170	1.08 (0.97, 1.19)		0.051	1.25 (0.99, 1.56)	
	Q_4	0.018	1.18 (1.03, 1.35)		0.016	1.38 (1.06, 1.79)	
	Q_5(richest)	0.108	1.10 (0.98, 1.24)		0.007	1.42 (1.10, 1.82)	
**Knowledge status about children' illness~~Other people's Smoking cause serious Illness in children~~**	No	~~1~~	1.00	1.02	~~1~~		
	Yes	0.003	1.22 (1.07, 1.39)			-	

®: Reference category; AOR: Adjusted odds ratio; CI: Confidence interval; SE: Standard error, -indicates that the variable is insignificant in bivariate analysis

Formatted: Font: Bold

Formatted: Indent: Left: 0"

Formatted: Font: 16 pt, Bold

Formatted: Font: 16 pt, Bold

Table 4. Correlations between ~~smoking norms~~smoke-free polic~~y~~ies in the home and workplace and ~~secondhand smoke~~SHS exposure

	~~Smoking norms~~Smoke-free polic~~y~~ies at home	~~Smoking norms~~Smoke-free polic~~y~~ies at workplace	SHS exposure at home	SHS exposure at workplace
~~Smoking norms~~Smoke-free polic~~y~~ies at home	1.00	0.71**	-0.95**	-0.57**
~~Smoking norms~~Smoke-free polic~~y~~ies at the workplace		1.00	-0.69**	-.69**
SHS exposure at home			1.00	0.60**
SHS exposure ~~at~~ in workplace				1.00

SHS-Secondhand smoke; **Correlation is significant at the 0.01 level; *Correlation is significant at the 0.05 level

Formatted: Indent: Left: 0"

Formatted: Font: 16 pt, Bold

Formatted: Font: 16 pt, Bold

Authors' Response to Reviewers

Manuscript ID: PONE-D-20-41034R3

Title: Smoke-free policies in the home and in the workplace among Indian people: Evidence from Global Adult Tobacco Survey Data-2017

Dear Respected Editor-in-Chief,

First of all, we thank the reviewer and the editor for their useful suggestions for improving the manuscript. We have now revised the manuscript accordingly. Thank you once again and looking forward to the final acceptance in your esteemed journal.

Reviewer (R3):

The paper has been improved but important limitations remain:

(R3-1): We still miss a justification of the variables' choice. Mentioning that they have been used in the literature is not enough. We must understand which mechanisms may lead subgroups to adopt stricter norms or not. In the Discussion, no good explanations are presented for some groups adopting stricter norms (e.g., more educated or richer people).

Authors: Thank you for your valuable suggestions and giving us an opportunity to improve the revised manuscript. We try to add the justification of including various explanatory variables for this research work and modify the discussion section to include the possible reasons for adopting stricter norms among some specified groups (e.g., more educated or richer people).

(R3-2): Since this information is missing about potential causal mechanisms, the Discussion does not provide relevant insights for decision making.

Authors: Thanks again for your valuable suggestions. We have focused on this suggestion and rewritten some points in the discussion section to make the potential causal mechanisms clear. Thanks for understanding in advance.

(R3-2): The text needs a careful revision of English language.

Authors: Thank you for your comment. We have given a special attention to maintaining the standard English language for this article. Thanks for understanding in advance.

Dear Reviewer#3

Thank you very much for your encouragement, kind guidance, and support in helping the authors improve the manuscript.
We are truly grateful for your time and support.
Kind Regards,
The Authors of the manuscript.

Reviewer (R4):

(R4-1): I beg to differ with the other reviewer who suggested changing the term **"smoke-free policy" to "smoking norms"**. In my opinion, using the term **"smoke-free policy"** is more accurate.

Authors: We highly appreciate your valuable comments. We have changed the term "smoke-free policy" to "smoking norms" throughout the manuscript. Thanks for understanding in advance.

(R4-2): In Table 3, please place the number 1 to indicate the reference category in the AOR column instead of the p value column.

Authors: Thank you for your valuable suggestions. We regret making such a great mistake. Now, we have fixed it and updated the revised manuscript. Thanks again.

(R4-3): All other issues were addressed except for language, which still needs correcting. Example: In Discussion, Para 3-"Literatures are suggesting the fact than people engaged in other government/non government professional careers" needs to be rewritten. In its current form, the meaning of this sentence is unclear.

Authors: Thank you for your opinion. This point has been carefully addressed and updated in the revised manuscript. Moreover, we have given special attention to maintaining the standard English language for this article. Thanks again for your undderstadning in advance.

Dear Reviewer#4

Thank you very much for your encouragement, kind guidance, and support in helping the authors improve the manuscript.
We are truly grateful for your time and support.
Kind Regards,
The Authors of the manuscript.
