# Supplementary material for: Smoke-free status of homes and workplaces among Indian people: Evidence from Global Adult Tobacco SurveyData-2016/2017
Source: PLoS One. 2023 Feb 23;18(2):e0282138. doi: 10.1371/journal.pone.0282138 (PMC9949653; doi:10.1371/journal.pone.0282138)
Supplement: Attachment — Submitted filename: Review_Gaurang.docx [file pone.0282138.s001.docx]

I have read this paper with interest. I have some observations. There have been some recent (2020) publications looking at SHS exposure at homes and workplaces. These have been listed below for the authors. Given that, the authors need to better explain the rationale for this study. Substantial edits are required throughout the manuscript (see below). The manuscript requires a thorough copy-editing for English language, structure and meaning that is conveyed.

**Abstract**

The authors say “and determine and determine the association between and within smoke-free policies at different setting where secondhand smoking occurs.” This is not very clear. This could be reworded to make this part of the objective clearer.

The introductory sentences in the abstract could be removed and more detail could be provided on data source and methods.

Description of the results is unclear and could be clarified. The authors mention factors correlated with complete smoke-free policies at home and at workplace and subsequent sentence says therefore both were significantly associated. This is unclear. The authors also mention that both policies were significantly negatively associated with different settings of secondhand smokers. This part “different settings” also should be clarified. What are these different settings? What analysis was done.

Overall, abstract requires to be reworked significantly to make objectives, data sources, methods, results and conclusion/policy implication clearer.

**Introduction**

Page 3

- National Tobacco Control Programme was established in 2007-08.
- Smoking zones are allowed only in hotels with more than 30 rooms, restaurants with more than 30 seats and airports.
- ‘Open spaces’ could be reworded as several open spaces are covered e.g. bus-stops, stadiums etc.
- Reference/s should be provided for “some previous country- or community-based researches shown that the presence of policies that restrict smoking in the workplace and home may vary by geographical region, occupation, industry and socioeconomic status.” And if this is in the context of GATS, what does ‘industry’ mean here?
- Not able to understand here the way authors are trying to justify the importance of studying context of implementation of policies. Yes, the level of implementation may vary by geographic region (and GATS does not measure level of implementation). I believe, at best, the authors are mentioning about correlates of smoke-free policies and the variation therein (between urban/rural geographic regions, across occupational of SES categories). The rationale for studying associations at state-level also needs proper justification. So current justification here needs to be revised.
- The authors may also want to have a look at these published papers to clarify their justification for this study:

1) <https://pdfs.semanticscholar.org/870c/449e35399633710a9ad4b223e46947102af1.pdf?_ga=2.118417226.2146522655.1614069121-810001830.1607255405>
2) <https://link.springer.com/article/10.1007/s11356-019-07341-x>
3) <https://link.springer.com/article/10.1007/s11356-020-10107-5>

**Methods**

Page 5, section 2.2

Why only knowledge about “children’s illness” would be included as an independent variable? SHS exposure causes illness among all.

Authors could provide more information about independent variables and groupings. The authors just mention ‘household assets’ but how was SES variable derived from that? Perhaps a supplementary table with variable definitions could be useful.

Section 2.3 (Data analysis) – The authors used logistic regression to study the correlates of living in smoke-free homes and working in smoke-free workplaces. Some language edits are required here for clarity. Did the authors perform any multi-collinearity diagnostics? It should be reported. The analysis for state-level within and between analysis needs further description for clarity. This seems relevant because there is no description of how the “State” variable was treated in the analysis. From the results presented (Table 3 and Correlation matrix), it seems only overall National level estimates have been used and presented.

**Results**

In tables 1 and 2 the authors could club the other three categories for both outcomes unless they are going into their details. In Table 3, why are gender (for SF at home) and age groups (for SF at workplace) missing as In Table 1 gender is significantly associated with the outcome and in Table 2, age group is also significantly associated (p<0.05). Authors should check this.

Ok. At the end of results section and after seeing the correlation matrix, one realizes that it is not the “different settings of second hand smokers” but it is SHS exposure (at home, workplace and public places). This needs to be clarified in the methods and also in the abstract. “Different settings of secondhand smokers” is confusing. Again, when SHS exposure at public places has not been discussed throughout the paper, what is the rationale for bringing that in in the correlation matrix?

**Discussion**

Page 7

- First line of discussion – Non-smoker participants were more likely (NOT priorities)….
- Well, most of the results and discussion deals with for e.g. females vs. males are more likely to live in smoke-free homes/workplaces and similar findings. What is not discussed in Discussion section, is the policy implications of the findings. In India, the smoke-free legislation is a national law (under COTPA 2003) and probably the level of implementation varies across geographic areas (rural/urban, national regions, states). So how are the findings specifically relevant to the national policy scenario? That needs to be discussed in detail.
