# Supplementary material for: Smoke-free status of homes and workplaces among Indian people: Evidence from Global Adult Tobacco SurveyData-2016/2017
Source: PLoS One. 2023 Feb 23;18(2):e0282138. doi: 10.1371/journal.pone.0282138 (PMC9949653; doi:10.1371/journal.pone.0282138)
Supplement: Attachment — Submitted filename: Response to Reviewer-Ver1.docx [file pone.0282138.s002.docx]

**Authors’ Response to Reviewers**

Manuscript ID: PONE-D-20-41034

**Title: Smoke-free policies in the home and in the workplace among Indian people: Evidence from Global Adult Tobacco Survey Data-2017**

Dear Respected Editor-in-Chief,

First of all, we thank the reviewer and the editor for their useful suggestions for improving the manuscript. We have now revised the manuscript accordingly. Thank you once again and looking forward to the final acceptance in your esteemed journal.

**Reviewer #1:**

**Reviewer#1-1**: I have read this paper with interest. I have some observations. There have been some recent (2020) publications looking at SHS exposure at homes and workplaces. These have been listed below for the authors. Given that, the authors need to better explain the rationale for this study. Substantial edits are required throughout the manuscript (see below). The manuscript requires a thorough copy-editing for English language, structure and meaning that is conveyed.

**Authors:** Thank you for your suggestions. The manuscript has been revised and modified by considering your instruction.

**Reviewer#1-2**: In Abstract, the authors say “and determine and determine the association between and within smoke-free policies at different setting where secondhand smoking occurs.” This is not very clear. This could be reworded to make this part of the objective clearer.

**Authors:** Thank you very much for asking this question. We are totally agreed with this reviewer. We update the objectives in the revised manuscript as follows:

**Old objective:** To determine the correlation between smoke-free policies in the home and workplace and determine the association between and within smoke-free policies at different setting where secondhand smoking occurs

**New Objective:** To determine the correlates of smoke-free policies in the home and workplace along with assessing the relationship between smoke-free policies and secondhand smoker’s exposure.

**[Revised Manuscript Page #2; Abstract: Line#: 1-3]**

**Reviewer#1-3**: The introductory sentences in the abstract could be removed and more detail could be provided on data source and methods.

**Authors:** Thank you very much for asking this question. We are already removed the introductory sentences are removed from abstract and added the data source and methods more details in the abstract in the revised manuscript. Thanks again.

**[Revised Manuscript Page #2]**

**Reviewer#1-4**: Description of the results is unclear and could be clarified. The authors mention a factor correlated with complete smoke-free policies at home and at workplace and subsequent sentence says therefore both were significantly associated. This is unclear. The authors also mention that both policies were significantly negatively associated with different settings of secondhand smokers. This part “**different settings**” also should be clarified. What are these different settings? What analysis was done?

**Authors:** Thank you very much for asking this question. Actually, this study presents a comparative study of completely smoke-free policy between home and workplace. **This study also assess the relationship between smoke-free polices and secondhand smokers.** To fill-up the objectives, we have implemented logistic regression model to determine the correlates of smoke-free policies in the home and workplace. We have also implemented Pearson correlation co-efficient to explore the relationship between smoke-free policies and proportion of secondhand smoker’s exposure at both home and workplace. We are updated the abstract in details in the revised manuscript. Thanks again.

**Old abstract:** Tobacco smoking is one of the biggest global health concerns of this century. Secondhand smoking has a significant contribution to the increasing burden of cancers, chronic diseases and associated mortality. India is the second largest consumer of tobacco after China in the world. A number of researches were conducted in India related to tobacco for improving tobacco control policies. However, this is the first study to determine the correlation between smoke-free policies in the home and workplace and determine the association between and within smoke-free policies at different setting where secondhand smoking occurs. Results found that tobacco smoking status, place of residence, geographical regions, education, employment status, household asset and knowledge status about children being affected the rate of second hand smoking, and were significantly correlated at p < 0.001 with the complete smoke-free policies both in the home and in the workplace in India. Therefore, smoke free policies in the home and in the workplace were significantly positively associated with each other whereas both policies were significantly negatively associated with different settings of secondhand smokers.

***New abstract:***

**Aim:** *The aim of this study was to determine the correlates of smoke-free policies in the home and workplace along with assessing the relationship between smoke-free policies and secondhand smoker’s exposure.*

***Methods:*** *Data was collected from Global Adult Tobacco Survey Data (GATS)-2017. It was a household survey that included the people aged 15 years or older and covered all the 30 states and 2 Union Territories (UTs) in India. Logistic Regression model was used to determine the correlates of smoke-free policies in the home and workplace. Pearson correlation was also used to explore the relationship between smoke-free policies and proportion of secondhand smoker’s exposure at both home and workplace.*

***Results:*** *Results found that tobacco smoking status, place of residence, geographical regions, education, employment status, household asset, and knowledge status about the children’s illness from other people’s smoking were significantly correlated at p<0.001 with the complete smoke-free policies both in home and workplace in India. The study also found that smoke-free policies in both home and workplace were significantly negatively associated with secondhand smoker’s exposure.*

***Conclusion:*** *This study is expected to help the policymakers to improve smoke-free policies and ensure a better environment both in home and workplace.*

**[Revised Manuscript Page #2]**

**Reviewer#1-5**: Overall, abstract requires to be reworked significantly to make objectives, data sources, methods, results and conclusion/policy implication clearer.

**Authors:** Thank you very much for asking this question and give us an opportunity to improve the revised manuscript. We are totally agreed with this reviewer. We are already added in details of the objectives, data sources, results and conclusion/policy implication in the abstract of the revised manuscript as follows:

**“*Aim:*** *The aim of this study was to determine the correlates of smoke-free policies in the home and workplace along with assessing the relationship between smoke-free policies and secondhand smoker’s exposure.*

***Methods:*** *Data was collected from Global Adult Tobacco Survey Data (GATS)-2017. It was a household survey that included the people aged 15 years or older and covered all the 30 states and 2 Union Territories (UTs) in India. Logistic Regression model was used to determine the correlates of smoke-free policies in the home and workplace. Pearson correlation was also used to explore the relationship between smoke-free policies and proportion of secondhand smoker’s exposure at both home and workplace.*

***Results:*** *Results found that tobacco smoking status, place of residence, geographical regions, education, employment status, household asset, and knowledge status about the children’s illness from other people’s smoking were significantly correlated at p<0.001 with the complete smoke-free policies both in home and workplace in India. The study also found that smoke-free policies in both home and workplace were significantly negatively associated with secondhand smoker’s exposure.*

***Conclusion:*** *This study is expected to help the policymakers to improve smoke-free policies and ensure a better environment both in home and workplace.”*

**[Revised Manuscript Page #2]**

Thank again for giving us an opportunity to improve the revised manuscript.

**Reviewer#1-6**: **Introduction,** Page 3**:** National Tobacco Control Programme was established in 2007-08.

**Authors:** Thank again this reviewer for giving us an opportunity to improve the revised manuscript. We are totally agreed with this reviewer that National Tobacco Control Programme was established in 2007-08. We fix it and update the revised manuscript.

**[Revised Manuscript Page #3, Section #1]**

**Reviewer#1-7**: **Introduction,** Page 3**:** Smoking zones are allowed only in hotels with more than 30 rooms, restaurants with more than 30 seats and airports.

**Authors:** Thank again this reviewer for giving us an opportunity to improve the revised manuscript. According to your overall suggestions, we exclude this statement from our revised manuscript to rearrange the introduction. Thanks again.

**Reviewer#1-8**: **Introduction,** Page 3**:** ‘Open spaces’ could be reworded as several open spaces are covered e.g. bus-stops, stadiums etc.

**Authors:** Thank again this reviewer for giving us an opportunity to improve the revised manuscript. According to your overall suggestions, we exclude this statement from our revised manuscript to rearrange the introduction. Thanks again.

**Reviewer#1-9**: **Introduction,** Page 3**:** Reference/s should be provided for “some previous country- or community-based researches shown that the presence of policies that restrict smoking in the workplace and home may vary by geographical region, occupation, industry and socioeconomic status.” **And if this is in the context of GATS, what does ‘industry’ mean here?**

**Authors:** Thank you for your kind suggestions. Some words are reworded according to your advice. Unfortunately Reference/s for some statements which are written on the basis of some previous evidence based literature was missed. We had included the necessary references in the required places in the revised manuscript.

In the context of GATS, we are totally agreed with this reviewer. So, we omit the words “industry” from the revised manuscript. Thanks again

**Reviewer#1-10**: **Introduction,** Page 3**:** Not able to understand here the way authors are trying to justify the importance of studying context of implementation of policies. Yes, the level of implementation may vary by geographic region (and GATS does not measure level of implementation). I believe, at best, the authors are mentioning about correlates of smoke-free policies and the variation therein (between urban/rural geographic regions, across occupational of SES categories). The rationale for studying associations at state-level also needs proper justification. So current justification here needs to be revised. The authors may also want to have a look at these published papers to clarify their justification for this study:

1. <https://pdfs.semanticscholar.org/870c/449e35399633710a9ad4b223e46947102af1.pdf?_ga=2.118417226.2146522655.1614069121-810001830.1607255405>
2. <https://link.springer.com/article/10.1007/s11356-019-07341-x>
3. <https://link.springer.com/article/10.1007/s11356-020-10107-5>

**Authors:** Thank again this reviewer for giving us an opportunity to improve the revised manuscript. It is true that the level of implementation may vary by geographic region and GATS does not measure level of implementation. So according to the suggestion of this reviewer, we emphasis on the correlates of smoke-free policies and demonstrated the variation therein regions (between urban/rural geographic regions, across occupational of SES categories) and already the included the revised manuscript.

**Reviewer#1-11**: **In Method, Page 5, section 2.2**: Why only knowledge about “children’s illness” would be included as an independent variable? SHS exposure causes illness among all.

**Authors:** Thank again this reviewer for giving us an opportunity to improve the revised manuscript. We not consider other knowledge about SHS exposure causes illness to avoid multi-collinearity problem.

**Reviewer#1-12**: **In Method, Page 5, section 2.2**: Authors could provide more information about independent variables and groupings. The authors just mention ‘**household assets**’ but how SES variable was derived from that? Perhaps a supplementary table with variable definitions could be useful.

**Authors**: Thanks this reviewer for asking this question. We are totally agreed this reviewer. We have already added the information about independent variables along with their groupings of each variable which is presented a supplementary table in the revised manuscript as follows:

Table. Descriptions of the independent variables along with their categorizations

| Factors | Descriptions | Categorizations |
| --- | --- | --- |
| Smoking status | Smoking status | 1= Smoker, 2= Non-smoker |
| Age (years) | Age of the respondents in years | 1=15 to 30 years, 2= 31 to 45 years, 3= 46 to 60 years, 4= Up to 60 years |
| Gender | Gender of respondents | 1= Male, 2= Female |
| Marital status | Marital status of respondents | 1= single, 2= Married, 3=Separated/others |
| Religion | Religion | 1=Hindu,2=Muslim,3=Christian,4= Others |
| Place of residence | Place of residence | 1=Urban,2=Rural |
| Geographical regions | Respondent’s geographical regions | 1=North, 2= Central, 3=East, 4=North-east, 5=West, 6=South |
| Education | Education status of respondent’s | 1=No education, 2=Up to primary, 3=Up to secondary, 4=More than secondary |
| Occupation | Occupation status of the respondents | 1=Govt./Non-Govt. employee, 2= Daily wise/causal laborer, 3= Self-employed, 4=Homemaker, 5= Student, 6= Others |
| Household assets | Household assets of the respondents | 1=Poorest, 2= Poorer, 3= Middle, 4= Richer, 5= Richest |
| Knowledgeable of the children | Knowledgeable of the children illness | 1= No, 2= Yes |

**[Revised Manuscript Page #17, Supplementation]**

The household assets construction procedure is already added in the revised manuscript as follows:

*“In case of household assets, the following indicators were used: whether the household or person living in the household enjoys the facility of i) electricity, ii) electric fan, iii) air conditioner, iv) refrigerator, v) washing machine, vi) radio, vii) television, viii) computer/laptop, ix) internet connection, x) moped/scooter xi) car, xii) flush toilet, xiii) fixed telephone, xiv) cell telephone. Principal component analysis (PCA) had been conducted and this variable was divided into five categories (poorest, poorer, medium, richer, and richest) on the basis of the quintiles of the first PCA scores.”*

**[Revised Manuscript Page #5, Section #2, Subsection# 2.3]**

**Reviewer#1-13**: Section 2.3 (Data analysis)--The authors used logistic regression to study the correlates of living in smoke-free homes and working in smoke-free workplaces. **Some language edits are required here for clarity**. Did the authors perform any multi-collinearity diagnostics? It should be reported.

**Authors**: Thanks this reviewer for asking this question. At the initial stage we checked the existence of multi-collinearity problem by considering the value of standard error (SE) but didn’t include it in the manuscript. We are extremely sorry for that. Now, we have fixed it and update the revised manuscript. Thanks again.

**[Revised Manuscript Page #5, Section #2, Subsection# 2.4]**

**Reviewer#1-14**: Section 2.3 (Data analysis)--The analysis for state-level within and between analyses needs further description for clarity. This seems relevant because there is no description of how the “State” variable was treated in the analysis. From the results presented (**Table 3 and Correlation matrix**), it seems only overall National level estimates have been used and presented.

**Authors**: Thanks this reviewer for asking this question. We collected the estimated proportion of smoke-free policies and secondhand smoker’s exposure at both home and workplace from the GATS 2 report for each state to measure the correlation between the state-level proportion of smoke-free policies and secondhand smoker’s exposure at both home and workplace.

**[Revised Manuscript Page #5, Section #2, Subsection# 2.3]**

**Reviewer#1-15**: **Results,** in tables 1 and 2 the authors could club the other three categories for both outcomes unless they are going into their details. In Table 3, why are gender (for SF at home) and age groups (for SF at workplace) missing as In Table 1 gender is significantly associated with the outcome and in Table 2, age group is also significantly associated (p<0.05). Authors should check this.

**Authors**: Thanks this reviewer for asking this question. We deeply apologize for such mistakes. There was some misinterpretation in that table. We already fixed Tables in the revised manuscript. Thanks again for giving us an opportunity to improve the revised manuscript.

**Reviewer#1-16**: **Results,** Ok. At the end of results section and after seeing the correlation matrix, one realizes that it is not the “different settings of second hand smokers” but it is SHS exposure (at home, workplace and public places). This needs to be clarified in the methods and also in the abstract. “Different settings of secondhand smokers” is confusing. Again, when SHS exposure at public places has not been discussed throughout the paper, what is the rationale for bringing that in the correlation matrix?

**Authors**: Thanks this reviewer for asking this question. We have modified the word second hand smokers (SHS) to SHS exposure. The SHS exposure at public places was only depicted in the correlation matrix. We are totally agreed with your justification of being irrational for bringing that point and omitted this section from the correlation matrix. Thanks again for giving us an opportunity to improve the revised manuscript.

**Reviewer#1-17**: **Discussion,** Page 7:-First line of discussion–Non-smoker participants was more likely (NOT priorities)….

**Author:** Thanks this reviewer for asking this question. We are already modified the first line in the revised manuscript. Thanks again.

**Reviewer#1-17**: **Discussion,** Page 7:-Well, most of the results and discussion deals with for e.g. females vs. males are more likely to live in smoke-free homes/workplaces and similar findings. What is not discussed in Discussion section, is the policy implications of the findings. In India, the smoke-free legislation is a national law (under COTPA 2003) and probably the level of implementation varies across geographic areas (rural/urban, national regions, states). So how are the findings specifically relevant to the national policy scenario? That needs to be discussed in detail.

**Author:** Thanks this reviewer for asking this question. We are already modified the whole discussion section the revised manuscript. Thanks again.

**[Revised Manuscript Page #7, 8, 9, Section #4]**

Dear Reviewer #1

Thank you very much for your kind guidance and support in helping the authors improve the manuscript.

We are truly grateful for your time and support.

Kind Regards,

The Authors of the manuscript.

**Reviewer #2:**

 This study used data from the 2016/17 wave of the GATS conducted in India to analyse the prevalence and determinants of smoke-free policies in the home and in the workplace among Indian people aged 15+. Towards this research aim, this study did a great job to perform the analyses rigorously and interpret the results in great detail. However, it is less clear what the second aim -- assessing the state-level within and between relationships among the nature of smoke free policies prevailing at different settings where secondhand smoking occurs -- is about, and why it is important to examine this aim.

**Reviewer#2-1**: Title: 2017 should be changed to 2016/2017 because the GATS2-India was conducted in the period between August 2017 and February 2017.

**Authors:** Thank you for your suggestions. The manuscript has been revised and modified by considering your instruction.

**[Revised Manuscript Page #1, 2; Title of the manuscript]**

**Reviewer#2-2**: Abstract lines 5-6: Why is it necessary to examine the correlation between smoke-free policies in the home and smoke-free policies in the workplace?

**Authors:** Thank you very much for asking this question. We are totally agreed with this reviewer. We update the objectives in the revised manuscript as follows:

**Old objective:** To determine the correlation between smoke-free policies in the home and workplace and determine the association between and within smoke-free policies at different setting where secondhand smoking occurs

**New Objective:** To determine the correlates of smoke-free policies in the home and workplace along with assessing the relationship between smoke-free policies and secondhand smoker’s exposure.

**[Revised Manuscript Page #2; Abstract: Line#: 1-3]**

**Reviewer#2-3**: There is a lack of explanation about the **secondhand smoke measure**. In the abstract (page 2, lines 6-7), Introduction section (page 4, lines 8-9), Statistical Analysis section (page 5, lines 6-8 of the second paragraph), and Results section (page 7, the second paragraph), the authors talked **about the associations between smoke-free policies and different settings of secondhand smoke**. However, there is no explanation about the definition of secondhand smoke, how many settings were examined, and how each setting of secondhand smoke was measured. Also, is secondhand smoke an outcome variable or independent variable? **This information needs to be described in the Methods section**.

**Authors:** Thank you very much for asking this question. Actually, we do not consider secondhand smoker in our main analysis. We collected the estimated proportion of smoke-free policies and secondhand smoker’s exposure at both home and workplace from the GATS 2 report for each state to measure the correlation between the state-level proportion of smoke-free policies and secondhand smoker’s exposure at both home and workplace. We are already updated it’s in the revised manuscript.

**[Revised Manuscript Page #5; Section#2, Subsection# 2.4]**

**Reviewer#2-4**: Introduction, 1st paragraph, lines 9-10: “Among the total tobacco consumers, 42.4% are male and 14.2% are female.” This sentence does not look right because the male tobacco

 consumers (42.4%) and female consumers (14.2%) should add up to total tobacco consumers (100%). Does “42.2%” refer to “the prevalence of tobacco use”?

**Authors:** Thank you very much for asking this question. Actually, we do not show the proportion/percentages of male and female tobacco consumers. So, it is not mandatory to reach 100% (their sum). Actually, we show the prevalence of male and female tobacco consumer. In the previous manuscript, we have some mistakes to sentence structure. Now, we fixed it and updated the revised manuscript. Thanks again.

**Old:** Among the total tobacco consumers, 42.4% are male and 14.2% are female

**New:** The prevalence of male and female tobacco consumers is 42.4% and 14.2%.

**[Revised Manuscript Page #3; Section#1]**

**Reviewer#2-5**: Introduction, 2^nd^ paragraph, lines 10-12: For the sentence which begins with “Some previous country or community, can you cite some references to justify this sentence?

**Authors:** Thank you very much for asking this question. We are extremely sorry for our mistake. Now, we added some references in the revised manuscript. Thanks again.

**[Revised Manuscript Page #3-4; Section#1]**

**Reviewer#2-6**: Sample size: Page 4, lines 11-12 of the second paragraph: “After removing respondents with missing values” Please explain the reason for missing values. For example, how many respondents were excluded due to missing values for the outcome variable, and how many due to missing values for independent variables?

**Authors:** Thank you very much for asking this question. In GATS 2, a total of 74,037 individual interviews were completed with an overall response rate of 92.9%. Among the respondent’s, 7 were refused and 244 responded with don’t know about smoke-free policies at home. After excluding these respondents along with 2740 (missing, refused, don’t know etc.) in the selected independent variables, 71, 046 respondents were selected for final analysis in case of smoke-free policies at home.

The analysis of smoking policies at workplace was restricted to the GATS2 respondents (18258) who were working indoors or both indoors and outdoors but outside their home (adults). Among them, 7 respondents are refused and 374 respondent’s responses don’t know about the work place policies. After excluding these respondent’s (381) and 393 missing, refused, don’t know etc.) in the selected independent variables, 17, 484 respondents were selected for final analysis for smoke-free policies at work place.

**Reviewer#2-7**: The paragraph about the independent variables needs to be substantially expanded by providing detailed definition of each independent variable. For example, tobacco smoking status was classified into smokers, and non-smokers. How do you define “smokers” and “non-smokers defined”?

**Authors:** Thank you very much for asking this question. In Gats 2016-2017 questionnaire, there is a question about tobacco smoking: Does the person currently smoke tobacco including bidhis, cigarattes, hukkah, cigars etc.? (i) Yes, (ii) No, (iii) Don’t know, and (iv) Refused. The frequency table as follows:

|  | Frequency | % |
| --- | --- | --- |
| Yes | 11767 | 15.9 |
| No | 62109 | 83.9 |
| Don’t know | 160 | 0.2 |
| Refused | 1 | 0.00 |

We omit the respondents who refused and replied with don’t know. Thanks again.

**Reviewer#2-8**: More explanation is needed about what analyses utilized the chi-square test (which was stated in the “Statistical Analysis” paragraph). Also, please clarify meaning of “a **feature selection method** for further analysis”.

**Authors:** Thank you very much for asking this question. We are extremely sorry for mistake. We are already fixed it in the revised manuscript. Thanks.

**Reviewer#2-9.** The classification of the two outcome variables – smoke-free policy at home, and smoke-free policy in the workplace – seems confusing and inconsistent. First, In the “Variable of the study” paragraph, the smoke-free policy at home variable was classified as a binary variable: “living in a complete smoke-free home” vs. “not living in a complete smoke-free home“. Second, in the Results section, this variable was classified into a 3-category variable: “complete smoke-free home”, “partial smoke-free home”, and “no rules about smoking”. Third, in Table 1, this variable was classified into a 4-cateogy variable: “never allowed”, “not allowed, but exception”, “no rules”, and “allowed”. It is not clear whether the chi-square test was conducted using the first classification, or the second classification, or the third classification. Similar problem also exists for the smoke-free policy in the workplace variable.

**Authors:** Thank you very much for asking this question. In our main dataset, smoke-free policies at home and workplace consist of 4 categories. To assess the association between smoke-free policies and independent variables using Chi-square test, we consider four categories. But to determine the correlates of complete smoke-free policies at both home and workplace converted into binary. It is a common case in public health.

**Reviewer#2-10.** Page 6, lines 9-10 of the second paragraph: To justify the claim that “Participants who lived in South region … had the highest tendency to live …..compared to other regions.”, the multivariate logistic regression model needs to be revised using “south” **as the reference group**.

**Authors:** Thank you very much for asking this question. We are already done in the revised manuscript.
 
**Reviewer#2-10.** The first sentence in the Conclusion section indicates that “54.4% had”, while page 6, line 1 says that 50.4% had complete smoke-free policies at home. Which one is correct?

**Authors:** Thank you very much for asking this question. This was the type of mistake. Actually, 54.4% had complete smoke-free polices at home correct not 50.4%. We are already fixed it and update the revised manuscript. Thanks again.

**Reviewer#2-10.** Tables 1-2 need to show total sample size, change “tobacco polices” to “smoke-free policies” in the title, and add footnote to explain what the superscripts “1”, “(a)”, and “(b)” mean.

**Authors:** Thank you very much for asking this question. We are already fixed it and update the revised manuscript. Thanks again.

**Reviewer#2-10.** Table 3 needs to add outcome variable information in the title, and change the term “odds ration” to “adjusted odds ratio”.

**Authors:** Thank you very much for asking this question. We are already fixed it and update the revised manuscript. Thanks again.

Dear Reviewer #2

Thank you very much for your kind guidance and support in helping the authors improve the manuscript.

We are truly grateful for your time and support.

Kind Regards,

The Authors of the manuscript.
