# Supplementary material for: Smoke-free status of homes and workplaces among Indian people: Evidence from Global Adult Tobacco SurveyData-2016/2017
Source: PLoS One. 2023 Feb 23;18(2):e0282138. doi: 10.1371/journal.pone.0282138 (PMC9949653; doi:10.1371/journal.pone.0282138)
Supplement: Attachment — Submitted filename: Rebuttal-Ver1.docx [file pone.0282138.s004.docx]

**Authors’ Response to Reviewers**

Manuscript ID: PONE-D-20-41034R1

**Title: Smoke-free policies in the home and in the workplace among Indian people: Evidence from Global Adult Tobacco Survey Data-2016/2017**

Dear Respected Editor-in-Chief,

First of all, we thank the reviewer and the editor for their useful suggestions for improving the manuscript. We have now revised the manuscript accordingly. Thank you once again and looking forward to the final acceptance in your esteemed journal.

**Reviewer (R1):**

The authors have addressed a number of comments that were raised. The manuscript still requires significant copy-editing. There are some concerns regarding the terminologies used. The authors should ensure correct terms are used which are consistent with previous tobacco literature. There are some minor recommendations that I believe would improve the quality of the paper.

**Abstract:**

**R1-1:** Aim-edit “**secondhand smoker’s exposure**” to “**secondhand smoke exposure**”. Please ensure this changed is reflected throughout the manuscript.

**Authors:** Thank you for your suggestions. We are already replaced the word “**secondhand smoker’s exposure**” to “**secondhand smoke exposure**” throughout the manuscript and update the revised manuscript.

**[Revised Manuscript Page #2; Aims (in Abstract)]**

**R1-2:** Methods-edit “**proportion of secondhand smoker’s exposure**” to “**proportion of participants exposed to secondhand smoke**”.

**Authors:** Thank you for your suggestions and giving us an opportunity to improve the revised manuscript. We are already replaced the word “**proportion of secondhand smoker’s exposure**” to “**proportion of participants exposed to secondhand smoke**” throughout the manuscript and update the revised manuscript.

**[Revised Manuscript Page #2; Methods (in Abstract)]**

**R1-3:** Results-edit “Results found that” to “We found that” edit “**secondhand smoker’s exposure**” to “**secondhand smoke exposure**”.

**Authors:** Thank you for your suggestions. We are already replaced the word “**secondhand smoker’s exposure**” to “**secondhand smoke exposure**” throughout the manuscript and update the revised manuscript.

**[Revised Manuscript Page #2; Results (in Abstract)]**

**R1-4:** Conclusion-smoke free policies are already in place in India. Maybe authors could emphasize “**comprehensive smoke free policies without any exceptions**” and their “enhanced monitoring.” This could be a part of discussion and conclusion but authors should note - Penalty for violation of smoke-free legislation in public places is currently only INR 200 (approx. USD 2.7) which needs to be increased considerably taking into account inflation and rise in purchasing power/incomes. The proposed COTPA Amendment Bill (2020) proposes to increase this penalty to INR 2000 but this Bill has not yet been tabled in the Parliament.

**Authors:** Thank you for your suggestions. The manuscript has been revised and modified by considering your instruction.

**Introduction**

**R1-5:** First para-6^th^ line from bottom – remove “of them”

**Authors:** Thank you for your suggestions. The manuscript has been revised and modified by considering your instruction.

**[Revised Manuscript Page #3; Section#1, First para-4-5th line from bottom]**

**R1-6:** First para-5th line from bottom – “…tendency to smoke themselves but exposure to SHS…” can be replaced by “…tendency to smoke, exposure to SHS….”

**Authors:** Thank you for your suggestions. The manuscript has been revised and modified by considering your instruction.

**[Revised Manuscript Page #3; Section#1, First para-5th line from bottom]**
**R1-7:** Second para – last sentence – “**a progress of smoke-free policies**” should be replaced by “**progress in smoke-free policies**”. “Government still couldn’t reach its ultimate goal” could be replaced by “the policies are still not comprehensive”

**Authors:** Thank you for your suggestions. The manuscript has been revised and modified by considering your instruction.

**[Revised Manuscript Page #3; Section#1, 2^nd^ Para-Last sentence]**

**R1-8:** Third para – “**A number of countries or community-based Researches**” could simply be replaced by “**Previous research has**”. And “**secondhand smoker’s exposure**” should be replaced at all places by “**exposure to secondhand smoke**”.

**Authors:** Thank you for your suggestions. The manuscript has been revised and modified by considering your instruction.

**[Revised Manuscript Page #3-4; Section#1, 3^rd^ Para]**

**R1-9: Variables of the study** – I think the **household asset** variable should be termed as the “**wealth quintile**” or “**asset quintile**”. This is typically the term used in similar research papers.

**Authors:** Thank you for your suggestions. We are already replaced the word “**household asset**” to “**wealth quintile**” throughout the manuscript and update the revised manuscript.

**[Revised Manuscript Page #5; Section#2, subsection#2.3, 2^nd^ Para]**

**R1-10:** Similarly, there is a problem with terminologies here “**proportion of smoke-free policies** and **secondhand smoker’s exposure at both home and workplace**”. This could be replaced by “**proportion of participants living in smoke-free homes**; **working in smoke-free workplaces** and **proportion of participants exposed to secondhand smoke**.” The authors should take care of these terminologies throughout the paper. The authors may also want to clarify here whether the **proportion of participants exposed to SHS** was restricted to non-smokers only.

**Authors:** Thank you for your suggestions. The manuscript has been revised and modified by considering your instruction.

**[Revised Manuscript Page #2; Section#2, subsection#2.3, last Para]**

**R1-11:** The **multicollinearity problem**” could be replaced by just “**multicollinearity**”. The authors should run their regression models with ‘collin’ command (if using STATA) after running each logistic model and look for higher VIF values. While there is no rule of thumb for this, they could report that multicollinearity was assessed and all **VIF values were less than 10**, indicating **no significant multicollinearity between explanatory variables**. Multicollinearity between variables can lead to high SEs of coefficients and there are less chances of them being significant. Hence, only of such variables should be retained in the model.

Apologies for not pointing this out earlier but in the statistical analysis section, the authors should report about using survey weights “**svyset**” to account for the complex multistaged GATS survey design.

**Authors:** Thank you for your suggestions. Yes, we are totally agreed with this reviewer. We are already run our logistic model with “**collin**” command (using STATA) after running logistic model and look VIF values for checking multicollinearity between independent variables. We observed that all VIF values were less than 10, indicating no significant multicollinearity between explanatory variables. Now, we have revised the Table and also updated the revised manuscript. Thanks again.

*Table 3. Multivariate logistic regression results*

| *Variable* | | *Home Place* | | | *Work Place* | | |
| --- | --- | --- | --- | --- | --- | --- | --- |
|  |  | *p-value* | *AOR (95% CI of AOR)* | *VIF* | *p-value* | *AOR (95% CI of AOR)* | *VIF* |
| ***Tobacco Smoking*** | *Smoker®* |  |  | *1.05* |  |  | *1.11* |
| ***status*** | *Non-smoker* | *<0.001* | *3.285 (3.001,3.595)* |  | *<0.001* | *1.627 (1.404, 1.886)* |  |
| ***Age group*** | *15-30* |  | *-* |  |  | *-* |  |
|  | *31-45* |  | *-* |  |  | *-* |  |
|  | *46-60* |  | *-* |  |  | *-* |  |
|  | *60+* |  | *-* |  |  | *-* |  |
| ***Gender*** | *Male®* |  | *-* |  |  |  | *1.11* |
|  | *Female* |  | *-* |  | *0.048* | *1.229 (1.002, 1.510)* |  |
| ***Marital status*** | *Single®* |  |  | *1.13* |  |  | *1.06* |
|  | *Married* | *0.420* | *0.961 (0.874, 1.058)* |  | *0.604* | *0.954 (0.797, 1.141)* |  |
|  | *Separated/others* | *0.274* | *1.081 (0.940, 1.242)* |  | *0.934* | *0.984 (0.677, 1.431)* |  |
| ***Religion*** | *Hindhu®* |  |  | *1.01* |  |  | *1.01* |
|  | *Muslim* | *0.074* | *0.885 (0.774, 1.012)* |  | *0.510* | *1.069 (0.876, 1.305)* |  |
|  | *Christian* | *0.901* | *0.987 (0.805, 1.211)* |  | *0.084* | *0.710 (0.482, 1.047)* |  |
|  | *Others* | *<0.001* | *1.939 (1.650, 2.278)* |  | *0.589* | *1.099 (0.778, 1.554)* |  |
| ***Place of residence*** | *Urban®* |  |  | *1.21* |  |  | *1.19* |
|  | *Rural* | *<0.001* | *1.376 (1.253, 1.511)* |  | *0.015* | *1.245 (1.044, 1.484)* |  |
| ***Region*** | *North®* |  |  | *1.01* |  |  | *1.01* |
|  | *Central* | *0.434* | *0.952 (0.840, 1.077)* |  | *0.170* | *0.866 (0.706, 1.064)* |  |
|  | *East* | *<0.001* | *1.249 (1.107, 1.408)* |  | *0.003* | *1.424 (1.132, 1.791)* |  |
|  | *North-east* | *0.001* | *1.258 (1.095, 1.445)* |  | *0.162* | *1.181 (0.935, 1.491)* |  |
|  | *West* | *<0.001* | *1.637 (1.423, 1.885)* |  | *0.001* | *1.671 (1.247, 2.239)* |  |
|  | *South* | *<0.001* | *5.339 (4.764, 5.985)* |  | *<0.001* | *1.836 (1.476, 2.285)* |  |
| ***Education*** | *Noeducation®* |  |  | *1.44* |  |  | *1.46* |
|  | *Uptoprimary* | *0.004* | *1.127 (1.038, 1.222)* |  | *0.001* | *1.356 (1.124, 1.636)* |  |
|  | *Upto secondary* | *<0.001* | *1.401 (1.286, 1.527)* |  | *<0.001* | *1.831 (.501, 2.235)* |  |
|  | *Morethan* | *<0.001* | *1.879 (1.695, 2.083)* |  | *<0.001* | *2.317 (1.834, 2.928)* |  |
|  | *secondary* |  |  |  |  |  |  |
| ***Occupation*** | *Govt./Non-Govt.* |  |  | *1.07* |  |  | *1.10* |
|  | *employee®* |  |  |  |  |  |  |
|  | *Dailywise/causal*  *laborer* | *0.008* | *0.853 (0.758, 0.959)* |  | *<0.001* | *0.468 (0.381, 0.574)* |  |
|  | *Selfemployed* | *0.094* | *0.913 (0.821, 1.016)* |  | *<0.001* | *0.559 (0.458, 0.684)* |  |
|  | *Homemaker* | *0.530* | *1.047 (0.908, 1.206)* |  | *0.001* | *2.064 (1.327, 3.208)* |  |
|  | *Student* | *0.001* | *0.821 (0.733, 0.919)* |  | *0.001* | *0.495 (0.329, 0.743)* |  |
|  | *Others* | *0.025* | *0.856 (0.746, 0.981)* |  | *0.095* | *0.629 (0.366, 1.083)* |  |
| ***Wealth quintile*** | *Q_1_(poorest)®* |  |  | *1.41* |  |  | *1.47* |
|  | *Q_2_* | *0.623* | *0.977 (0.889, 1.072)* |  | *0.121* | *1.173 (0.959, 1.435)* |  |
|  | *Q_3_* | *0.170* | *1.076 (0.969, 1.195)* |  | *0.051* | *1.248 (0.998, 1.559)* |  |
|  | *Q_4_* | *0.018* | *1.176 (1.028, 1.346)* |  | *0.016* | *1.378 (1.061, 1.789)* |  |
|  | *Q_5_(richest)* | *0.108* | *1.103 (0.979, 1.243)* |  | *0.007* | *1.416 (1.101, 1.820)* |  |
| ***Otherpeople’s*** | *No®* |  |  | *1.02* |  |  |  |
| ***Smokingcauseserious***  ***Illnessinchildren*** | *Yes* | *0.003* | *1.219 (1.068, 1.391)* |  |  | *-* |  |

*- indicates that the variable is insignificant in bivariate analysis*

**[Revised Manuscript Page #15; Table #3]**

In case of sampling weight, as per your suggestions, Sampling weights were applied and weighted estimates were calculated to account for the complex study design. Clustering and stratification was also accounted for by using **svyset** command in STATA. The following variables were used to apply clustering and stratification: gatscluster, gatsstrata and gatsweight. Now, we have revised and updated the statistical analysis section in the revised manuscript as follows:

“*Descriptive statistics was used to explore the scenario of the smoke-free policies in the home and in the workplace as well as the basic characteristics of the respondents. A Chi-square test was used to access the association between dependent and independent variables. Logistic regression analysis was used to determine the significant correlates of smoke-free policies at home and in the workplace* ***and estimated adjusted odds ratio (AOR) along with 95% confidence interval of AOR. In this study, the multicollinearity among different independent variables was checked using variance inflation factor (VIF) values. If the all VIF values were less than 10, indicating there was no significant multicollinearity between independent variables [23]. Sampling weights were applied and weighted estimates were calculated to account for the complex study design. Clustering and stratification was also accounted for by using svyset command in STATA. The following variables were used to apply clustering and stratification: gatscluster, gatsstrata and gatsweight.*** *Pearson correlation was also used to explore the relationship between smoke-free policies and the proportion of secondhand smoke exposure at both home and workplace.”*

**[Revised Manuscript Page #5-6; section#2, subsection#2.4]**

**R1-12: In Results,** Just want to point out here to the authors that SHS typically stands for “**Secondhand Smoke**” in tobacco literature and not “**Secondhand Smoker**’s”. This should be corrected throughout the paper and also in tables and their legends. The correct term should be “Secondhand smoke exposure” or SHS exposure.

**Authors:** Thank you for your suggestions and giving us an opportunity to improve the revised manuscript. We have already revised throughout the paper along with tables and their legends and updated the revised manuscript. Thanks again.

**R1-13: In Conclusion,** this needs to be strengthened. As mentioned earlier, the authors studied complete smoke-free policy vs. no/partial smoke-free policy both at home and at workplace. Hence, it is the complete or comprehensive smoke-free policy (without any exceptions [designated smoking rooms] such as at airports, restaurants or hotels) that needs to be enforced (not partial). There could be enhanced monitoring of implementation of such comprehensive smoke-free policies and fines/penalties should be substantially increased to discourage smoking in public places (and protect the non-smokers from SHS).

**Authors:** Thank you for your suggestions. The manuscript has been revised and modified by considering your instruction.

**Reviewer (R2):**

This revised paper still needs a substantial revision and editing.

**R2-1:** There are published papers which used the same GATS-India-II data to examine the prevalence and correlates of SHS exposure in India (**Tripathy 20201, 2020b**). There is a need to discuss how this study differs from the published studies.

**Authors:** Thank you for asking this question and giving us an opportunity to improve the revised manuscript. The pattern of our current study is similar with **Tripathy (20201, 2020b)**. But our objectives are totally differed with **Tripathy (20201, 2020b)**. These are as follows:

The main aim of this study was to determine the prevalence and correlates of **smoke-free policies in the home and workplace.** We have also investigated **the state level relationship** between smoke-free policies and secondhand smoke exposure. Whereas, **Tripathy (2020) examined the relationship between** smoke-free workplaces and smoke-free homes in India. He also examined the state level relationship between Smoke-free workplaces and smoke-free homes. **Tripathy (2021)** was examined the prevalence and correlates of **SHS exposure** at home and public place in India.

**R2-1a:** I found some question about sample size. The study by **Tripathy (2020)** used all observations in the GATS-India-II (N=74,037) and identified **64,538** non-smokers and 9,499 current smokers (including 7,647 daily smokers and 1,852 less-than-daily smokers). After deleting missing values, this study included 71,046 for the analysis of smoke-free policies at home. However, there are 11,321 current smokers according to Table 1. Why the sample size of current smokers is larger than the sample size (**11,321 vs. 9,499**) in the study by **Tripathy (2020)** even though the total sample size is smaller (**71,046 vs. 74,037**)? I suspect that this study might use a different definition of current smokers.

**Authors:** Thank you for asking this question and giving us an opportunity to improve the revised manuscript. In order to classify the current tobacco smokers Tripathy (2020) considered the variable as **B01: Do you currently smoke tobacco on a daily basis, less than daily, or not at all?** Whereas, we have considered the variables as **HH4E01: Does this person currently smoke tobacco, including bidis, cigarettes, hukkah, cigars, and pipes**? For this reason, the numbers of current tobacco smokers are differed with Tripathy (2020). We have presented the frequency of both considering variables (B01 vs. HH4E01)**.**

| **Before Cleaning (N=74,037)** | **After Cleaning (N=71,046)** |
| --- | --- |
|  |  |

**R2-1b:** I also found some inconsistent findings. **Tripathy (2020) reported that exposure to SHS in the home was 29.2% among all respondents** and **it was higher among females (30.4%)** compared to males (28.1%); and that exposure to SHS in workplaces was 30% overall and it was higher among males (32.5%) compared to females (17.8%). However, this study yielded opposite results compared with **Tripathy (2020)**.

**Authors:** Thank you for your suggestions. In our current study, we have presented the prevalence of smoke-free polices at home and workplace whereas, Tripathy (2020 Tripathy (2020) reported that exposure to SHS in the home and public place. We have carefully checked our results section; there is no any prevalence of exposure to SHS in the home and public place. We have only addressed some statistics from GATS 2 report in the introduction section as follows:

*“38.7% (38.1% male vs. 39.3% female) of exposure to secondhand smoke (SHS) takes place at home, 30.2% (32.7% male vs. 17.9% female) at workplace and 5.3% (8.1% male vs. 2.4% female) at government office, 5.6% (6.8% male vs. 4.4% female) at health care facilities, 7.4% (13.0% male vs. 1.6% female) at restaurants, and 13.3% (16.6% male vs. 9.9% female)”.*

Thanks again for your understanding.

**Reference**

Tripathy JP. Secondhand smoke exposure at home and public places among smokers and non-smokers in India: findings from the Global Adult Tobacco Survey 2016-17. Environ Sci Pollut Res Int. 2020a; 27(6):6033-6041. doi: 10.1007/s11356-019-07341-x.

Tripathy JP. Smoke-free workplaces are associated with smoke-free homes in India: evidence for action. Environ Sci Pollut Res Int. 2020b;27(33):41405-41414. doi: 10.1007/s11356-020-10107-5.

**R2-2:** (Page 2, abstract) the main aim of this study is to determine the correlates of smoke-free policies in the home and in the workplace. Before addressing the correlates, **it is necessary to examine the prevalence of smoke-free policies in the home and in the workplace**.

**Authors:** Thank you for your suggestions and giving us an opportunity to improve the revised manuscript. Yes, we are totally agreed with this reviewer. Now, we have added a table as prevalence of prevalence of smoke-free policies in the home and in the workplace in the revised manuscript and updated the revised manuscript as follows:

Table 1. Prevalence of smoke-free policies in the home and in the workplace

| Characteristics | Smoke-free policies in | | | | | |
| --- | --- | --- | --- | --- | --- | --- |
|  | Home | | | Workplace | | |
|  | Yes, % | No, % | p-value | Yes, % | No, % | p-value^1^ |
| Overall | 62.8 | 37.2 |  | 51.7 | 48.3 |  |
| **Tobacco Smoking status** |  |  | <0.001 |  |  | <0.001 |
| Smoker | 28.5 | 71.5 |  | 48.5 | 51.5 |  |
| Non-smoker | 56.1 | 43.9 |  | 66.9 | 33.1 |  |
| **Age group** |  |  | 0.944 |  |  | 0.676 |
| 15-30 | 51.8 | 48.2 |  | 63.6 | 36.4 |  |
| 31-45 | 51.8 | 48.2 |  | 61.8 | 38.2 |  |
| 46-60 | 51.3 | 48.7 |  | 62.7 | 37.3 |  |
| 60+ | 51.5 | 48.5 |  | 63.2 | 36.8 |  |
| **Gender** |  |  | 0.205 |  |  | <0.001 |
| Male | 52.2 | 47.8 |  | 61.6 | 38.4 |  |
| Female | 51.1 | 48.9 |  | 68.9 | 31.1 |  |
| **Religion** |  |  | <0.001 |  |  | 0.0086 |
| Hindhu | 51.9 | 48.1 |  | 63.5 | 36.5 |  |
| Muslim | 45.4 | 54.6 |  | 57.1 | 42.9 |  |
| Christian | 65.4 | 34.6 |  | 64.6 | 35.4 |  |
| Others | 35.0 | 65.0 |  | 68.5 | 31.5 |  |
| **Marital status** |  |  | <0.001 |  |  | <0.001 |
| Single | 56.0 | 44.0 |  | 69.0 | 31.0 |  |
| Married | 50.2 | 49.8 |  | 60.9 | 39.1 |  |
| Separated/others | 52.6 | 47.4 |  | 58.9 | 41.1 |  |
| **Place of residence** |  |  | <0.001 |  |  | <0.001 |
| Urban | 46.1 | 53.9 |  | 56.0 | 44.0 |  |
| Rural | 62.1 | 37.9 |  | 70.8 | 29.2 |  |
| **Region** |  |  | <0.001 |  |  | <0.001 |
| North | 56.3 | 53.7 |  | 62.3 | 37.7 |  |
| Central | 38.3 | 61.7 |  | 52.7 | 47.3 |  |
| East | 43.9 | 56.1 |  | 58.1 | 41.9 |  |
| North-east | 43.3 | 56.7 |  | 55.9 | 44.1 |  |
| West | 56.6 | 43.4 |  | 74.7 | 25.3 |  |
| South | 77.9 | 22.1 |  | 72.2 | 27.8 |  |
| **Education** |  |  | <0.001 |  |  | <0.001 |
| No education | 41.5 | 58.5 |  | 41.5 | 58.5 |  |
| Up to primary | 46.4 | 53.6 |  | 50.8 | 49.2 |  |
| Up to secondary | 53.6 | 46.4 |  | 64.8 | 35.2 |  |
| More than secondary | 65.1 | 34.9 |  | 76.3 | 23.7 |  |
| **Occupation** |  |  | <0.001 |  |  | <0.001 |
| Govt./ Non-Govt. employee | 60.7 | 39.3 |  | 75.5 | 24.5 |  |
| Daily wise/ causal labourer | 46.8 | 53.2 |  | 46.6 | 53.4 |  |
| Self employed | 49.1 | 50.9 |  | 58.2 | 41.8 |  |
| Homemaker | 59.6 | 40.4 |  | 84.0 | 16.0 |  |
| Student | 50.5 | 49.5 |  | 52.8 | 47.2 |  |
| Others ^(a)^ | 50.1 | 49.9 |  | 58.0 | 42.0 |  |
| **Household asset^(b)^** |  |  | <0.001 |  |  | <0.001 |
| Q_1_ (poorest) | 39.1 | 60.9 |  | 46.1 | 53.9 |  |
| Q_2_ | 48.7 | 51.3 |  | 56.7 | 43.3 |  |
| Q_3_ | 54.5 | 45.5 |  | 62.1 | 37.9 |  |
| Q_4_ | 62.8 | 37.2 |  | 74.2 | 25.8 |  |
| Q_5_ (richest) | 59.6 | 40.4 |  | 72.5 | 27.5 |  |
| **Other people’s smoking cause serious illness in children** |  |  | <0.001 |  |  | 0.484 |
| Yes | 52.1 | 47.9 |  | 62.9 | 37.1 |  |
| No | 43.4 | 56.6 |  | 60.0 | 40.0 |  |

Q1: Poorest; Q2: Poorer; Q3: Middle; Q4: Richer; Q5: Richest.

**[Revised Manuscript Page #1; Table#2]**

**R2-3:** The second aim of this study is to assess the relationship between smoke-free policies and secondhand smoke (SHS) exposure. H**owever, there is a lack of explanation for why examining this aim is important**. Also, while the main aim of this study was analyzed using persons as the unit of the analysis, this aim was analyzed using states (30 states and 2 Union Territories) as the unit of the analysis. Moreover, while the main aim of this study was analyzed using the multivariable logistic regression model, this aim was analyzed using a simple Pearson’s correlation without adjusting for other confounding factors. If the authors really want to include this second aim in this paper, this aim should be examined in a much more rigorous way.

**Authors:** Thank you for your suggestions. We are totally agreed with you. In our current study, we do not adjust any confounding factor which was limitations of our study. We already address this issue in the limitations section in the revised manuscript. Thanks again.

**[Revised Manuscript Page #9; Section#4, subsection#4.1]**

**R2-3a:** First, the **definition of the SHS exposure variables, including what GATS questions were used to measure SHS exposure, should be explained clearly in the Methods section**.

**Authors:** Thank you for your suggestions. We are already add GATS questions to use to measure SHS exposure in the revised manuscript.

**[Revised Manuscript Page #5; Section#2, subsection#2.3]**

**R2-3b:** Second, a **multivariable regression model which controls for other confounding factor** should be used to analyze the association between smoke-free policies and SHS exposure.

**Authors:** Thank you for your suggestions. We are totally agreed with you. In our current study, we do not adjust any confounding factor which was limitations of our study. We already address this issue in the limitations section in the revised manuscript. Thanks again.

**[Revised Manuscript Page #9; Section#4, subsection#4.1]**

**R2-3c:** Third, justification is needed for why the analysis needs to be conducted at state-level rather than at person-level.

**Authors:** Thank you for your suggestions. The implementation of restrictions/rules about smoke-free policies varies state to state [Ali et al., 2020]. For this reason, we fixed our interest was to show the state relationship between the smoke-free policies and exposure to SHS at both home and workplace. Thanks again.

**R2-4:** (Page 2, abstract). **More specific results about the correlates of smoke-free policies need to be provide in the Results section**. For example, what are the prevalence estimates for smoke-free policies among all people aged 15+ in India? How does the prevalence differ by sub-groups stratified by each correlate? What are the key findings concerning the adjusted odds ratios for the associations between correlates and smoke-free policies?

**Authors:** Thank you for your suggestions. We have already updated it in the revised manuscript. Thanks again.

**R2-5:** (Page 2, abstract). The Conclusion section needs to be re-written to highlight the key findings.

**Authors:** Thank you for your suggestions. The manuscript has been revised and modified by considering your instruction.

**R2-6:** Page 3, 1st paragraph: “GATS-2 also reports that 38.7% … of exposure to secondhand smoke (SHS) takes place at home, 30.2% … at workplace and 23% … occurs at any public place”. Before this sentence, it is important to let the readers know **what was the prevalence of secondhand smoke exposure in India according to the GATS-2 data**. Also, does this sentence mean that among people who were exposed to secondhand smoke, 38.7% were exposed at home, 30.2% were exposed at workplace, and 23% were exposed at public place. These 3 percentages only add up to 91.9%. What is the source of secondhand smoke exposure for the remaining 8.1%?

**Authors:** Thanks again for giving us your suggestions to improve the revised manuscript. We have already fixed the above sentence and updated the revised manuscript as follows:

*“According to the GATS 2 (2016-2017) data, 38.7% (38.1% male vs. 39.3% female) of exposure to secondhand smoke (SHS) takes place at home, 30.2% (32.7% male vs. 17.9% female) at workplace and 5.3% (8.1% male vs. 2.4% female) at government office, 5.6% (6.8% male vs. 4.4% female) at health care facilities, 7.4% (13.0% male vs. 1.6% female) at restaurants, and 13.3% (16.6% male vs. 9.9% female) [6].”*

**[Revised Manuscript Page #3; Section#1, 1^st^ Para]**

**R2-7:** Page 3, 2nd paragraph “Although there is a progress of smoke-free policies … government still couldn’t reach its ultimate goal.” What is the ultimate goal of the National Tobacco Control Program launched in 2007-2008?

**Authors:** Thank you for your suggestions. We have already updated the above sentence as per guidance of Reviewer#1. Thanks again.

**R2-8:** Page 3, 3rd paragraph.
**R2-8a:** Delete the wording “To our best knowledge”.

**Authors:** Thank you for your suggestions. We have already removed the word “**To our best knowledge**” from our revised manuscript. Thanks again.

**R2-8b:** In the sentence “However, most of these previous studies have been focused on the prevalence and predictors of tobacco use [11, 18-20]”, the cited studies were not relevant because they are about secondhand smoke exposure rather than about tobacco use.

**Authors:** Thank you for your suggestions and giving us an opportunity to improve the revised manuscript. Yes, we are totally agreed with this reviewer. Now, we have fixed it and updated the revised manuscript. Thanks again.

**R2-8c:** In the sentence “A study also observed that a smoke-free workplace was … associated with SHS exposure at home [21] “, Ref #19 can also be cited in addition to Ref #21.

**Authors:** Thank you for your suggestions. As your suggestions, we have already cited Ref#19 instead of Ref#21 and updated the revised manuscript. Thanks again.

**R2-8d:** In the sentence “A number of countries or community … the presence of policies that restrict smoking in the workplace and home may vary by … status [19, 22]“, Ref #19 should not be cited because it is about secondhand exposure rather than about smoke-free policies.

**Authors:** Thank you for your suggestions. As your suggestions, we have already cited Ref#22 in the above mentioned sentence instead of Ref#19, 22 and updated the revised manuscript. Thanks again.

**R2-9:** Page 4, Data paragraph, in the sentence “**The analysis of smoking policies at workplace** … but outside their home (adults)“, “smoking policies” should be corrected into ‘smoke-free policies”. Also, were “adults” defined as **people aged 18+ or aged 15+**?

**Authors:** Thank you for your suggestions. We have already replaced the word “**smoking policies**” by “**smoking-free policies**” in the revised manuscript. In regarding adult, we are defined the word adult as people aged 15+. Thanks again.

**R2-10:** Page 4, Section 2.3:
**R2-10a:** In the 1st sentence, delete “The status of” and “consisted of four categories”.

**Authors:** Thank you for your suggestions. We have already removed the word “**The status of**” and “**consisted of four categories**” from our revised manuscript. Thanks again.

**R2-10b:** In the 2nd sentence, change the sentence “**To determine the correlates of complete smoke-free policies at home and workplace classified into binary in the following ways** “ to “Smoke-free policies at home and workplace were classified into binary in the following ways”.

**Authors:** Thank you for your suggestions. We have already fixed the above sentence and updated the revised manuscript. Thanks again.

**[Revised Manuscript Page #4; Section#2, Subsection#2.3, 2^nd^ sentence]**

**R2-10c.** If the outcome variable “smoke-free policies at home” is classified as a binary variable as stated in this section, why was this variable classified into a 4-group categorical variable in Table 1? The chi-square test for the association between 2-category smoke-free variable and the independent variable would have different results from the chi-square test for the association between 4-category smoke-free variable and the independent variable.

**Authors:** Thank you for your suggestions and giving us an opportunity to improve the revised manuscript. We are totally agreed with this reviewer. Now, we have made a Table 2 with the combination of previous Table 1 and Table 2 for two categories of outcome instead of 4-categories and updated the revised manuscript. Thanks again.

Table 1. Prevalence of smoke-free policies in the home and in the workplace

| Characteristics | Smoke-free policies in | | | | | |
| --- | --- | --- | --- | --- | --- | --- |
|  | Home | | | Workplace | | |
|  | Yes, % | No, % | p-value | Yes, % | No, % | p-value^1^ |
| Overall | 62.8 | 37.2 |  | 51.7 | 48.3 |  |
| **Tobacco Smoking status** |  |  | <0.001 |  |  | <0.001 |
| Smoker | 28.5 | 71.5 |  | 48.5 | 51.5 |  |
| Non-smoker | 56.1 | 43.9 |  | 66.9 | 33.1 |  |
| **Age group** |  |  | 0.944 |  |  | 0.676 |
| 15-30 | 51.8 | 48.2 |  | 63.6 | 36.4 |  |
| 31-45 | 51.8 | 48.2 |  | 61.8 | 38.2 |  |
| 46-60 | 51.3 | 48.7 |  | 62.7 | 37.3 |  |
| 60+ | 51.5 | 48.5 |  | 63.2 | 36.8 |  |
| **Gender** |  |  | 0.205 |  |  | <0.001 |
| Male | 52.2 | 47.8 |  | 61.6 | 38.4 |  |
| Female | 51.1 | 48.9 |  | 68.9 | 31.1 |  |
| **Religion** |  |  | <0.001 |  |  | 0.0086 |
| Hindhu | 51.9 | 48.1 |  | 63.5 | 36.5 |  |
| Muslim | 45.4 | 54.6 |  | 57.1 | 42.9 |  |
| Christian | 65.4 | 34.6 |  | 64.6 | 35.4 |  |
| Others | 35.0 | 65.0 |  | 68.5 | 31.5 |  |
| **Marital status** |  |  | <0.001 |  |  | <0.001 |
| Single | 56.0 | 44.0 |  | 69.0 | 31.0 |  |
| Married | 50.2 | 49.8 |  | 60.9 | 39.1 |  |
| Separated/others | 52.6 | 47.4 |  | 58.9 | 41.1 |  |
| **Place of residence** |  |  | <0.001 |  |  | <0.001 |
| Urban | 46.1 | 53.9 |  | 56.0 | 44.0 |  |
| Rural | 62.1 | 37.9 |  | 70.8 | 29.2 |  |
| **Region** |  |  | <0.001 |  |  | <0.001 |
| North | 56.3 | 53.7 |  | 62.3 | 37.7 |  |
| Central | 38.3 | 61.7 |  | 52.7 | 47.3 |  |
| East | 43.9 | 56.1 |  | 58.1 | 41.9 |  |
| North-east | 43.3 | 56.7 |  | 55.9 | 44.1 |  |
| West | 56.6 | 43.4 |  | 74.7 | 25.3 |  |
| South | 77.9 | 22.1 |  | 72.2 | 27.8 |  |
| **Education** |  |  | <0.001 |  |  | <0.001 |
| No education | 41.5 | 58.5 |  | 41.5 | 58.5 |  |
| Up to primary | 46.4 | 53.6 |  | 50.8 | 49.2 |  |
| Up to secondary | 53.6 | 46.4 |  | 64.8 | 35.2 |  |
| More than secondary | 65.1 | 34.9 |  | 76.3 | 23.7 |  |
| **Occupation** |  |  | <0.001 |  |  | <0.001 |
| Govt./ Non-Govt. employee | 60.7 | 39.3 |  | 75.5 | 24.5 |  |
| Daily wise/ causal labourer | 46.8 | 53.2 |  | 46.6 | 53.4 |  |
| Self employed | 49.1 | 50.9 |  | 58.2 | 41.8 |  |
| Homemaker | 59.6 | 40.4 |  | 84.0 | 16.0 |  |
| Student | 50.5 | 49.5 |  | 52.8 | 47.2 |  |
| Others ^(a)^ | 50.1 | 49.9 |  | 58.0 | 42.0 |  |
| **Household asset^(b)^** |  |  | <0.001 |  |  | <0.001 |
| Q_1_ (poorest) | 39.1 | 60.9 |  | 46.1 | 53.9 |  |
| Q_2_ | 48.7 | 51.3 |  | 56.7 | 43.3 |  |
| Q_3_ | 54.5 | 45.5 |  | 62.1 | 37.9 |  |
| Q_4_ | 62.8 | 37.2 |  | 74.2 | 25.8 |  |
| Q_5_ (richest) | 59.6 | 40.4 |  | 72.5 | 27.5 |  |
| **Other people’s smoking cause serious illness in children** |  |  | <0.001 |  |  | 0.484 |
| Yes | 52.1 | 47.9 |  | 62.9 | 37.1 |  |
| No | 43.4 | 56.6 |  | 60.0 | 40.0 |  |

Q1: Poorest; Q2: Poorer; Q3: Middle; Q4: Richer; Q5: Richest.

**[Revised Manuscript Page #15; Table#2]**

**R2-11:** Page 5, 1st paragraph: **The entire description of the independent variables was provided only in a supplementary table**. Because the main aim of this study is to determine whether the selected independent variables are significant correlates of smoke-free policies, the independent variables should be explained in great detail in the Methods section including their names, definitions, and how they were measures. For example, for the smoking status variable, how were “smokers” defined? What GATS questions were used to define “smokers”? For the “knowledgeable of the children illness”, what GATS questions were used to define this variable?

**Authors:** Thank you for your suggestions and giving us an opportunity to improve the revised manuscript. We are totally agreed with this reviewer. Now, we have added a Table in the method section for description of independent variables and update the revised manuscript. Thanks again.

**[Revised Manuscript Page #5; Table#1]**

**R2-12:** Page 5, Section 2.4, the authors checked multi-collinearity based on the values of SE for all predictors by citing Ref #23, which determined multi-collinearity by the criteria of whether the magnitude of the SE for all predictors was > 0.5 by citing a study “Chan Y (2004) Biostatistics 202: logistic regression analysis. SingapMed J 45:149–153).” However, in the original work by Chan (2004), it was stated that measuring multicollinearity by inspecting the magnitude of the standard error (SE) of each variable is a simple but subjective technique because there is no fixed criterion on how small the SE should be but a matter of judgment. In the literature, a more widely used technique to diagnose multicollinearity is the variance inflation factor (VIF).

**Authors:** Thank you for your suggestions. Yes, we are totally agreed with this reviewer. We are already run our logistic model with “**collin**” command (using STATA) after running logistic model and look VIF values for checking multicollinearity between independent variables. We observed that all VIF values were less than 10, indicating no significant multicollinearity between explanatory variables. Now, we have revised the Table and also updated the revised manuscript. Thanks again.

*Table 3. Multivariate logistic regression results*

| *Variable* | | *Home Place* | | | *Work Place* | | |
| --- | --- | --- | --- | --- | --- | --- | --- |
|  |  | *p-value* | *AOR (95% CI of AOR)* | *VIF* | *p-value* | *AOR (95% CI of AOR)* | *VIF* |
| ***Tobacco Smoking*** | *Smoker®* |  |  | *1.05* |  |  | *1.11* |
| ***status*** | *Non-smoker* | *<0.001* | *3.285 (3.001,3.595)* |  | *<0.001* | *1.627 (1.404, 1.886)* |  |
| ***Age group*** | *15-30* |  | *-* |  |  | *-* |  |
|  | *31-45* |  | *-* |  |  | *-* |  |
|  | *46-60* |  | *-* |  |  | *-* |  |
|  | *60+* |  | *-* |  |  | *-* |  |
| ***Gender*** | *Male®* |  | *-* |  |  |  | *1.11* |
|  | *Female* |  | *-* |  | *0.048* | *1.229 (1.002, 1.510)* |  |
| ***Marital status*** | *Single®* |  |  | *1.13* |  |  | *1.06* |
|  | *Married* | *0.420* | *0.961 (0.874, 1.058)* |  | *0.604* | *0.954 (0.797, 1.141)* |  |
|  | *Separated/others* | *0.274* | *1.081 (0.940, 1.242)* |  | *0.934* | *0.984 (0.677, 1.431)* |  |
| ***Religion*** | *Hindhu®* |  |  | *1.01* |  |  | *1.01* |
|  | *Muslim* | *0.074* | *0.885 (0.774, 1.012)* |  | *0.510* | *1.069 (0.876, 1.305)* |  |
|  | *Christian* | *0.901* | *0.987 (0.805, 1.211)* |  | *0.084* | *0.710 (0.482, 1.047)* |  |
|  | *Others* | *<0.001* | *1.939 (1.650, 2.278)* |  | *0.589* | *1.099 (0.778, 1.554)* |  |
| ***Place of residence*** | *Urban®* |  |  | *1.21* |  |  | *1.19* |
|  | *Rural* | *<0.001* | *1.376 (1.253, 1.511)* |  | *0.015* | *1.245 (1.044, 1.484)* |  |
| ***Region*** | *North®* |  |  | *1.01* |  |  | *1.01* |
|  | *Central* | *0.434* | *0.952 (0.840, 1.077)* |  | *0.170* | *0.866 (0.706, 1.064)* |  |
|  | *East* | *<0.001* | *1.249 (1.107, 1.408)* |  | *0.003* | *1.424 (1.132, 1.791)* |  |
|  | *North-east* | *0.001* | *1.258 (1.095, 1.445)* |  | *0.162* | *1.181 (0.935, 1.491)* |  |
|  | *West* | *<0.001* | *1.637 (1.423, 1.885)* |  | *0.001* | *1.671 (1.247, 2.239)* |  |
|  | *South* | *<0.001* | *5.339 (4.764, 5.985)* |  | *<0.001* | *1.836 (1.476, 2.285)* |  |
| ***Education*** | *Noeducation®* |  |  | *1.44* |  |  | *1.46* |
|  | *Uptoprimary* | *0.004* | *1.127 (1.038, 1.222)* |  | *0.001* | *1.356 (1.124, 1.636)* |  |
|  | *Upto secondary* | *<0.001* | *1.401 (1.286, 1.527)* |  | *<0.001* | *1.831 (.501, 2.235)* |  |
|  | *Morethan* | *<0.001* | *1.879 (1.695, 2.083)* |  | *<0.001* | *2.317 (1.834, 2.928)* |  |
|  | *secondary* |  |  |  |  |  |  |
| ***Occupation*** | *Govt./Non-Govt.* |  |  | *1.07* |  |  | *1.10* |
|  | *employee®* |  |  |  |  |  |  |
|  | *Dailywise/causal*  *laborer* | *0.008* | *0.853 (0.758, 0.959)* |  | *<0.001* | *0.468 (0.381, 0.574)* |  |
|  | *Selfemployed* | *0.094* | *0.913 (0.821, 1.016)* |  | *<0.001* | *0.559 (0.458, 0.684)* |  |
|  | *Homemaker* | *0.530* | *1.047 (0.908, 1.206)* |  | *0.001* | *2.064 (1.327, 3.208)* |  |
|  | *Student* | *0.001* | *0.821 (0.733, 0.919)* |  | *0.001* | *0.495 (0.329, 0.743)* |  |
|  | *Others* | *0.025* | *0.856 (0.746, 0.981)* |  | *0.095* | *0.629 (0.366, 1.083)* |  |
| ***Wealth quintile*** | *Q_1_(poorest)®* |  |  | *1.41* |  |  | *1.47* |
|  | *Q_2_* | *0.623* | *0.977 (0.889, 1.072)* |  | *0.121* | *1.173 (0.959, 1.435)* |  |
|  | *Q_3_* | *0.170* | *1.076 (0.969, 1.195)* |  | *0.051* | *1.248 (0.998, 1.559)* |  |
|  | *Q_4_* | *0.018* | *1.176 (1.028, 1.346)* |  | *0.016* | *1.378 (1.061, 1.789)* |  |
|  | *Q_5_(richest)* | *0.108* | *1.103 (0.979, 1.243)* |  | *0.007* | *1.416 (1.101, 1.820)* |  |
| ***Otherpeople’s*** | *No®* |  |  | *1.02* |  |  |  |
| ***Smokingcauseserious***  ***Illnessinchildren*** | *Yes* | *0.003* | *1.219 (1.068, 1.391)* |  |  | *-* |  |

*- indicates that the variable is insignificant in bivariate analysis*

**[Revised Manuscript Page #15; Table #2]**

**R2-13:** On page 6, 1st paragraph, the phrase “by conducting bivariate analysis“ and the sentence “A Chi-square test was used to assess the association … with selected factors. “ should be moved to the Methods section.

**Authors:** Thank you for your suggestions. We are already moved this sentence from Results section to Methods section and updated the revised manuscript.

**[Revised Manuscript Page #5; Section#2, Subsection#2.4]**

**R2-14:** Page 6, 1st paragraph, in the sentence “… among the participants 54.4% had complete smoke-free policies at their home, 15.6% had partial … and 34% had no rules … (see Table 1)“, there are two problems. First, the sum of 54.4%, 15.6%, and 34% equals 104.0%, which is greater than 100%. There must be typos. Second, these numbers were not shown in Table 1. Similarly, in the next sentence, the reported percentages for smoke-free policy at workplace were not shown in Table 2.

**Authors:** Thank you for your suggestions. The manuscript has been revised and modified by considering your instruction. Now, we have made a Table 2 with the combination of previous Table 1 and Table 2 for two categories of outcome instead of 4-categories and updated the revised manuscript. Thanks again.

Table 2. Prevalence of smoke-free policies in the home and in the workplace

| Characteristics | Smoke-free policies in | | | | | |
| --- | --- | --- | --- | --- | --- | --- |
|  | Home | | | Workplace | | |
|  | Yes, % | No, % | p-value | Yes, % | No, % | p-value^1^ |
| **Overall** | 62.8 | 37.2 |  | 51.7 | 48.3 |  |
| **Tobacco Smoking status** |  |  | <0.001 |  |  | <0.001 |
| Smoker | 28.5 | 71.5 |  | 48.5 | 51.5 |  |
| Non-smoker | 56.1 | 43.9 |  | 66.9 | 33.1 |  |
| **Age group** |  |  | 0.944 |  |  | 0.676 |
| 15-30 | 51.8 | 48.2 |  | 63.6 | 36.4 |  |
| 31-45 | 51.8 | 48.2 |  | 61.8 | 38.2 |  |
| 46-60 | 51.3 | 48.7 |  | 62.7 | 37.3 |  |
| 60+ | 51.5 | 48.5 |  | 63.2 | 36.8 |  |
| **Gender** |  |  | 0.205 |  |  | <0.001 |
| Male | 52.2 | 47.8 |  | 61.6 | 38.4 |  |
| Female | 51.1 | 48.9 |  | 68.9 | 31.1 |  |
| **Religion** |  |  | <0.001 |  |  | 0.0086 |
| Hindhu | 51.9 | 48.1 |  | 63.5 | 36.5 |  |
| Muslim | 45.4 | 54.6 |  | 57.1 | 42.9 |  |
| Christian | 65.4 | 34.6 |  | 64.6 | 35.4 |  |
| Others | 35.0 | 65.0 |  | 68.5 | 31.5 |  |
| **Marital status** |  |  | <0.001 |  |  | <0.001 |
| Single | 56.0 | 44.0 |  | 69.0 | 31.0 |  |
| Married | 50.2 | 49.8 |  | 60.9 | 39.1 |  |
| Separated/others | 52.6 | 47.4 |  | 58.9 | 41.1 |  |
| **Place of residence** |  |  | <0.001 |  |  | <0.001 |
| Urban | 46.1 | 53.9 |  | 56.0 | 44.0 |  |
| Rural | 62.1 | 37.9 |  | 70.8 | 29.2 |  |
| **Region** |  |  | <0.001 |  |  | <0.001 |
| North | 56.3 | 53.7 |  | 62.3 | 37.7 |  |
| Central | 38.3 | 61.7 |  | 52.7 | 47.3 |  |
| East | 43.9 | 56.1 |  | 58.1 | 41.9 |  |
| North-east | 43.3 | 56.7 |  | 55.9 | 44.1 |  |
| West | 56.6 | 43.4 |  | 74.7 | 25.3 |  |
| South | 77.9 | 22.1 |  | 72.2 | 27.8 |  |
| **Education** |  |  | <0.001 |  |  | <0.001 |
| No education | 41.5 | 58.5 |  | 41.5 | 58.5 |  |
| Up to primary | 46.4 | 53.6 |  | 50.8 | 49.2 |  |
| Up to secondary | 53.6 | 46.4 |  | 64.8 | 35.2 |  |
| More than secondary | 65.1 | 34.9 |  | 76.3 | 23.7 |  |
| **Occupation** |  |  | <0.001 |  |  | <0.001 |
| Govt./ Non-Govt. employee | 60.7 | 39.3 |  | 75.5 | 24.5 |  |
| Daily wise/ causal labourer | 46.8 | 53.2 |  | 46.6 | 53.4 |  |
| Self employed | 49.1 | 50.9 |  | 58.2 | 41.8 |  |
| Homemaker | 59.6 | 40.4 |  | 84.0 | 16.0 |  |
| Student | 50.5 | 49.5 |  | 52.8 | 47.2 |  |
| Others ^(a)^ | 50.1 | 49.9 |  | 58.0 | 42.0 |  |
| **Household asset^(b)^** |  |  | <0.001 |  |  | <0.001 |
| Q_1_ (poorest) | 39.1 | 60.9 |  | 46.1 | 53.9 |  |
| Q_2_ | 48.7 | 51.3 |  | 56.7 | 43.3 |  |
| Q_3_ | 54.5 | 45.5 |  | 62.1 | 37.9 |  |
| Q_4_ | 62.8 | 37.2 |  | 74.2 | 25.8 |  |
| Q_5_ (richest) | 59.6 | 40.4 |  | 72.5 | 27.5 |  |
| **Other people’s smoking cause serious illness in children** |  |  | <0.001 |  |  | 0.484 |
| Yes | 52.1 | 47.9 |  | 62.9 | 37.1 |  |
| No | 43.4 | 56.6 |  | 60.0 | 40.0 |  |

Q1: Poorest; Q2: Poorer; Q3: Middle; Q4: Richer; Q5: Richest.

**[Revised Manuscript Page #15; Table#2]**

**R2-15:** Pages 6-7, the Results section needs to be substantially revised to improve the clarity. Some of the reported results (including Table 3) do not seem reasonable.
a. For example, in the sentence, “.. elderly people were more likely to enjoy a completely smoke-free environment at their home than the younger counterparts.”, who are the elderly people? Was the group aged 31-45 regarded as “elderly people”? What are the younger counterparts? Is this statement justified by the results in Table 3? I am puzzled why the AORs for the age groups 31-45, 46-60, and ≥60 were greater than 1 and highly significantly (p <.001) while the prevalence of smoke-free home rule was very similar across age groups (49.2% for the 15-30 group, 50.8% for the 31-45 group, 51.6% for the 46-60 group, and 50.6% for the ≥60 group. Was the sample weight used for the logistic regression analysis? I wonder whether the highly statistically significant results were due to the inflated sample size from using the sample weight. If this is the case, the models need to be re-estimated using the normalized weight.
b. A sentence states that “Female counterparts had less likely tendency to live in a complete smoke-free atmosphere in their residence compared to the male counterparts.” However, according to Table 1, the prevalence of smoke-free home rules was 50.7% for females and 50.0% for males. Their prevalence rates are quite close. It is peculiar why the AOR for female compared to male was less than 1 (AOR=0.569) and highly significant (p <.001).
c. Another sentence states that “Participants who lived in rural areas were 1.211 times more likely to report that they worked in a completely smoke-free environment than their counterparts.” However, according to Table 2, the prevalence of smoke-free workplace rule in rural areas was 52.1%, which is much lower than that in urban areas (69.4%). This illustrated that the reliability of Table 3 results is questionable.
d. The sentence “Participants who lived in the South, East, and West region of India had the higher tendency to report that they worked in a completely smoke-free environment compared to other regions” is not justifiable by the Table 3 results.

**Authors:** Thank you for your suggestions and giving us an opportunity to improve the revised manuscript. We have already revised the results section and update the revised manuscript. Thanks again.

**[Revised Manuscript Page #6-7; Section#3 (Results)]**

**R2-16:** On page 7, the 2nd paragraph, the phrase “**According to a research study**,” should be deleted.

**Authors:** Thank you for your suggestions. The manuscript has been revised and modified by considering your instruction.

**R2-17:** Pages 8-9, the Discussion section needs substantial revision by rigorously comparing the findings of this study with the literature, e.g,. how the findings are similar to or different from the previously published studies in the field, rather than repeating what has been presented in the Results section.

**Authors:** Thank you for your suggestions. The manuscript has been revised and modified by considering your instruction.

**R2-18:** On page 8, the 2nd paragraph, the sentence “**Married and separated people were more habituated to smoking in their workplace**” is inconsistent with the results shown in **Table 3**.

**Authors:** Thank you for your suggestions. We have already revised this sentence in discussion section and updated the revised manuscript. Thanks again.

**R2-19:** On page 9, the 1st paragraph, the meaning of the 4 sentences (“The research paper demonstrated that …. **It could be detrimental for children in a two-fold way**. They could fall victim …. But, if the children belonged to a highly educated and high income family, the exposure risk would decrease“) is not clear. How are these sentences relevant to the findings of this study?

**Authors:** Thank you for your suggestions. We are totally agreed with this reviewer. Now, we have removed these sentences from discussion and also revised the discussion section in the revised manuscript. Thanks again.

**R2-20:** On page 9, the 2nd paragraph, the sentence “For instance, such as section 4 (smoking in public places-59.28%), 6-a (implementation level-68.57%) and 6-b (implementation level-52.85%) were observed to be violated to some extent [37].“ What does “**implementation level**” mean? How was it measured?
**Authors:** Thank you for your suggestions. We have collected the above statistics from Ali et al. (2020). They used an assessment tools to measure the implementation tools which were explained in details in the following paper:

Ali, I., Patthi, B., Singla, A., Dhama, K., Muchhal, M., Rajeev, A., ...& Khan, A. (2020). Assessment of implementation and compliance of (COTPA) Cigarette and Other Tobacco Products Act (2003) in open places of Delhi. Journal of Family Medicine and Primary Care, 9(6), 3094.

**R2-21:** On Page 10, the authors cited a study conducted in the United States to explain the limitation of the GATS survey. This does not make sense.

**Authors:** Thank you for your suggestions. We are extremely sorry for this great mistake. Now, we have removed this sentence from strengths and limitations and updated the revised manuscript. Thanks again.

**R2-22:** Page 10, the last sentence — “… so that the projection of WHO about India being the only South Asian country to curb its smoking prevalence by 30% within 2025 will turn to be a success” is ambiguous. First, it needs to cite a reference about the WHO projected goal of smoking prevalence by 2014 for India. Second, it needs to explain what the current smoking prevalence in India is. This sentence seems to be contradictory to the information presented in the Introduction section (Page 3, lines 8-9): “Findings from the Global Adult Tobacco Survey 2 (GATS-2)-2017 indicate that 266.8 (28.6%) millions of all adults use tobacco in any form in India where 10.7% smoke tobacco”. Wasn’t India’s cigarette smoking prevalence rate in 2016-2017 10.7%?

**Authors:** Thank you for your suggestions. We are totally agreed this reviewer. We have omitted this sentence from conclusion and updated the revised manuscript. Thanks again.

**R2-23:** Tables 1-2 need to **add a row to show** results for all the persons in the final study sample.
**Authors:** Thank you for your suggestions. We have already made a new Table with the combination of Table 1 and Table 2 and add a row to show results for all the persons in the final study sample as follows:

| Characteristics | Living in a complete smoke-free policies (n= 15,254) | | | Working in a complete smoke-free policies (n= 71,046) | | |
| --- | --- | --- | --- | --- | --- | --- |
|  | Yes % | No, % | p-value | Yes, % | No, % | p-value^1^ |
| Overall | 62.8 | 37.2 |  | 51.7 | 48.3 |  |

**R2-24:** Tables 3-4 need to show the total sample size for the analysis.
**Authors:** Thank you for your suggestions. The manuscript has been revised and modified by considering your instruction.

**R2-25:** In the Supplementary Table A1, for the age variable, “**Up to 60 years**” should be corrected into “**More than 60 years**”.

**Authors:** Thank you for your suggestions. We have already replaced Up to 60 years by More than 60 years in the In the Supplementary Table A1 and updated the revised manuscript. Thanks again.

**R2-26:** Inconsistent terms:
**R2-26a:** Smoke-free policies vs. smoking policies. The term “**smoking policies**” used in several places (e.g., page 4, line 2) should be corrected into “**smoke-free policies**”.

**Authors:** Thank you for your suggestions. We have already replaced the word “smoking policies” by smoke-free policies throughout the manuscript and updated the revised manuscript. Thanks again.

**[Revised Manuscript Page #4; Section#2, Subsection#2.1]**

**R2-26b:** Outcome variables & independent variables (Section 2.3, line 1 and line 13) vs. dependent variables & explanatory variables (page 5, Section 2.4, line 3).

**Authors:** Thank you for your suggestions. We have already fixed it and updated the revised manuscript. Thanks again.

**[Revised Manuscript Page #4, 5; Section#2, Subsection#2.3, 2.4]**

**R2-26c:** GATS2 vs. GATS 2

**Authors:** Thank you for your suggestions. We have already replaced the word “GATS2” by “GATS 2” throughout the manuscript. Thanks again.

**R2-27:** Inappropriate terms: “**secondhand smoker’s exposure**” should be corrected into “**secondhand smoke exposure**”.

**Authors:** Thank you for your suggestions. The manuscript has been revised and modified by considering your instruction.
