# Supplementary material for: Smoke-free status of homes and workplaces among Indian people: Evidence from Global Adult Tobacco SurveyData-2016/2017
Source: PLoS One. 2023 Feb 23;18(2):e0282138. doi: 10.1371/journal.pone.0282138 (PMC9949653; doi:10.1371/journal.pone.0282138)
Supplement: Attachment — Submitted filename: R2-Rebuttal-Ver2.docx [file pone.0282138.s005.docx]

**Authors’ Response to Reviewers**

Manuscript ID: PONE-D-20-41034R2

**Title: Smoke-free policies in the home and in the workplace among Indian people: Evidence from Global Adult Tobacco Survey Data-2017**

Dear Respected Editor-in-Chief,

First of all, we thank the reviewer and the editor for their useful suggestions for improving the manuscript. We have now revised the manuscript accordingly. Thank you once again and looking forward to the final acceptance in your esteemed journal.

**Reviewer (R3):**

More should be said about the smoke-free policies and their enforcement. It is not clear why, if there are smoke-free policies, people still smoke at workplaces, e.g. More should be said about how policies have been implemented, i.e., if there are fines in case of non-compliance, and it there is some monitoring. I have added some lines. Please Check:

**R3-1**: The dependent variable should not be called smoke-free “policies” since authors are not evaluating policies. Instead, it should be named **smoking “norms**”, e.g.

**Authors:** Thank you very much for your valuable suggestions and for giving us an opportunity to improve the revised manuscript. the comment. As per your suggestions, we have replaced the word **“smoke-free policies”** with **“smoking norms”** throughout the manuscript and updated the revised manuscript. Thanks for understanding in advance.

**R3-2**: Authors have been including several variables, but **do not provide any justification to include them**. It seems more they have been “fishing” factors, without any clear theoretical background to consider specific variables. In the same line, the **Discussion fails to provide explanations for why women, richer and more educated people live and work in more smoke-free environments**.

**Authors:** Thanks again for asking this question and giving us an opportunity to improve the revised manuscript. We apologize for the unclear specifications of factors. Based on the previous literature, we have selected the input variables or factors. For the justification, we have added some references in the descriptions of the dataset section, and the findings of our significant factors are clearly explained in the discussion section. Thanks in advance for understanding.

**R3-3**: The correlation between **SHS and smoke-free policies** is not convincing as it does not consider **potential confounders**, as **this is the case in the main analysis**.

**Authors:** Thanks very much for your valuable comments. In this study, we try to only show the correlation between SHS and smoke-free policies. We do not try to consider SHS as a potential confounder of the smoke-free policies. As per your concerns, we will try to verify whether it is a potential confounder or not in our next projects. Thanks in advance for understanding.

**R3-4:** There is a clear problem of **reverse causation** when using the smoker variable, as living in a more smoke-free environment reduces the likelihood to smoke. **To avoid this problem**, authors may use **the prevalence of smoking in the region or municipality**, e.g.

**Authors:** Thanks for asking this question. We are totally agreed with you. But, this study aimed to determine the impact of correlates on smoking norms both at home and workplaces in India. Without adjusting smoker variable in our main analysis, we could not find out the other correlates exactly. When we perform regression analysis, the independent variables asymptotically fixed and only dependent variable is considered as random variable. So, we think that it does not create any big problems for interpretation. Thanks in advance for understanding.

**R3-5:** More generally, the implementation of norms is related to contextual variables, namely, the degree of enforcement of policies, cultural perceptions about smoking, other anti-tobacco policies (taxes, sales bans to minors, etc), or communication strategies by local authorities.

**Authors:** Thanks for your comments. We are totally agreed with you. We are already discussed in the discussion in the revised manuscript. We do not consider these variables in the main analysis because these variables are not available in our data. Thanks in advance for understanding.

**R3-6:** The relevance of the paper is not clearly highlighted. Authors should state why measuring the determinants of smoke-free environments is relevant from a public health viewpoint, and how results may be relevant for decision making. We miss a section with the **policy implications of the paper**.

**Authors:** Thanks again for asking this question and giving us an opportunity to improve the revised manuscript. We already updated it’s in the revised manuscript. Thanks in advance for understanding.

**Dear Reviewer#3**

Thank you very much for your encouragement, kind guidance, and support in helping the authors improve the manuscript.

We are truly grateful for your time and support.

Kind Regards,

The Authors of the manuscript.

**Reviewer (R4):**

The authors have responded to all comments by the previous reviewers. However, some major language revision is still needed (**especially the results and discussion sections**) to help improve the readability of the manuscript.

**Abstract**
**R4-1:** Some of the sentences needs restructuring to make it read better.

**Authors:** Thank you very much for your valuable suggestions and giving us an opportunity to improve the revised manuscript. As per your suggestions, we have restructured some sentences in the abstract for the readers better understanding and updated the revised manuscript. Thanks, in advanced for understanding.

**Introduction**
**R4-2:** As mentioned by previous reviewers, please elaborate on the importance of the second objective (i.e. correlation between state-level smoke free policy and SHS exposure).

**Authors:** Thank you very much for your valuable suggestions and giving us an opportunity to improve the revised manuscript. As per your suggestions, we have updated the introduction section in the revised manuscript Thanks, in advanced for understanding.

**Results**
**R4-3:** In many places, the sentences need to be rewritten. For example: Page 6 Line 15 “About 28.5% of tobacco smokers were kept to complete smoke-free policies in the home, whereas 48.5% of smokers in the workplace”. The meaning of this sentence is unclear. Do the authors mean “25% and 48.5% of tobacco smokers have smoke-free policies in their homes and workplace respectively?”

**Authors:** Thank you very much for your valuable suggestions and giving us an opportunity to improve the revised manuscript. As per your suggestions, we have restructured this sentence for readers clear understanding in result section and updated the revised manuscript. in the revised manuscript. Thanks again for understanding.

**Table 2:**
**R4-4:** To indicate **which variables were adjusted for in the multivariable model**?

**Authors:** Thank you very much for your concerns and giving us an opportunity to improve the revised manuscript. In this study, we have applied multivariable or multiple logistic regression model. Suppose we have three independent factors, say A, B, and C which are significantly associated with dependent variable “Y”. These three factors are also considered as a significant factor under simple logistic regression (have only one independent variable with a dependent variable). In multivariable logistic regression model needs to done included all significant factors. Now, the odds ratio of independent factors “A” under simple logistic regression is unadjusted odds ratio while under multivariable logistic regression, it is adjusted odds ratio adjusting for the risk factor such as “B” and “C”.

In case of our study, we have 9 significant factors for smoking-free policies in home, get from Chi-Square test (p<0.05). Using these 9 significant factors, we fitted our multivariable logistic regression model and calculated the odds ratio. The odds ratio of “Tobacco smoking status” is called adjusted adjusting for the rests of 8 risk factors. We hope that it is clear now. Thanks again for understanding.

**R4-5:** I suggest that the table be titled differently to better describe its contents. A suggestion is “**Correlates of smoke-free policies in the home and the workplace in India**”

**Authors:** Thank you very much for your valuable suggestions and giving us an opportunity to improve the revised manuscript. As per your suggestions, we have updated the entitled of the **Table 2 as** “**Correlates of smoke-free policies in the home and the workplace in India**”. Thanks again for understanding.

**R4-6:** Th occupation ‘daily wise/causal labourer’ is probably meant to be ‘daily wage/casual labourer’?

**Authors:** Thank you very much for your valuable suggestions and giving us an opportunity to improve the revised manuscript. As per your suggestions, we have replaced the categories of occupations from **‘daily wise/causal labourer’** to **‘daily wage/casual labourer’** throughout the revised manuscript. Thanks again.

**Table 3:**

**R4-7:** As pointed out by a previous reviewer, the word multivariable is appropriate here rather than **multivariate logistic regression** (The terms are not interchangeable).

**Authors:** Thank you very much for your valuable suggestions and giving us an opportunity to improve the revised manuscript. As per your suggestions, we have replaced the word from **“multivariate logistic regression”** to **“multivariable logistic regression”** throughout the revised manuscript. Thanks again.

**R4-8:** ORs and the 95% CIs need be expressed up to 2 decimals only.

**Authors:** Thank again for your valuable suggestions and giving us an opportunity to improve the revised manuscript. As per your suggestions, we have added the value of **ORs along with their 95% CI in 2 decimals** throughout the revised manuscript. Thanks again.

**R4-9:** The use of the ‘’registered’ ® symbol to indicate the reference category is rather inappropriate (Or does this symbol appear only on my computer?). The authors may put ‘1’, ‘Reference’ or ‘.ref’ in the AOR for the reference category instead to indicate this.

**Authors:** Thank again for your valuable suggestions and giving us an opportunity to improve the revised manuscript. As per your suggestions, we have fixed it and updated the revised manuscript. Thanks again.

**Table 4:**

**R4-10:** The table should be given a more descriptive title e.g. “**Correlations between smoke-free policies in the home and workplace and secondhanded smoke exposure**”

**Authors:** Thank again for your valuable suggestions and giving us an opportunity to improve the revised manuscript. As per your suggestions, we have updated the entitled of the **Table 4 as** “**Correlations between smoke-free policies in the home and workplace and secondhand smoke exposure**”. Thanks again for understanding.

**R4-11:** Only half of the table cells need to be filled as the other half is redundant (have the same values).

**Authors:** Thank again for your valuable suggestions and giving us an opportunity to improve the revised manuscript. As per your suggestions, we have fixed the Table 4 and updated the revised manuscript. Thanks again.

**R4-12:** The correlation coefficient may be reported to only 2 decimals.

**Authors:** Thank again for your valuable suggestions and giving us an opportunity to improve the revised manuscript. As per your suggestions, we have kept the value of **reported correlation coefficients in 2 decimals** throughout the revised manuscript. Thanks again.

**R4-13:** ‘SHS at home’ should be ‘**SHS exposure at home**’ (similarly for workplace and public places).

**Authors:** Thank again for your valuable concerns. As per your suggestions, we have fixed these words throughout the manuscript these words and updated the revised manuscript. Thanks again for understanding.

**R4-14:** In the footnote, SHS should stand for secondhand smoke instead of secondhand smoker.

**Authors:** Thank again for your valuable suggestions and giving us an opportunity to improve the revised manuscript. As per your suggestions, we have modified the footnote of **Table 4** and updated the revised manuscript. Thanks again for understanding.

**Discussion**
Suggest to first summarize the finding and then interpretation and discussion in each paragraph. For example on Pg 8 para 3, it would help to state the correlation finding first before explaining the possible reasons why it is so.
The language style throughout the section need to be given a another round of proofreading.
- Example on Pg 8 “Literature suggests the fact that” should be “literature suggest that”
Pg 8, “Other ocupations tend to..” does “other occupations” mean non-professional occupations?
Pg 8: The statement “unspoiled , contaminated environment”, unspoiled and contaminated are contradictory, please check this statement.

**Authors:** Thank again for your valuable suggestions and giving us an opportunity to improve the revised manuscript. As per your suggestions, we have rewritten the discussion section and update the revised manuscript. Thanks in advance.

**Conclusion**
The implications of the study findings with regards to tobacco control policy as commented by the previous reviewer are still not spelled out. **What can the authorities do with the findings? Make more specific suggestions/recommendations**.

**Authors:** Thank again for your valuable suggestions and giving us an opportunity to improve the revised manuscript. As per your suggestions, we have rewritten the conclusion section and update the revised manuscript. Thanks in advance.

**Dear Reviewer#4**

Thank you very much for your encouragement, kind guidance, and support in helping the authors improve the manuscript.

We are truly grateful for your time and support.

Kind Regards,

The Authors of the manuscript.
