# Supplementary material for: Smoke-free status of homes and workplaces among Indian people: Evidence from Global Adult Tobacco SurveyData-2016/2017
Source: PLoS One. 2023 Feb 23;18(2):e0282138. doi: 10.1371/journal.pone.0282138 (PMC9949653; doi:10.1371/journal.pone.0282138)
Supplement: Attachment — Submitted filename: PLOS_2ndRevision.docx [file pone.0282138.s006.docx]

The paper has been improved but important limitations remain:

- We still miss a justification of the variables’ choice. Mentioning that they have been used in the literature is not enough. We must understand which mechanisms may lead subgroups to adopt stricter norms or not. In the Discussion, no good explanations are presented for some groups adopting stricter norms (e.g., more educated or richer people).
- Since this information is missing about potential causal mechanisms, the Discussion does not provide relevant insights for decision making.
- The text needs a careful revision of English language.
