# Supplementary material for: Smoke-free status of homes and workplaces among Indian people: Evidence from Global Adult Tobacco SurveyData-2016/2017
Source: PLoS One. 2023 Feb 23;18(2):e0282138. doi: 10.1371/journal.pone.0282138 (PMC9949653; doi:10.1371/journal.pone.0282138)
Supplement: Attachment — Submitted filename: R4-Rebuttal-Ver4.docx [file pone.0282138.s010.docx]

**Authors’ Response to Reviewers**

**Manuscript ID: PONE-D-20-41034R3**

**Title: Smoke-free policies in the home and in the workplace among**

**Indian people: Evidence from Global Adult Tobacco Survey Data-2017**

**Dear Respected Editor-in-Chief,**

We would like to express our gratitude and thanks to the reviewer and the editor for their useful suggestions for improving the manuscript. We have revised the manuscript accordingly. Thank you once again and looking forward to the final acceptance in your esteemed journal.

**Reviewer (R5)**

Overall very useful topic, but these substantial revisions, and language editing.

**R5-1:** The prevalence of male and female tobacco consumers is 42.4% and 14.2%. The denominator is not clear here.

**Authors:** Thank you for your comment. As per your concerns, we modified this sentence for more clear understanding and cited its supporting documents. Thanks again for understanding.

**R5-2:** References 6 and 13 in page number 3. How do these references say that there is progress in smoke-free policies in indoor public/workplaces

**Authors:** Thank you very much for raising this question. In reference 6 there is section of “Change in exposure to secondhand smoke at various places; GATS 1 to GATS 2” where we noticed the change in exposure to secondhand smoke at different public places among all adults, from GATS 1 India, 2009-10 to GATS 2 India, 2016-17. This difference in SHS exposure from GATS 1 to GATS 2 data may reflect the impact of law enforcement and increased public awareness in support of smoke-free policy. It indicates the progress of smoke free policy across the country. Based on that evidence this reference is placed here. We hope this will support the justification of using this reference.

**R5-3:** The lack of evidence on smoke-free policies in homes and suboptimal implementation of smoke-free workplaces need to be highlighted to provide a rationale for doing this study.

**Authors:** Thank you very much for your kind suggestion. We have included some information to provide justification and evidence on suboptimal implementation of smoke-free policy both at home and workplaces to highlight the rationale of the study.

**R5-4:** Page number 05: “It is classified into binary categories in the following ways: the respondent was classified as living in a completely smoke-free home”
Living in a smoke-free home is a misnomer here as we don’t know whether the home is smoke-free or not. We only know that there is smoke-free policy in the home. So, you have to choose these words carefully throughout the manuscript. So, we can say that the categories are: having a smoke-free policy at home or not?

**Authors:** Thank you so much for your valuable suggestion. As per your suggestions, we have modified this statement in the revised manuscript. Thanks again for understanding.

**R5-5:** Table 3: Please put p-value column on the right side of the a OR column.

**Authors:** Thank again for your valuable suggestion. As per your suggestion, we put the p-vale column of the right side of OR column and modified the Table 3 in the revised manuscript. Thanks again for understanding.

**R5-6:** Table 4: please clearly state that these correlations depict relationships at a state level and also write the total number (N) for each correlation.

Authors: Thank you very much for your valuable comments and concerns. As per your concerns, we put the total number (N) for each correction in Table and also modified Table 4 in the revised manuscript. Thanks again for understanding.

**R5-7:** Major comments: There is a major confusion in this paper. You are defining two things in the methods section (variables of the study sub-section): smoke-free policy in home and workplace and exposure to SHS in home and workplace. Both are different concepts as rightly defined. However, in the results you are probably exploring correlates of smoke-free policy. Why do you define SHS exposure under the variables of the study section?

**Authors:** Thank you very much for giving your valuation concerns. As per your concerns, we move this part from here and make another subsection as study or dependent variable under methodology section in the revised manuscript. Yes, we estimated the state-level correlation between the proportion of smoke-free policies and SHS exposure at both home and workplace. To make people understand what SHS is and how it is defined, it is added to the study section.

**R5-8:** Out of 74, 037 we chose 71,046 and 15,254 for SHS at home and workplace respectively. How did you arrive at these numbers? Please provide a flowchart showing the flow of respondents.

**Authors:** Thank you very much for your valuable concerns. As per your concerns, we added a flowchart for both SHS at home and workplace in the revised manuscript. Thanks again for understanding.

**R5-9:** Discussion: 65 and above are mostly tended to stay smoke-free in their home h. Please rectify this sentence.

**Authors:** Thank you very much for your valuable comments. It was typing mistake. We apologized for such a mistake. This sentence is corrected and updated in the revised manuscript. Thanks again for understanding.

**R510:** “female people emphasized more on a smoke-free zone in their workplace more than their opposite gender”
“Married people were less tending to enjoy a complete non-smoking work environment as well as their home than their single counterparts”
“Contrarily, separated persons were expected to enjoy a more smokefree environment in their residence.”
The use of verbs such as emphasize, tending to enjoy, expected to enjoy does not seem to fit your findings. Rephrasing these statements would help.

**Authors:** Thank you very much for your valuable suggestions. As per your suggestions, all these terms have been removed from the discussion section in order to make the statements more perceptible. Thanks again for understanding.

**R5-11:** Muslims were more likely to maintain a smoke-free workplace but not a smoke-free home. The use of religion as an explanation seems contradicting here.
Any possible reason/speculation for the regional differences in smoke-free policies? Exploring the answers and possible speculations require a thorough understanding of the country’s culture, tobacco habits, regional differences which might not be the case here as all the authors belong to another country i.e. Bangladesh. I wonder what is the rationale for conducting this study in an Indian setting.

**Authors:** Thank you very much for comments. Yes, you are right. In our previous version revised manuscript, we mistakenly written this statement. We are extremely sorry regarding this issue. Actually, we got only other religion people as a significant risk factors compared to Hindu religions. for completely smoke-free policy at home. In this work, we observed that other religious people were 1.939 [AOR=1.939, 95% CI= 1.65 to 2.28, p<0.001] times more likely to follow complete smoke-free policy at home compared to Hindu religious people. As per your concerns, we fixed this statement in the discussion section of the revised manuscript. Thanks again for understanding.

**R5-12:** Any possible reason/speculation for the **association of education and occupation with Smoke free policy**. Discussion section lacks a paragraph on policy implications based on the key findings of the study

**Authors:** Thank you very much for noticing this point. We have tried to highlight the possible reasons of association for education and occupation with Smoke free policy. Besides, based on the study findings, possible policy implications are added to the conclusion section. Thanks again for understanding.

**R5-13:** “However, we didn’t adjust some other possible confounding factors which could be explored further”. Mention what are the possible confounders which could be explored further.

**Authors:** Thank you very much for your comment and valuable concerns. As per your concerns, we already mentioned some possible confounding factors which could be explored in future. Thanks again.

**R5-14:** Page 09: Furthermore, if an individual is forced to be refrained from smoking in the workplace, it would ultimately lead to quit smoking in the home also. Give suitable references here

**Authors:** Thank you very much for your valuable concerns. As per your concerns, we provided a suitable citation in order to support this statement. Thanks again for understanding.

**R5-15:** Discussion needs more streamlining. It should begin with the key highlights of the paper and each highlight could be discussed in separate paragraph with reference to other studies in the literature, possible reasons and speculations or differences within studies.

**Authors:** Thank you very much for your valuable suggestion. As per your suggestions, we have updated the discussion section to make the potential mechanism clear in the revised manuscript. Thanks again for understanding.

**R5-16:** Conclusion looks a bit repetitive of what has been said before. A more crisp and clear messaging would help.

**Authors:** Thanks again for your valuable suggestion. As per your suggestions, we updated this section and focused on some possible policy implications here.

**Dear Reviewer#5**

Thank you very much for your encouragement, kind guidance, and support in helping the authors improve the manuscript.

We are truly grateful for your time and support. Kind Regards,

The Authors of the manuscript.

**Reviewer (R6)**

This study examines factors associated with smoke-free policies at home and in workplace using the 2017 Global Adult Tobacco Survey (GATS) in India. Additionally, the study examines the correlations between secondhand smoke exposure and smokefree policies. The variables were selected from the literature; therefore, the findings were mostly consistent with the existing literature. Nevertheless, this is the first kind of study in a country with the second largest of tobacco users in the world. The main results show that substantial number of people in the country are not covered by complete smokefree policies which is significant to inform policy initiatives. As such, it provides an added value to the literature. Further, the authors appear to be responsive to earlier reviews. With the above said, here are few suggestions:

**R6-1:** Consider creating a third paragraph for the introduction, starting with "Several studies have been conducted ...."

**Authors:** Thank you for your suggestion. As per your suggestion, we have created a new paragraph from the given statement. Thanks again for understanding.

**R6-2:** The study's aim is still not well-articulated in the paragraph #2.

**Authors:** Thank you very much for asking this question and giving us an opportunity to improve the revised manuscript. As per your concerns, we are now explained more clearly the objective of this study. Thanks again for understanding.

**R6-3:** In section 2.1, did you examine that the data you excluded were not significantly different from the analytic samples.

**Authors:** Thank you very much for raising this question. In this work, we have excluded some observations, like don’t know, refused, and missing values during the analysis. We think that these observations are not statistically significant. As you’re your concerns, we have added a flowchart for the selection of samples in the revised manuscript (**See in Figure 1**). Thanks again for understanding.

**R6-4:** In section 2.4, add the name of the manufacturer and the City and country in a parenthesis after "STATA version 12 (...., ....)

**Authors:** Thank you very much for your valuable comments. As per concerns, we added STATA version along with developer names as well as name of the manufacturer and the City and country in order to reduce the results. Thanks again for understanding.

**R6-5:** In reporting the results (section 3.2), can you report OR, CI, and P-value?

**Authors:** Thank you very much for your valuable suggestion. As per your suggestion, we have added the value of OR with its CI and p-value and explained more clearly in the section 3.2 of the revised manuscript. Thanks again for understanding.

**R6-6:** Please note that this is a cross-sectional study; therefore, the results should not be interpreted as establishing a causation, instead of association.

**Authors:** Thank you very much for your comment. Yes, this is a cross-sectional study. As per your suggestions, we have fixed it in the revised manuscript. Thanks again for understanding.

**R6-7:** Key limitations of the study are missing, particularly biases such as recall and social desirability. The cross-sectional data by itself is a limitation

**Authors:** Thank you very much for your comment. As per your comments, we have added section as “Strength and limitations” in the revised manuscript. Thanks again for understanding.

**R6-8:** The minor issues involve attention to details ---

1) Place comma (,) before "respectively"

2) Delete "h" on the third line under section 4 Discussion

3) Consider removing grid on tables 2 and 3

**Authors:** We highly appreciate your valuable comments. As per your concerns, all points are addressed carefully and updated the revised manuscript. Thanks again for understanding.

**Dear Reviewer#6**

Thank you very much for your encouragement, kind guidance, and support in helping the authors improve the manuscript.

We are truly grateful for your time and support. Kind Regards,

The Authors of the manuscript.
