# Supplementary material for: Smoke-free status of homes and workplaces among Indian people: Evidence from Global Adult Tobacco SurveyData-2016/2017
Source: PLoS One. 2023 Feb 23;18(2):e0282138. doi: 10.1371/journal.pone.0282138 (PMC9949653; doi:10.1371/journal.pone.0282138)
Supplement: Attachment — Submitted filename: R6-Rebuttal-Ver1.docx [file pone.0282138.s013.docx]

**Authors’ Response to Reviewers**

**Manuscript ID: PONE-D-20-41034R6**

**Title: Smoke-free status of homes and workplaces among Indian people: Evidence from Global Adult Tobacco Survey Data-2016/2017**

**Dear Respected Editor-in-Chief,**

We would like to express our gratitude and thanks to the reviewer and the editor for their useful suggestions for improving the manuscript. We have revised the manuscript accordingly. Thank you once again and looking forward to the final acceptance in your esteemed journal.

**Journal Requirements:**

Please review your reference list to ensure that it is complete and correct. If you have cited papers that have been retracted, please include the rationale for doing so in the manuscript text, or remove these references and replace them with relevant current references. Any changes to the reference list should be mentioned in the rebuttal letter that accompanies your revised manuscript. If you need to cite a retracted article, indicate the article’s retracted status in the References list and also include a citation and full reference for the retraction notice.

**Authors:** Thank you very much for giving us to improve the revised manuscript. We have carefully checked the reference list and observed that all reference was correct. We do not update or remove any paper. We read the whole manuscript very carefully and tried to fix some grammatical errors and marked its highlighted. Thanks again for understanding.

**Dear Editor**

Thank you very much for your encouragement, kind guidance, and support in helping the authors improve the manuscript.

We are truly grateful for your time and support. Kind Regards,

The Authors of the manuscript.
