# Supplementary material for: Smoke-free status of homes and workplaces among Indian people: Evidence from Global Adult Tobacco SurveyData-2016/2017
Source: PLoS One. 2023 Feb 23;18(2):e0282138. doi: 10.1371/journal.pone.0282138 (PMC9949653; doi:10.1371/journal.pone.0282138)
Supplement: Attachment — Submitted filename: R7-Rebuttal-Ver1.docx [file pone.0282138.s014.docx]

**Authors’ Response to Reviewers**

**Manuscript ID: PONE-D-20-41034R7**

**Title: Smoke-free status of homes and workplaces among Indian people: Evidence from Global Adult Tobacco Survey Data-2016/2017**

**Dear Respected Editor-in-Chief,**

We would like to express our gratitude and thanks to the reviewer and the editor for their useful suggestions for improving the manuscript. We have revised the manuscript accordingly. Thank you once again and looking forward to the final acceptance in your esteemed journal.

**Academic Editors:**

**AE1:** Kindly edit the abstract to reflect the change from smoke free policies to smoke free status.

**Authors:** Thank you very much for your valuable suggestions and giving us to improve the revised manuscript. As per your suggestions, we have changed the words from “smoke free policies” to “smoke free status” in the abstract of the revised manuscript. Thanks again for understanding.

**AE2:** I recommend that the manuscript should be reviewed by a professional proofreader.  The authors should attach a letter a of from the proof reader when submitting the revisions.

**Authors:** Thank you very much for your valuable concerns. As per your concerns, our revised manuscript has been reviewed by a professor cambridge proofreader and also attached a letter a of from the proof reader during on line submission system. Thanks again for understanding.

**Dear Editor**

Thank you very much for your encouragement, kind guidance, and support in helping the authors improve the manuscript.

We are truly grateful for your time and support. Kind Regards,

The Authors of the manuscript.
